# Ambipolar conjugated ladder polymers by room-temperature Knoevenagel polymerization†

**DOI:** 10.1039/d4sc03222e

**Published:** 2024-06-21

**Authors:** Lingli Zhao, Zeng Wu, Hanwen Qin, Guangxiong Bin, Junxiang Gao, Weixuan Zeng, Yan Zhao, Huajie Chen

**Affiliations:** a Key Laboratory of Environmentally Friendly Chemistry and Applications of Ministry of Education, Key Laboratory of Polymeric Materials and Application Technology of Hunan Province, College of Chemistry, Xiangtan University Xiangtan 411105 P. R. China chenhjoe@xtu.edu.cn; b Laboratory of Molecular Materials and Devices, Department of Materials Science, Fudan University Shanghai 200438 P. R. China zhaoy@fudan.edu.cn; c Zhangjiang Laboratory Shanghai 201210 P. R. China zengwx@zjlab.ac.cn

## Abstract

Two soluble conjugated ladder polymers (cLPs), decorated with multiple electron-poor species (*i.e.*, cyano groups, fused pentagons, and N-heterocyclic rings), have been synthesized from the newly developed tetraketo-functionalized double aza[5]helicene building blocks using a single-step Knoevenagel polycondensation strategy. This facile approach features mild conditions (*e.g.*, room temperature) and high efficiency, allowing us to quickly access a nonalternant ladder-like conjugated system with the *in situ* formation of multicyano substituents in the backbone. Analysis by ^1^H NMR, FT-Raman, and FT-IR spectra confirms the successful synthesis of the resulting cLPs. The combination of theoretical calculations and experimental characterizations reveals that the slightly contorted geometry coupled with a random assignment of *trans*- and *cis*-isomeric repeating units in each main chain contributes to improving the solubility of such rigid, multicyano nanoribbon systems. Apart from outstanding thermal stability, the resulting cLPs exhibit attractive red fluorescence, excellent redox properties, and strong π–π interactions coupled with orderly face-on packing in their thin-film states. They are proven to be the first example of ambipolar cLPs that show satisfactory hole and electron mobilities of up to 0.01 and 0.01 cm^2^ V^−1^ s^−1^, respectively. As we demonstrate, the Knoevenagel polycondensation chemistries open a new window to create complex and unique ladder-like nanoribbon systems under mild reaction conditions that are otherwise challenging to achieve.

## Abstract 2

Two soluble cyanated ladder-like polymers featuring a nonalternant conjugated structure and desirable ambipolar charge-transport properties were synthesized through a room-temperature Knoevenagel polycondensation strategy.

## Graphical abstract

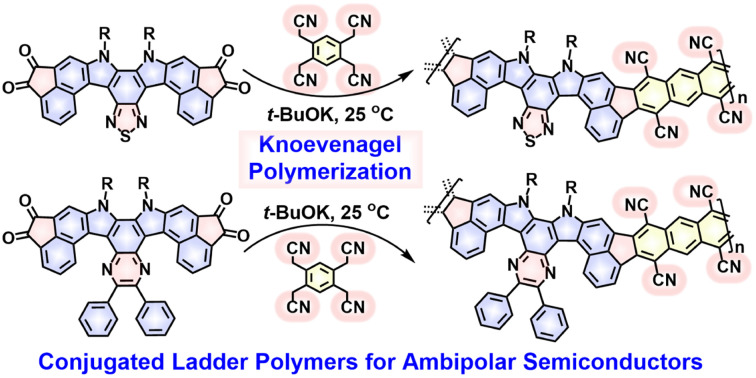

## Introduction

Conjugated ladder polymers (cLPs) are a unique family of ribbon-like macromolecules, in which all the fused-rings in the main chain are linked together by multiple-stranded bonds.^1–4^ Compared with conventional single-stranded conjugated polymers, the fused-ring constitution of cLPs can induce a significantly improved backbone rigidity, inhibiting the free torsional motion of aromatic units along the main-chain.^5,6^ These unique structural characteristics make it possible to generate outstanding thermal/chemical stability, strong inter-chain organization, high intra-chain carrier mobility, and extraordinary photo-/electro-luminescence properties.^7–9^ They are regarded as the perfect targets for various functional applications, and are already adopted as semiconductor layers in organic light-emitting diodes,^10^ organic field-effect transistors (OFETs),^11–16^ organic thermoelectrics,^17,18^ organic lasers,^19,20^ and lithium ion batteries.^21,22^ Despite these amazing prospects, development of structurally defined cLPs is still extremely challenging due to the scarcity of effective synthetic strategies and poor solubility.^23^

**Fig. 1:**
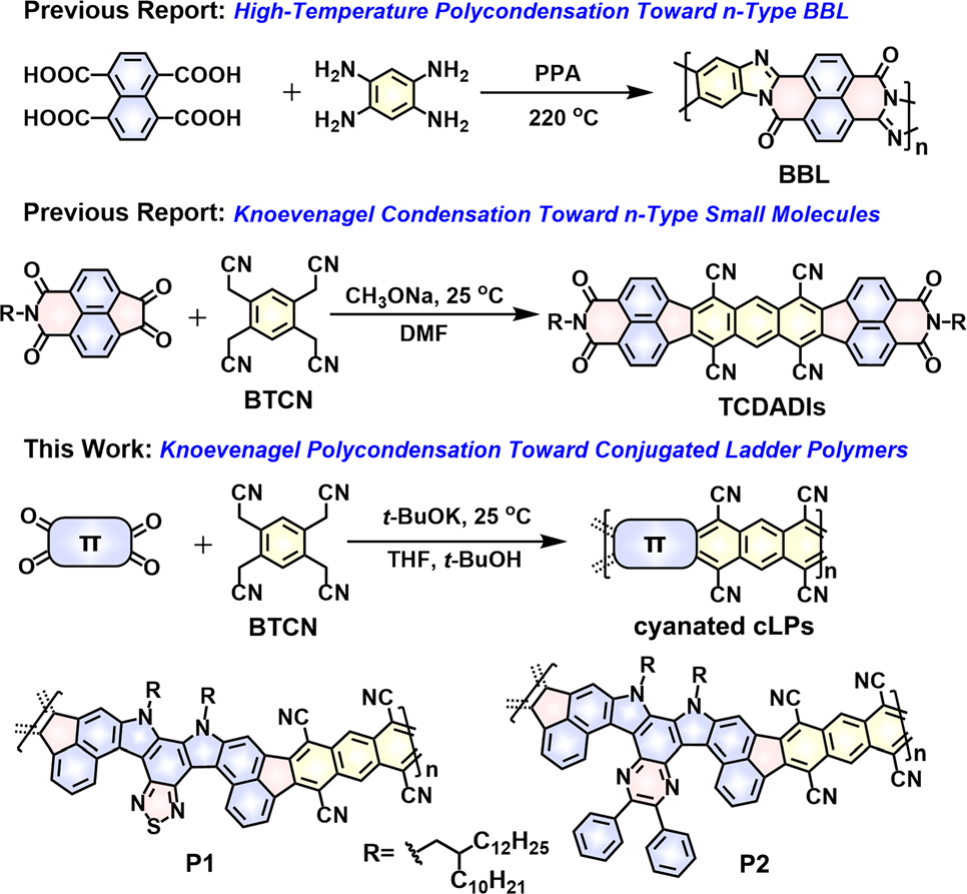
The well-known BBL synthesized from a single-step high-temperature polycondensation route.^39^ Room-temperature Knoevenagel condensation strategies toward generation of the known n-type small molecules (TCDADIs)^51^ and the two novel conjugated ladder polymers (P1 and P2) studied in this work.

Current synthetic strategies toward generation of soluble cLPs can be divided into two distinct approaches,^24^ of which post-polymerization annulation represents the most widely used one.^25,26^ This two-step strategy firstly creates ladder type structures by accessing a single-stranded conjugated polymer bearing suitable functional groups, followed by the intramolecular ring-closure reactions in which the functional groups are quantitatively cyclized to form the additional strand of bonds.^23^ Since the report of the first soluble cLP (*i.e.*, ladder conjugated poly(*p*-phenylene), LPPP) in 1991,^27^ there has been an ever-growing interest in this stepwise technique, thereby providing a wide scope of monomeric building blocks and applicable intramolecular ring-closure reactions, including Scholl coupling,^14^ electrophilic cyclization,^27–31^ photocyclization,^25,32,33^ Schiff base condensation,^34,35^ and olefin ring-closing metathesis.^36,37^ The other strategy for the preparation of cLPs is a single-step ladderization route that involves a simultaneous formation of a two-stranded bond between comonomers.^38^ Compared with the two-step strategy, this approach is more facile, only involving one-step condensation of one or more multifunctional monomers *via* polycondensation reactions^15–18,39,40^ or Diels–Alder polymerizations.^41,42^ One of the most heavily explored ladder polymers, *i.e.*, BBL (Fig. 1), was synthesized by a single-step polycondensation of 1,4,5,8-tetracarboxynaphthalene and 1,2,4,5-tetraaminobenzene in polyphosphoric acid (PPA) at temperatures even up to 220 °C.^39^ Despite investigation for over 50 years, the application of the single-step ladderization strategy to synthesize cLPs is rather limited due to the scarcity of ideal multifunctional monomers, limited synthetic methods, harsh reaction conditions (*e.g.*, high temperature), and poor solubility for the resulting cLPs in common solvents.

As a versatile polymerization strategy, recently the Knoevenagel condensation reaction has been adopted to build numerous cyanovinylene-linked conjugated polymers^43–46^ and 2D covalent-organic frameworks (COFs).^47–50^ Unlike classic metal-catalyzed coupling polymerizations, such as Stille and Suzuki coupling reactions, the Knoevenagel polycondensation, in most cases, takes place under mild reaction conditions, and eliminates the necessity for high temperature and any expensive metal catalysis. For example, by using this technique, synthesis of cyanovinylene-linked COF even can be realized at room temperature without adding any catalyst.^50^ Despite these inspiring progresses, the application of this polymerization technique to construct cLPs has never been documented so far. Such cLPs decorated with multicyano substituents would have even higher electron affinities compared to the monocyanovinylene-linked polymers, while confronting with a huge challenge in improving their solubility. Recently, we have demonstrated the feasibility of constructing high-mobility n-type small molecules, named tetracyanodiacenaphthoanthracene diimides (TCDADIs, Fig. 1),^51^ through the adoption of a one-step fourfold Knoevenagel condensation between a commercially available tetrafunctional 2,2′,2′′,2′′′-(benzene-1,2,4,5-tetrayl)tetraacetonitrile (BTCN) and another diketo-functional aromatic imide building block. Drawing inspiration from the previous progresses, here we have a particular interest in applying this technique to access π-extended ladder structures by reacting BTCN with a tetrafunctional ketone-based monomer (Fig. 1). Furthermore, development of novel single-step ladderization routes to generate cLPs with novel architecture and fantastic properties is highly desirable.

**Scheme 1:**
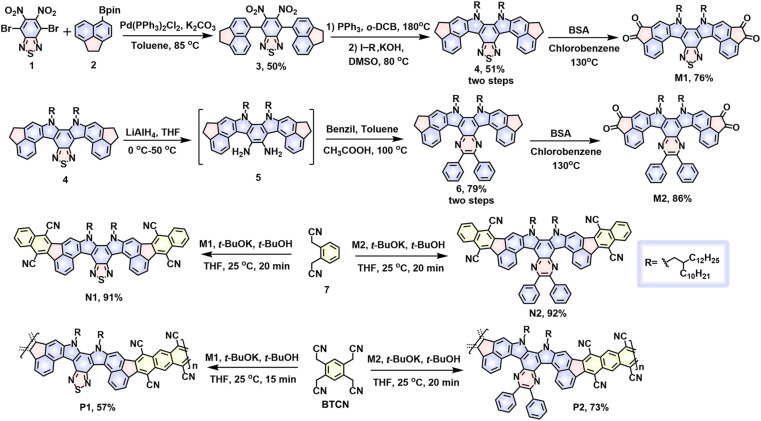
Synthetic strategies to contorted double aza[5]helicene model compounds (N1 and N2) and their conjugated ladder polymers (P1 and P2).

Given the considerations above, herein we have designed and synthesized two novel tetraketo-functionalized double aza[5]helicene building blocks (*i.e.*, thiadiazole-embedded M1 and pyrazine-embedded analogue M2, see Scheme 1), which, respectively, are employed as the tetrafunctional monomer to copolymerize with BTCN*via* a single-step Knoevenagel polycondensation reaction, giving two novel soluble cyanated cLPs (P1 and P2, Fig. 1). This facile approach was found to be rather highly efficient under room temperature, which allows us to access novel cLPs with a distorted nonalternant nanoribbon structure, together with the *in situ* introduction of multicyano substituents during a short reaction time. The main challenge of our molecular design is how to resolve the solubility of such rigid, multicyano nanoribbon systems. Firstly, installation of two long branched alkyl chains (*i.e.*, 2-decyltetradecyl group) in both tetraketo-bearing monomers is considered. Secondly, both monomers are of an axisymmetric character and particularly furnished with double aza[5]helicene-like architecture, which is induced by the steric hindrance effect of *ortho*-fused thiadiazole or pyrazine units in the fjord regions. Owing to these structural characteristics of both tetraketo-bearing monomers, the target cLPs are contorted and possess a random assignment of *trans*- and *cis*-isomeric repeating units in each main chain. With this unique backbone feature, the resulting cLPs can be expected to weaken π–π interactions effectively, and thus, provide more solubility benefits than similar sized, coplanar nanoribbons.^14,52^ In addition, the novel conjugated ladder polymers, P1 and P2, described in this contribution are of particular interest, since they are the first soluble cyanated cLPs featuring multiple cyclopenta-fused nonalternant repeating units and N-heterocyclic rings. The use of these electron-poor species (*i.e.*, cyano groups, fused pentagons, thiadiazole and/or pyrazine rings) is expected to significantly improve the electron affinity of the resulting materials, which would render them desirable electron-transporting properties. In combination with theoretical calculations and experimental techniques, the structures and properties of P1 and P2 were comparatively studied. As expected, the ambipolar transport behavior of both polymer films was experimentally proven, and well correlated with their strong redox behaviors in both oxidation and reduction processes.

## Results and discussion

Scheme 1 describes the synthetic routes of the target ladder polymers and their model compounds. The synthesis of the two tetraketo-functionalized monomers, M1 and M2, started from a Suzuki coupling reaction between the compound 2 and commercially available 4,7-dibromo-5,6-dinitrobenzo[*c*][1,2,5]-thiadiazole, affording compound 3 in 50% yield on a gram scale. Specifically, the starting material, compound 2, was obtained according to the literature method.^53,54^ Treatment of compound 3 with triphenylphosphine at 180 °C furnished a key intermediate, double aza[5]helicene, which directly reacted with 11-(iodomethyl)tricosane to give a 2-decyltetradecyl-substituted double aza[5]helicene 4 in 51% yield over two steps. With this thiadiazole-containing 4 ready, one tetraketo-functionalized monomer, thiadiazole-embedded M1, was readily prepared in the presence of benzeneseleninic anhydride (BSA). Previous reports have demonstrated that the thiadiazole rings in the polycyclic hydrocarbons possess high reaction activity that has been heavily adopted to construct diverse N-heterocyclic derivatives.^55^ With this in mind, compound 4 was further treated with LiAlH_4_ to form a diamine intermediate, subsequently reacting with benzil *via* a ketoamine condensation reaction to furnish a pyrazine-bearing analogue 6. Next, further oxidation of compound 6 with BSA produced another tetraketo-functionalized monomer, pyrazine-embedded M2. In this case, an isolated yield of 68% was obtained over three steps from compound 4. The solubility of both tetrafunctional monomers, M1 and M2, is excellent in most common solvents, which enables next solution-phase synthesis of the target cLPs *via* the Knoevenagel polycondensation strategy.

Prior to the preparation of target polymers, firstly we aimed at synthesizing two model small molecules, N1 and N2, to validate the feasibility of the strategy. Under the catalysis of *t*-BuOK (5.0 eq.) at 25 °C, Knoevenagel condensations between 2,2′-(1,2-phenylene)diacetonitrile and tetraketo-bearing monomers (M1 or M2) in THF were accomplished in 20 min, affording the ring-closure molecules, N1 or N2, in high isolated yields of 91 and 92%, respectively. During the reaction, a solution of *t*-BuOK in *tert*-butanol was added dropwise using an additional syringe with vigorous stirring. Despite the mild temperature and short time, the synthesis of model small molecules was proven to be highly efficient without the appearance of undesirable side reactions (*e.g.*, intermolecular cross-linking), exhibiting a great promise of exploring the synthesis of cLPs.

Encouraged by the above results, the synthesis of P1 and P2 was further implemented under similar reaction conditions. To a THF solution of tetraketo-bearing monomers (M1 or M2) and BTCN monomer was added *t*-BuOK (5.0 eq.) at 25 °C and stirred for 15 and 20 min for P1 and P2, respectively, followed by quenching with water. Upon adding *t*-BuOK, the reaction mixture quickly turned from pale red to light red, and gradually to dark brown with strong red fluorescence, implying a quick decrease of band gap upon polymerization. The solid samples were isolated *via* precipitation and filtration, and further purified with Soxhlet extraction. The black powders, P1 and P2, were recovered from chlorobenzene extracts in 57% and 73% yields, respectively. For such reaction systems, prolongation of reaction time led to an increase of insoluble polymers owing to quickly improved molecular weights. Thanks to their distorted geometries and multiple 2-decyltetradecyl substituents, both polymers are soluble in most hot aromatic solvents (*e.g.*, toluene, chlorobenzene, and *o*-dichlorobenzene) and show good film-forming ability, thereby offering a feasibility for the effective characterization of chemical structures and optoelectronic properties *via* a solution-processable technique. The excellent thermal stability of both P1 and P2 was demonstrated by TGA measurements (Fig. S1†), in which the decomposition temperatures (determined from 5% weight loss) reach 413 and 399 °C, respectively.

**Fig. 2:**
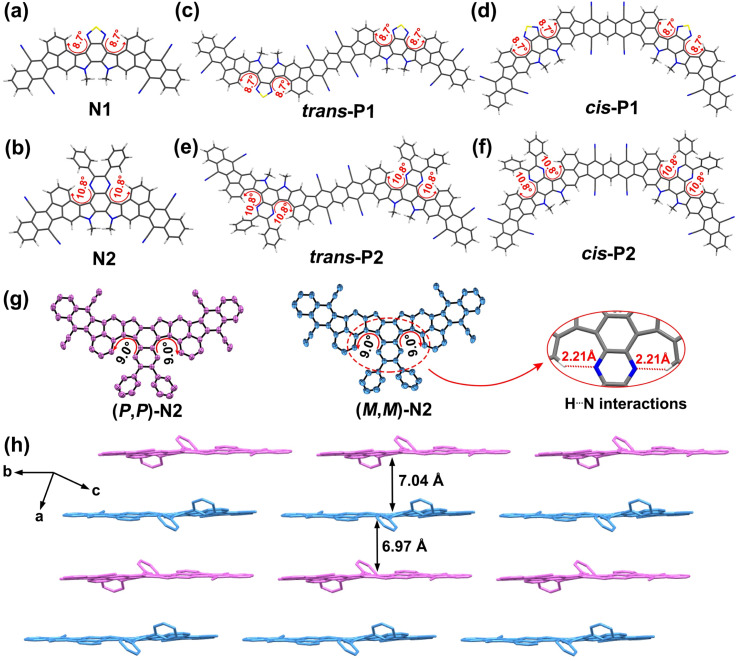
Ground-state structures of small-molecule models, N1 (a) and N2 (b), and polymeric dimer models, *trans*-/*cis*-P1 (c and d) and *trans*-/*cis*-P2 (e and f), calculated at the DFT//B3LYP/def2-SVP level. (g) X-ray crystallographic structures of enantiomers (*P*,*P*)-N2 and (*M*,*M*)-N2, and geometric details for twisted dihedral angles (9.0°) and H⋯N interactions (2.21 Å). (h) Molecular packing arrangement of racemic N2. Hydrogen atoms and 2-decyltetradecyl side-chains are omitted for clarity.

Analysis by high-temperature (150 °C) gel-permeation chromatography verifies the successful preparation of both polymers, whose number-average molecular weights (*M*_n_) and polydispersity indexes (PDIs) are determined to be 14.02 kDa/1.55 for P1 and 21.75 kDa/1.77 for P2 (Fig. S2†). The number of repeating units estimated from the *M*_n_ values is around 10 and 14 for P1 and P2, respectively. The different polymerization degrees can be associated with their degree of backbone distortion which is highly correlated with their solubility. To clarify this further, the structural optimizations were conducted at the DFT//B3LYP/def2-SVP level based on the methyl-substituted calculation models (Fig. 2a–f and S4a, b, S5†). Given the random appearance of *trans*- and *cis*-isomeric repeating units in each polymer backbone, both *trans*- and *cis*-dimers of P1 and P2 were separately taken into account.

For the thiadiazole-embedded π-systems (*i.e.*, N1, *trans*- and *cis*-P1), the twisted dihedral angle of 8.7° is observed in the double aza[5]helicene subunits (Fig. 2a, c, d and S4a†), while their pyrazine-embedded analogues (*i.e.*, N2, *trans*- and *cis*-P2) show a slight increase of backbone distortion, featuring larger torsional angles of 10.8° (Fig. 2b, e, f and S4b†). As shown in Fig. S5,† the increase of heterocyclic ring size from the five-membered thiadiazole unit to the six-membered pyrazine unit leads to a reduced H⋯N distance of 2.13 Å relative to the thiadiazole-embedded π-systems (2.19 Å). Obviously, the decrease of H⋯N distance induces an improved steric repulsive force in the fjord regions, thereby achieving the improved torsional degree in the pyrazine-bearing π-systems. In addition, the phenyl substituents in the pyrazine-containing π-systems are positioned out of the molecular plane of the adjacent pyrazine unit, and feature the torsional dihedral angles of 43.20–43.44° (Fig. S5†), respectively. In comparison with P1, the larger torsional angles combined with the appearance of multiple free-torsional phenyl substituents in P2 contribute to more effectively suppressing inter-chain aggregation during solution polymerization.^14^ This is a key point to improving the solubility of P2, thereby achieving a remarkable increase in both polymerization degree and synthetic yield relative to P1.

To unambiguously confirm the geometry of these fused-ring compounds, a single crystal of N2, suitable for X-ray analysis, was fortunately accessed by slow evaporation of a mixture solution, and the detailed crystal parameters are summarized in Table S1.† We also tried our best to access a suitable single crystal of N1, but failed. The analysis indicates that two enantiomers (*P*,*P*)-N2 and (*M*,*M*)-N2 with a sightly distorted geometry are observed in the crystal of N2 (Fig. S3†), whose twisted dihedral angles presented in the fjord regions are determined to be 9.0° (Fig. 2g and S4c, d†), very close to the DFT-optimized results (10.8°, Fig. 2b). The short H⋯N distance (2.21 Å, Fig. 2g), defined by the pyrazinyl N and the adjacent phenyl H atom, induces the backbone distortion of two aza[5]helicene subunits. In addition, two phenyl substituents are positioned out of the plane of the fused-ring system (Fig. 2h). This structural feature theoretically could weaken the intermolecular interactions, and thus offers more solubility benefits relative to the similar sized, plane nanoribbons. The molecules of N2 adopt a parallel columnar packing pattern, showing an alternating arrangement of (*P*,*P*)-N2 and (*M*,*M*)-N2 (Fig. 2h). However, two bulky 2-decyltetradecyl groups sterically isolate N2 molecules within the face-to-face arrangement (Fig. S3†), and consequently, the interplane distances between the adjacent racemic dimers reach around 7.0 Å (Fig. 2h and S4e†). This loose packing structure can be expected to increase the solubility and fluorescence of the materials.

**Fig. 3:**
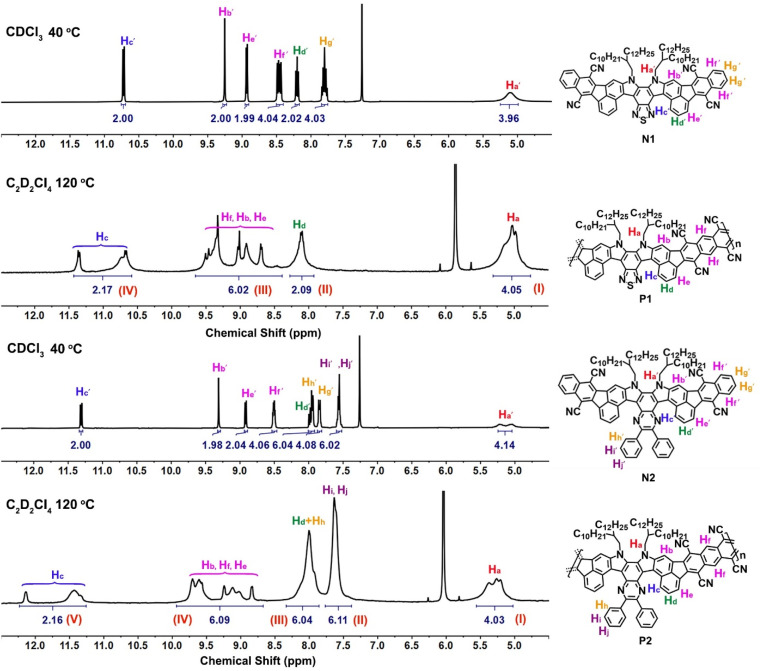
^1^H NMR spectra of model small molecules (N1 and N2) and their polymers (P1 and P2).

The success of the polymerization was also confirmed by NMR spectroscopic study (Fig. 3), in which the ^1^H NMR signals of N1 and N2 are sharp and well-defined for each aromatic proton. The doublet peaks at around 11.0 ppm belong to the inner edge protons H_c′_ of the so-called fjord region, while the other four phenyl protons, located near cyano substituents, give the corresponding singlet (H_b′_), doublet (H_e′_), and multiple peaks (H_f′_) at around 8.4–9.4 ppm. After the polymerization to form the more rigid polymers, all the peaks of ^1^H NMR spectra are broadened, similar to the other known ladder polymer systems;^14,29,30^ moreover, those of partial aromatic protons (*e.g.*, H_b_, H_c_, H_e_, and H_f_) are shifted downfield and changed into more complex signals compared to N1 and N2. For example, the H_c_ protons located in the fjord regions give two very broad peaks at around 10.5–11.5 ppm for P1 and 11.2–12.5 ppm for P2, whereas the characteristic peaks of H_b_, H_e_, and H_f_ at around 8.3–10.0 ppm become more complex and undefined. The presence of line-broadening and low-resolution signals mainly originates from the conformation variation in the fjord regions and the random assignment of *trans*- and *cis*-isomeric repeating units in each main chain. Despite their complex resonances, the characteristic peaks at 4.3–12.5 ppm can be assigned to the I–IV and I–V regions for P1 and P2 (Fig. 3), respectively. In addition, the integral area ratios between each region are quite consistent with the characteristic protons of P1 and P2, thereby confirming their chemical structures. The formation of polymers was further confirmed by FT-IR spectral study (Fig. S6†), in which the C≡N stretching band at around 2220 cm^−1^ is detectable for each polymer. However, the FT-IR spectra of P1 and P2 reveal the presence of two novel carbonyl signals at around 1735–1774 cm^−1^, quite distinct from the values presented in both comonomers M1 and M2 (around 1718–1720 cm^−1^, Fig. S6†). These signals may belong to the carbonyl end-groups and unreacted carbonyl defects in each main-chain, indicating that both polymers have relatively few single-bond linkages in the π-conjugated systems.

To validate the characteristics of conjugated nanoribbons, P1 and P2 were also characterized by Raman spectroscopy and DFT calculations (Fig. S7†). The FT-Raman spectra of P1 and P2 are very similar, indicating that varying N-heterocycles in the fjord regions shows a negligible influence on their FT-Raman spectra. The main characteristic bands (*i.e.*, G and D regions) expected for graphene nanoribbons are detected for both P1 (G: 1598 cm^−1^; D: 1405 cm^−1^) and P2 (G: 1598 cm^−1^; D: 1404 cm^−1^). These Raman lines are well reproduced by the DFT-calculations, where the simulated results for each *cis*- and *trans*-dimer model match well with their experimental data (Fig. S7†). It is noteworthy that weaker bands at around 1150 cm^−1^ to 1300 cm^−1^ are observed for the two polymers in both experiment and calculation (Fig. S7†), which can be assigned to the bending vibration along the fjord segments. An insightful inspection of the corresponding nuclear displacement patterns is illustrated in Fig. S8–S11,† and effectively supports the assessment of these Raman characteristic bands, especially the G and D features of P1 and P2. The observed Raman signatures combined with the DFT-simulated lines further confirm the successful synthesis of conjugated ladder polymers.

**Fig. 4:**
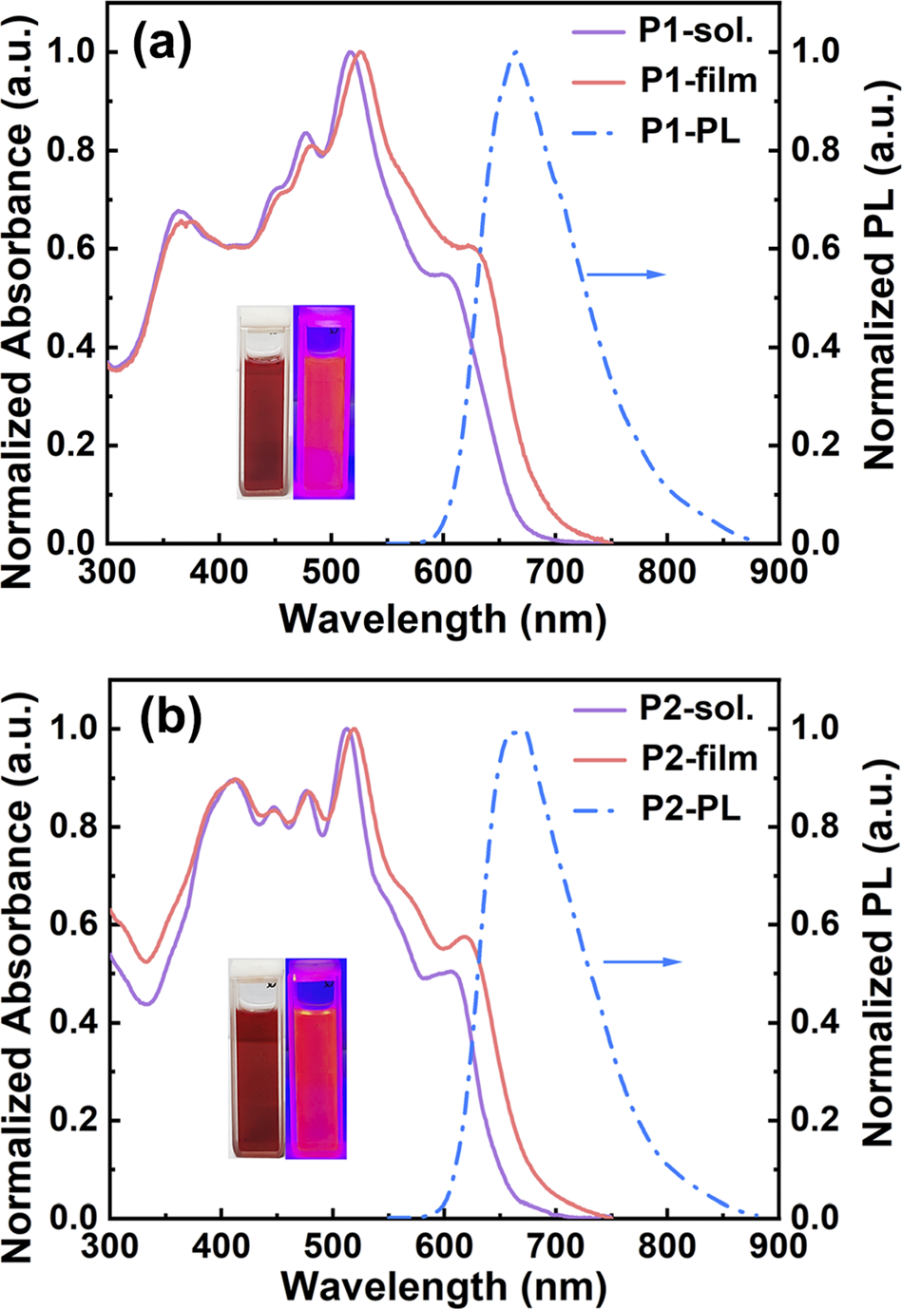
Normalized absorption spectra of P1 (a) and P2 (b) in both chlorobenzene solution (purple lines) and as thin films (red lines). Normalized PL spectra (blue lines) in chlorobenzene solution.

To study the optical properties, all the model compounds and polymers were examined by UV-vis absorption and fluorescence spectroscopy, and the detailed data are collected in Table S2.† In chlorobenzene, the absorption spectra of both N1 and N2 present well-resolved vibronic structures in the long-wavelength regions (Fig. S12†), indicating the rigidity of double aza[5]helicene-like aromatics. This absorption feature is commonly observed in many planar polycyclic aromatic hydrocarbons.^56–59^ The 0–0 transition peaks for N1 and N2 are located at 511 and 507 nm, respectively, with the corresponding molar absorption coefficients (*ε*) of 1.25 × 10^5^ and 0.99 × 10^5^ M^−1^ cm^−1^ (Fig. S12†), respectively. The blue shift of the 0–0 transition peaks from N1 to N2 implies slightly weakened electron communications, which is caused by installing slightly weaker acceptors (*i.e.*, pyrazine rings) instead of thiadiazole rings. Despite the existence of clear structural variation in their fjord regions, both N1 and N2 show almost identical optical band gaps (*E*^opt^_g_) of around 2.30 eV, as estimated from the low-energy onsets (around 539 nm, Fig. S12†). This observation suggests little influence on the *E*^opt^_g _values by varying from thiadiazole rings to pyrazine rings. In comparison with model compounds, two ladder polymers, P1 and P2, show a significant broadening of the light-capturing bands, featuring the low-energy onsets of 676 and 667 nm and the *E*^opt^_g _values of 1.83 and 1.86 eV (Table S2† and Fig. 4), respectively. The *E*^opt^_g _values of both polymers are thus reduced by around 0.5 eV compared to N1 and N2, which can be attributed to the elongation of π-conjugation and the attendance of extra electron-donating/-withdrawing units. In addition, the film absorption bands of P1 and P2 are much broader than those in solution (Fig. 4). This red-shift phenomenon can be explained by the occurrences of orderly packing or interchain aggregation in the solid-state films.^60^ Interestingly, clear absorption shoulders at around 580–650 nm are detectable in both dilute chlorobenzene solution and film spectra of P1 and P2 (Fig. 4 and S13†). Moreover, their normalized absorption spectra measured in different solution concentrations are almost identical to each other (Fig. S13†), featuring a similar absorption shoulder at around 580–650 nm. The results indicate that the absorption shoulder can be attributed to the weak charge transfers within the π-backbones of P1 and P2 and well clarified by the simulated absorption spectra as discussed below (Fig. S17–S20†). In chlorobenzene, both N1 and N2 emit a light-yellow photoluminescence (PL) with the emission peaks of 533 and 528 nm (Fig. S12†), respectively. Owing to the effective elongation of π-conjugation, a deep-red PL solution is observed for both P1 and P2, whose PL peaks are remarkably shifted to 665 and 669 nm (Fig. 4), respectively. The PL quantum yields (PLQYs) examined in chlorobenzene are 54% for N1, 51% for N2, 45.9% for P1, and 55.3% for P2 (Table S2 and Fig S14†). The PLQY values observed for both polymers are attractive for various functional applications like fluorescent dyes, biosensors, and organic light-emitting transistors.

To understand the typical absorption bands in depth, time-dependent DFT (TD-DFT) calculations were further conducted at the PBE0/def2-SVP level based on the DFT-optimized model structures (Fig. 2a–f). Note that the absorption maxima at around 516.4 nm for N1 and 508.7 nm for N2 originate from the electron transition from HOMO to LUMO (Fig. S15 and S16†), while other strong peaks at around 457.6 nm for N1 and 452.5 nm for N2 are attributed to the HOMO → LUMO+2 transition. These transitions could be assigned to localized excitation arising from the π-conjugation through the ladder-like backbones. Noticeably, the absorption spectra of small molecules (N1 and N2) can be well reproduced by the theoretical calculations. Compared with that of N1 (516.4 nm), the absorption maxima of *trans*- and *cis*-P1 exhibit only 5–7 nm red-shifts, peaking at around 523 and 521 nm (Fig. S17 and S18†), respectively. The former mainly results from the contribution of the S_3_ state (HOMO → LUMO+1, 73.1% and HOMO−1 → LUMO+2, 15.4%, Fig. S18†), while the latter can be associated with the combined contributions of the S_3_ state (HOMO → LUMO+1, 75.6% and HOMO−1 → LUMO+2, 11.8%, Fig. S17†) and S_4_ state (HOMO−1 → LUMO+1, 51.7% and HOMO → LUMO+2, 40.4%, Fig. S17†). Unlike N1, new absorption bands in the long-wavelength regions of *trans*- and *cis*-P1 are observed (Fig. S17 and S18†), corresponding to the intramolecular charge transfer within π-backbones (mainly contributed by HOMO → LUMO or HOMO−1 → LUMO). The electron transitions for these new absorption bands all involve their LUMOs that are mainly localized in the segments of tetracyanoanthracene and the adjacent pentagon rings (Fig. S17 and S18†). Although varying from *trans*- to *cis*-conformations shows a weak influence on the spectrum shape, *trans*-P1 presents around 50 nm red-shifts in both maximum absorption peak and low-energy onset than those of *cis*-P1. Considering that *trans*- and *cis*-isomeric repeating units are randomly embedded in P1 during polymerization, data fitting was thus performed to predict the absorption spectrum by combining the simulated results of *trans*- and *cis*-P1 (Fig. S21a†). The fitted absorption bands agree better with the experimental result of P1, comparing with either the *trans*- or *cis*-dimer model (Fig. S17 and S18†). Similar to the observations of *trans*- and *cis*-P1, varying the backbone conformation leads to a small change in the spectrum shape of *trans*- and *cis*-P2, while featuring different absorption tails, as verified by the TD-DFT calculation details (Fig. S19 and S20†). Consequently, the fitted absorption spectrum of P2 is also in line with that of experimental spectra (Fig. S21b†).

**Fig. 5:**
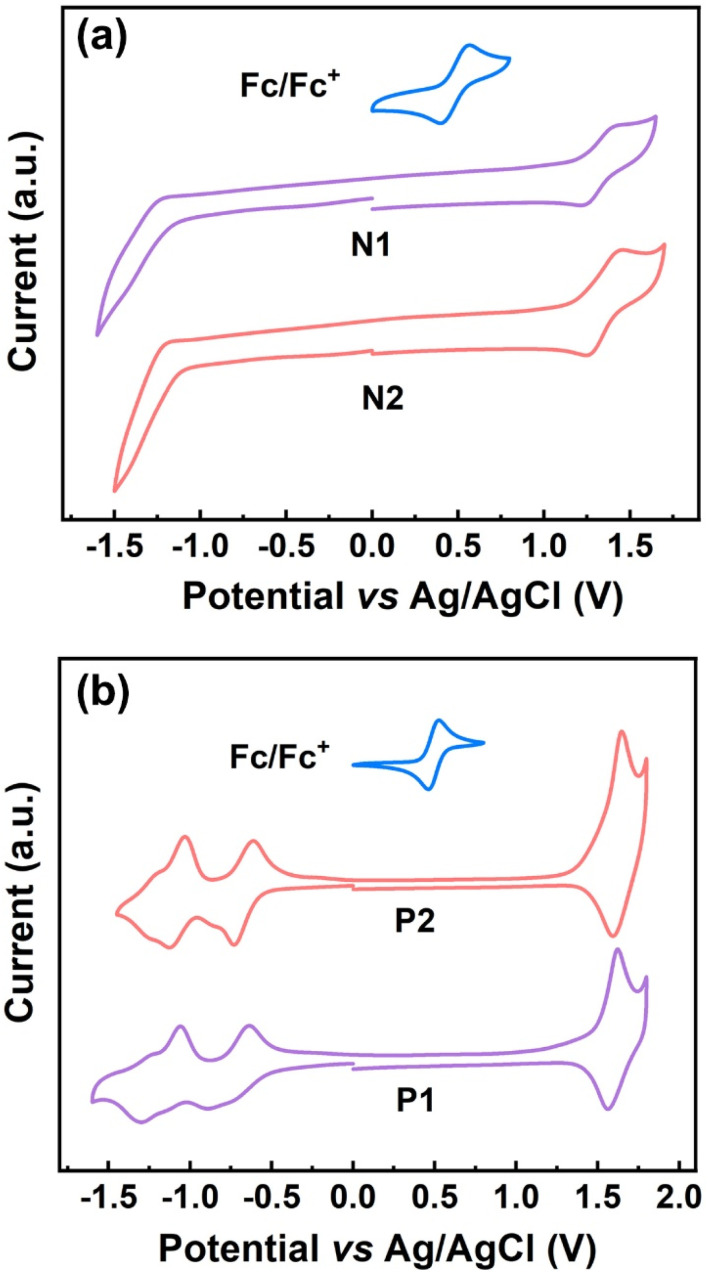
(a) CV curves of N1 and N2 measured in chloroform solution; (b) CV curves of P1 and P2 measured in acetonitrile solution.

The conjugated ladder polymers decorated with multiple electron-poor species should be promising electron-transport materials due to their highly electron-poor character. To verify this, the electrochemical properties of P1, P2, and their model compounds were evaluated by cyclic voltammetry (CV). Two model compounds, N1 and N2, exhibit one strong quasi-reversible oxidation wave but a weak reduction process, with the first-onset oxidation potentials (*E*^onset^_ox_) of 1.22 V (*vs.* Ag/AgCl) for N1 and 1.21 V (*vs.* Ag/AgCl) for N2 (Table S2† and Fig. 5a). As for both P1 and P2, the redox waves in both oxidation and reduction processes become more remarkable simultaneously (Fig. 5b), on account of the installation of larger π-conjugations and more electron-poor species (*i.e.*, cyano groups, fused pentagon rings, and heterocycles) relative to their model compounds. The first-onset oxidation and reduction potentials of P1 appear at 1.46 and −0.52 V (*vs.* Ag/AgCl), featuring the HOMO and LUMO energies of −5.88 and −3.90 eV, and the electrochemical band gap of 1.98 eV, respectively. As illustrated in Table S2,† both HOMO and LUMO energies of P2 are very close to those of P1, which is due to the little variation of electron-deficient capacity from the thiadiazole to the pyrazine rings that are embedded in P1 and P2, respectively. In combination with their low-lying HOMO/LUMO energies, simultaneous appearance of strong oxidation and reduction processes would be beneficial for their use as the ambipolar materials in OFET applications.^61^

**Fig. 6:**
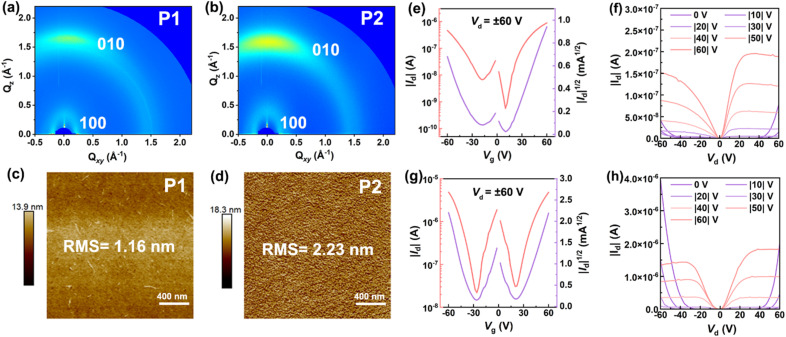
(a and b) 2D-GIWAXS diffraction patterns and (c and d) AFM images of P1 and P2 films; (e) transfer and (f) output curves of P1-based OFETs; (g) transfer and (h) output curves of P2-based OFETs.

To characterize the molecular packing and film crystallinity of both polymers, we carried out the measurements of grazing incidence wide-angle X-ray scattering (GIWAXS). The 2D-GIWAXS patterns of both P1 and P2 films were found to show an anisotropic halo at *q* ≈ 1.5 Å^−1^ and a strong scattering spot in the *Q*_*z*_ direction (Fig. 6a and b), indicating that their polymer sheets can form an alignment of face-on packing and partial crystallization phase in addition to the amorphous phase. To gain more information about the packing of both polymers, we further plotted the in-plane and out-of-plane 1D-GIWAXS curves. From in-plane 1D-GIWAXS images we observe one strong (100) scattering peak at around 0.23 and 0.25 Å^−1^ for P1 and P2 (Fig. S22†), respectively. The *d* spacing of this peak is calculated to be around 27.31 Å for P1 and 25.13 Å for P2 by using the equation of *d* = 2π/*q*, which is assumed to be the lamellar stacking distance.^62^ Clearly, the lamellar spacing of P2 is smaller than that of P1. By carefully looking at out-of-plane 1D-GIWAXS patterns, the (010) peak positions of P1 and P2 are located at 1.69 and 1.62 Å^−1^ (Fig. S22†), respectively, and their π–π packing distances are determined to be 3.72 and 3.88 Å (Table S3†), respectively. Overall, despite the presence of the wave-like main-chain structure, the films of P1 and P2 are still able to form an orderly inter-chain organization, quite different from the amorphous character observed in other known ladder polymer systems.^12,14^ Furthermore, atomic force microscopy (AFM) images of P1 and P2 films were found to show different morphological surfaces. As for P1, a featureless surface filled with irregular polymer fibers is observed (Fig. 6c), while P2 exhibits a more regular morphology coupled with an orderly distribution of interconnected polymeric nodes (Fig. 6d). The morphological character observed for P2, by contrast, is more attractive for charge transport than that of P1.

To quantify their charge-transport properties, the OFET devices with a bottom-gate/bottom-contact architecture (Fig. S23a†) was fabricated by deposition of P1 or P2 film onto the OTS-modified silicon wafers (see the details in the ESI†). All the transfer and output curves of the devices were collected in an N_2_ glovebox. When source–drain voltages of ±60 V were applied, typical “V” shape transfer profiles are clearly visible for both P1- and P2-based transistors (Fig. 6e and g), implying that they are ambipolar semiconductors. The average hole and electron mobilities (*μ*_h_/*μ*_e_) of 3 × 10^−3^/4 × 10^−3^ cm^2^ V^−1^ s^−1^ are extracted from 30 P1-based transistors (Fig. S23b and Table S4†), featuring the highest *μ*_h_/*μ*_e_ values of 8 × 10^−3^/9 × 10^−3^ cm^2^ V^−1^ s^−1^, respectively. Compared with P1, a slight improvement of charge-transport ability is observed for the P2-based transistors, and their maximum *μ*_h_/*μ*_e_ values reach 0.01 and 0.01 cm^2^ V^−1^ s^−1^, respectively. When going from P1 to P2, the increasing of molecular weights and more regular surface morphology are responsible for the enhancement of charge transport ability. It should be mentioned that, apart from n-type BBL (*μ*_e_, as high as 0.1 cm^2^ V^−1^ s^−1^),^11^ the known cLPs generally give low mobilities of around 10^−6^ to 10^−3^ cm^2^ V^−1^ s^−1^ when used for OFETs (Table S5†);^12,14–16^ the ambipolar mobilities observed for P2 are quite satisfactory and can be correlated with the orderly face-on packing and partial crystallization in the solid-state films. To the best of our knowledge, they are the first examples of ambipolar cLPs featuring excellent solution-processability and attractive red fluorescence.

## Conclusion

In conclusion, we have demonstrated that the Knoevenagel reaction can be used as a single-step polycondensation technique to construct soluble multicyano-functionalized conjugated ladder polymers from the newly developed tetrafunctional monomers, *i.e.*, tetraketo-bearing double aza[5]helicenes. This versatile annulation chemistry features mild conditions (*e.g.*, room temperature) and high efficiency, offering excellent feasibility of accessing complex nonalternant conjugated nanoribbon systems with the *in situ* formation of multicyano substituents and reasonably high molecular weights. The resulting polymers present high thermal decomposition temperature, attractive red fluorescence, high electron affinity and low-lying HOMO/LUMO energies owing to the multiple electron-poor species (*i.e.*, cyano groups, fused pentagons, and N-heterocycles) furnished in the backbones. They are proven to form an orderly face-on packing orientation in the solid-state films and to be the first example of ambipolar conjugated ladder polymers, showing the highest hole and electron mobilities of up to 0.01 and 0.01 cm^2^ V^−1^ s^−1^, respectively. Our findings demonstrate that efficient Knoevenagel polycondensation chemistries open a new avenue for accessing complex and unique carbon nanoribbon frameworks under mild reaction conditions that are otherwise challenging to achieve. Further extension of this polycondensation strategy toward novel π-conjugated systems and fantastic functional applications is already underway in our laboratory.

## Data availability

The synthetic details, TGA, GPC, FT-IR, crystallographic data, FT-Raman, absorption spectra, OFET device performance, GIWAS, NMR data and computational results are available in the ESI.†

## Author contributions

H. C. designed and conducted the project; L. Z., G. B. and J. G. performed the material synthesis and characterization; Z. W. and Y. Z. collected the data of OFET performance, AFM and GIWAXS; W. Z. and H. Q. carried out the theoretical calculations; H. C., L. Z. and W. Z. wrote this manuscript; H. C., W. Z. and Y. Z. supervised the project. All authors have reviewed and approved the manuscript.

## Conflicts of interest

The authors declare no conflict of interest.

## Supplementary Material

SC-015-D4SC03222E-s001

SC-015-D4SC03222E-s002
