# Supplementary material for: Ambipolar conjugated ladder polymers by room-temperature Knoevenagel polymerization
Source: Chem Sci. 2024 Jun 21;15(29):11594–603. doi: 10.1039/d4sc03222e (PMC11268504; doi:10.1039/d4sc03222e)
Supplement: SC-015-D4SC03222E-s001 [file SC-015-D4SC03222E-s001.pdf]

Electronic Supplementary Material (ESI) for Chemical Science.
This journal is © The Royal Society of Chemistry 2024
Evaluation Only. Created with Aspose.Pdf. Copyright 2002-2014 Aspose Pty Ltd.

Supplementary information

Ambipolar Conjugated Ladder Polymers by Room-Temperature Knoevenagel Polymerization

Lingli Zhao,[a] ‡ Zeng Wu,[b]‡ Hanwen Qin,[a] Guangxiong Bin,[a] Junxiang Gao,[a] Weixuan Zeng,*[c] Yan Zhao,*[b] Huajie Chen*[a]

a L. Zhao, H. Qin, G. Bin, J. Gao, Prof. H. Chen
Key Laboratory of Environmentally Friendly Chemistry and Applications of Ministry of Education and Key Laboratory of Polymeric Materials and Application Technology of Hunan Province, College of Chemistry, Xiangtan University
Xiangtan 411105, P. R. China.
E-mail: chenhjoe@xtu.edu.cn

b Z. Wu, Prof. Y. Zhao
Laboratory of Molecular Materials and Devices, Department of Materials Science, Fudan University
Shanghai 200438, P. R. China.
E-mail: zhaoy@fudan.edu.cn

c Dr. W. Zeng
Zhangjiang Laboratory
Shanghai, 201210, P. R. China.
E-mail: zengwx@zjlab.ac.cn

‡ These authors contributed equally to this work.

Evaluation Only. Created with Aspose.Pdf. Copyright 2002-2014 Aspose Pty Ltd.

Table of Contents

S1. Measurement S3
S2. Synthesis of materials S3
S3. TGA Data of Both Polymers S5
S4. High-Temperature GPC Data of Both Polymers S5
S5. Single Crystal X-ray Crystallographic Data and DFT-Optimized Structures S5
S6. FT-IR Spectrum of Compounds S8
S7. FT-Raman Data of P1 and P2 .... S8
S8. Photophysical and Electrochemical Properties S13
S9. Quantum Chemical Calculations S14
S10. GIWAXS Data of Polymer Films S18
S11. Transistor Devices Fabrication and Measurement S19
S12. ^{1}H and ^{13}C NMR of Compounds S22
S13. Computational Atomic Coordinate S38
S14. Reference S59

Evaluation Only. Created with Aspose.Pdf. Copyright 2002-2014 Aspose Pty Ltd.

S1. Measurement

The ^{1}H and ^{13}C NMR spectra for each small molecule and high-temperature (373K) ^{1}H NMR spectra for both polymers were collected on a Bruker AVANCE 400, and tetramethylsilane (TMS) was used as the interval standard. Fourier transform infrared spectroscopy (FT-IR) was recorded on a NICOLET is50 spectrometer in the range from 600 to 4000 cm^{-1}. Thermogravimetric analysis (TGA) was recorded on a TGA Q50 V20.13 Build 39 with a heating rate of 20 °C min^{-1} from 25 to 600 °C. High-resolution mass spectra (MALDI-TOF-MS) were measured on a Bruker Biflex III MALDI-TOF Bruker instrument. High-resolution electrospray ionization (ESI) mass spectra were collected on an Orbitrap Fusion Lumos instrument. High-temperature (150 °C) gel-permeation chromatography were conducted on an Agilent GPC instrument (Polymer Labs PL 220 system), and 1,2,4-trichlorobenzene and polystyrene were used as the eluent and standard sample, respectively. UV−Vis absorption spectra of model small molecules in chlorobenzene solution, and the polymers in chlorobenzene and as thin films casted onto the quartz glass were measured on an Agilent Cary 60 UV-Vis spectrophotometer. The photoluminescence (PL) spectra for each sample in chlorobenzene solution were collected on a Hitachi F-4600 spectrophotometer. The absolute PL quantum yields for each sample in chlorobenzene solution were measured on an Edinburgh Instruments (FS5) with a SC-30 Integrating Sphere by using chlorobenzene as the reference. Cyclic voltammetry (CV) was performed on an electrochemistry workstation (CHI660E, Chen hua Shanghai) using a three-electrode cell system. To examine model small molecules (N1 and N2), the electrochemical cell contains a glassy carbon electrode, a Pt wire, and an Ag/AgCl (KCl, Sat'd) electrode. To collect the CV curves of both polymers, a Pt disk coated with a layer of polymer film, a Pt wire, and an Ag/AgCl (KCl, Sat'd) electrode, were used as the working electrode, counter electrode, and reference electrode, respectively. Anhydrous and Ar-saturated tetrabutylammonium hexafluorophosphate ($TBAPF_6$, 0.1 M) solutions in chloroform and in acetonitrile were used as the supporting electrolytes to collect the CV curves of small molecules and polymers, respectively. Raman spectra were recorded on a Renishaw inVia instrument equipped with Nd-YVO4 laser (532 nm) in the range of 1100~1700 cm^{-1}. The surface morphology for both polymer films was characterized using an atomic force microscope (AFM, Park XE7) in a tapping mode. The molecular packing and film crystallinity of the polymer films were characterized by grazing incidence wide-angle X-ray scattering (GIWAXS), at Shanghai Synchrotron Radiation Facility, Shanghai Institute of Applied Physics, Chinese Academy of Science.

S2. Synthesis of materials

Materials. The key monomer, 2,2′,2′′,2′′′-(Benzene-1,2,4,5-tetrayl)tetraacetonitrile (BTCN), was obtained from Alfa Aesar, and the other chemicals were obtained from Chem Greatwall. The starting material, 4,7-dibromo-5,6-dinitrobenzo[*c*][1,2,5]thiadiazole (1), was purchased from Macklin. Compound 2 was synthesized following the reported method [1-2].

Synthesis of compounds 3. Compound 1 (500 mg, 1.3 mmol), compound 2 (912 mg, 3.26 mmol), and $Pd(PPh_3)_2Cl_2$ (90 mg, 0.13 mmol) were dissolved in 12 mL of toluene, followed by the adding of K_2CO_3 aqueous solution (2.0 M, 4 mL). The reaction solution was stirred at 85 °C for 16 h under argon atmosphere. After cooling down to room temperature, the organic phase was separated by extraction with CH_2Cl_2, and then dried over $MgSO_4$. After the removal of solvent under reduced pressure, the residue was purified by silica-gel column chromatography to afford an orange solid (petrol ether /CH_2Cl_2, V: V = 2:1, 345 mg, yield 50 %). ^{1}H NMR (400 MHz, $CDCl_3$) δ (ppm): 7.59 (t, *J* = 8.0 Hz, 2H), 7.47–7.43 (m, 4H), 7.39 (d, *J* = 8.0 Hz, 2H), 7.16 (dd, *J* = 16.0, 8.0 Hz, 2H), 3.51 (s, 8H). ^{13}C NMR (100 MHz, $CDCl_3$) δ (ppm): 153.52, 153.34, 149.58, 149.54, 146.90, 146.81, 143.77, 143.71, 139.34, 129.79, 129.73, 129.63, 129.26, 129.16, 129.10, 128.96, 123.33, 123.16, 120.43, 120.05, 119.73, 119.11, 119.08, 30.53, 30.48. HRMS: m/z [M]$^+$ calcd for ($C_{30}H_{18}N_4O_4S$) 530.1043; found 530.1041.

Synthesis of compounds 4. To a 25 mL tube, compound 3 (500 mg, 0.94 mmol), triphenylphosphine (4.94 g, 18.85 mmol), and 5 mL of *o*-dichlorobenzene were added. The reaction solution was stirred at 180 °C for 12 h under argon atmosphere. After cooling down to room temperature, the reaction mixture was poured into 100 mL of ethanol. The brown solid sample was collected by filtration, and used directly for the next step without further purification. To a 25 mL two-necked flask, the as-obtain filter residue (439 mg, 0.94 mmol), 11-(iodomethyl)tricosane (2.63 g, 5.65 mmol), and 10 mL of dried dimethyl sulfoxide were added. Under argon atmosphere, the reaction solution was stirred at 80 °C for 10 min, followed by the adding of KOH (634 mg, 11.31 mmol) and stirring for 16 h. After cooling down to room temperature, the organic phase was extracted by petrol ether and washed with brine, and then dried over $MgSO_4$. After the removal of solvent under reduced pressure, the residue was purified by silica-gel column chromatography to afford a brown yellow solid (petrol ether, 548 mg, 51 % yield over two step). ^{1}H NMR (400 MHz, $CDCl_3$) δ (ppm): 10.25 (d, *J* = 8.0 Hz, 2H), 7.78 (t, *J* = 8.0 Hz, 2H), 7.57 (s, 2H), 7.43 (d, *J* = 4.0 Hz, 2H), 4.71 (t, *J* = 8.0 Hz, 4H), 3.57 (s, 8H), 1.95–1.90 (m, 2H), 1.32–0.86 (m, 92H). ^{13}C NMR (100 MHz, $CDCl_3$) δ (ppm): 149.59, 145.45, 143.77, 140.89, 136.19, 132.15, 128.17, 127.23, 123.40, 118.98, 118.65, 112.91, 106.62, 52.12, 37.35, 31.96, 31.90, 31.09, 30.04, 29.64, 29.37, 22.72, 22.68, 14.15. HRMS: m/z [M]$^+$ calcd for ($C_{78}H_{114}N_4S$) 1138.8758; found 1138.8747.

Synthesis of compounds M1. To a 25 mL tube, compound 4 (500 mg, 0.44 mmol), benzeneseleninic anhydride (BSA) (948 mg, 2.63 mmol), and 10 mL of chlorobenzene were added. The reaction solution was stirred at 130 °C for 2 h under argon atmosphere. After cooling down to room temperature, the organic phase was extracted by CH_2Cl_2 and washed with brine, and then dried over $MgSO_4$. After the removal of solvent under reduced pressure, the residue was purified by silica-gel column chromatography to afford a red solid (petrol ether /CH_2Cl_2, V: V = 1:2, 399 mg, yield 76 %). ^{1}H NMR (400 MHz, $CDCl_3$) δ (ppm): 10.96 (dd, *J* = 12.0, 4.0 Hz, 2H), 8.52 (s, 2H), 8.26 (d, *J* = 8.0 Hz, 2H), 8.18 (t, *J* = 8.0 Hz, 2H), 4.92 (s, 4H), 1.94 (s, 2H), 1.29–0.83 (m, 92H). ^{13}C NMR (100 MHz, $CDCl_3$) δ (ppm): 188.89, 188.43, 149.19, 143.35, 139.11, 134.34, 133.96, 129.30, 128.87, 126.90, 126.15, 125.53, 120.84, 114.58, 109.68, 53.25, 38.56, 31.90, 31.80, 29.52, 29.37, 29.30, 22.68, 22.62, 14.12, 14.09. HRMS: m/z [M+H]$^+$ calcd for ($C_{78}H_{107}N_4O_4S$) 1195.8007; found 1195.7994. FT-IR data: $\nu_{C=O}$, 1718 cm^{-1}.

Evaluation Only. Created with Aspose.Pdf. Copyright 2002-2014 Aspose Pty Ltd.

Synthesis of compounds 6. To a 50 mL two-necked flask, compound 4 (500 mg, 0.44 mmol) and 15 mL of THF were added. The reaction solution was stirred at 0 °C for 10 min under argon atmosphere, followed by the adding of $LiAlH_4$ (666 mg, 17.55 mmol). The reaction mixture was stirred at 0 °C for 10 min, then at 50 °C overnight. After cooling down to room temperature, the organic phase was extracted by ethyl acetate and washed with brine, and then dried over $MgSO_4$. After the removal of solvent under reduced pressure, the as-obtained aromatic amine 5 was used directly for the next-step reaction. Under reduced pressure, a mixture of compound 5 (488 mg, 0.44 mmol), benzil (111 mg, 0.53 mmol), 8 mL of toluene, and 8 mL of CH_3COOH were stirred at 100 °C overnight. After cooling down to room temperature, the organic phase was extracted by CH_2Cl_2 and washed with brine, and then dried over $MgSO_4$. After the removal of solvent under reduced pressure, the residue was purified by silica-gel column chromatography to afford a yellow solid (petrol ether /CH_2Cl_2, V: V = 3:1, 445 mg, yield 79 %). ^{1}H NMR (400 MHz, $CDCl_3$) δ (ppm): 10.77 (d, J = 8.0 Hz, 2H), 7.91 (d, J = 8.0 Hz, 4H), 7.62 (s, 2H), 7.57 (t, J = 8.0Hz, 2H), 7.46 (m, 6H), 7.38 (d, J = 8.0 Hz, 2H), 4.77 (d, J = 8.0 Hz, 4H), 3.58 (s, 8H), 1.95–1.92 (m, 2H), 1.27–0.86 (m, 92H). ^{13}C NMR (100 MHz, $CDCl_3$) δ (ppm): 147.09, 145.20, 144.17, 141.86, 140.36, 136.29, 132.16, 130.66, 128.04, 127.86, 127.42, 126.56, 119.64, 119.54, 118.26, 106.71, 51.85, 37.19, 31.93, 31.28, 31.10, 30.00, 29.56, 29.36, 23.76, 22.70, 14.14. HRMS: m/z $[M]^+$ calcd for ($C_{92}H_{124}N_4$) 1284.9826; found 1284.9805.

Synthesis of compounds M2. The synthetic procedure of compound M2 is similar to that of compound M1. On the basis of the reaction mixture of compound 6 (500 mg, 0.39 mmol), BSA (840 mg, 2.33 mmol), and 10 mL of chlorobenzene, an orange solid was prepared and isolated via silica-gel column chromatography (petrol ether /CH_2Cl_2, V: V = 1:2, 449 mg, yield 86%). ^{1}H NMR (400 MHz, $CDCl_3$) δ (ppm): 11.48 (d, J = 8.0 Hz, 2H), 8.56 (s, 2H), 8.20 (d, J = 8.0 Hz, 2H), 7.93–7.87 (m, 6H), 7.57–7.53 (m, 6H), 4.97 (s, 4H), 1.96 (s, 2H), 1.22–0.84 (m, 92H). ^{13}C NMR (100 MHz, $CDCl_3$) δ (ppm): 189.13, 188.70, 149.35, 143.62, 139.92, 139.09, 137.20, 136.79, 134.07, 130.29, 129.23, 128.97, 128.51, 128.05, 127.65, 126.45, 126.35, 120.99, 120.52, 109.67, 52.82, 38.31, 31.87, 31.14, 29.60, 29.43, 29.31, 26.76, 23.38, 22.66, 14.12, 14.07. HRMS: m/z $[M+H]^+$ calcd for ($C_{92}H_{116}N_4O_4$) 1341.9069; found 1341.9053. FT-IR data: $\nu_{C=O}$, 1720 cm^{-1}.

Synthesis of compounds N1. To a 25 mL two-necked flask, M1 (100 mg, 0.084 mmol), compound 7 (26 mg, 0.17 mmol), and 10 mL of THF were added. The reaction solution was stirred at 25 °C under argon atmosphere. Then, 2 mL of *t*-BuOH solution of t-BuOK (47 mg, 0.42 mmol) was added dropwise via syringe. After 20 min, the reaction was quenched by water. The organic phase was extracted by $CHCl_3$ and washed with brine, and then dried over $MgSO_4$. After the removal of solvent under reduced pressure, the residue was purified by silica-gel column chromatography to afford a yellow solid (petrol ether /CH_2Cl_2, V: V = 3:1, 109 mg, yield 91%). ^{1}H NMR (400 MHz, $CDCl_3$) δ (ppm): 10.72 (d, J = 8.0 Hz, 2H), 9.25 (s, 2H), 8.93 (d, J = 8.0 Hz, 2H), 8.46 (dd, J = 16, 8.0 Hz, 4H), 8.20 (t, J = 8.0 Hz, 2H), 7.83–7.78 (m, 4H), 5.10 (s, 4H), 2.13–2.10 (m, 2H), 1.27–0.78 (m, 92H). ^{13}C NMR (100 MHz, $CDCl_3$) δ (ppm): 149.27, 142.05, 140.55, 134.17, 132.83, 131.03, 130.98, 130.91, 130.36, 129.13, 128.99, 128.90, 128.74, 126.10, 125.97, 125.76, 123.93, 122.35, 116.57, 116.00, 114.33, 111.76, 105.49, 105.18, 53.33, 38.84, 31.90, 31.85, 29.71, 29.61, 29.43, 29.31, 22.67, 22.62, 14.12, 14.08. HRMS: m/z $[M]^+$ calcd for ($C_{98}H_{114}N_8S$) 1434.8881; found1434.8863. FT-IR data: ν_{CN}, 2220 cm^{-1}.

Synthesis of compounds N2. The synthetic procedure of compound N2 is similar to that of compound N1. On the basis of the reaction mixture of M2 (100 mg, 0.075 mmol), compound 7 (23 mg, 0.15 mmol), 2 mL of *t*-BuOH solution of *t*-BuOK (42 mg, 0.37 mmol), and 10 mL of THF, an orange solid was prepared and isolated via silica-gel column chromatography (petrol ether /CH_2Cl_2, V: V = 3:1, 108 mg, yield 92%). ^{1}H NMR (400 MHz, $CDCl_3$) δ (ppm): 11.31 (d, J = 8.0 Hz, 2H), 9.31 (s, 2H), 8.92 (d, J = 8.0 Hz, 2H), 8.51 (dd, J = 8.0, 4.0 Hz, 4H), 8.00–7.93 (m, 6H), 7.87–7.82 (m, 4H), 7.59–7.53 (m, 6H), 5.13 (d, 4H), 2.11 (s, 2H), 1.27–0.83 (m, 92H).^{13}C NMR (100 MHz, $CDCl_3$) δ (ppm): 148.86, 142.49, 141.44, 140.73, 139.54, 136.66, 133.98, 133.70, 133.10, 131.07, 131.00, 130.97, 130.53, 129.16, 128.99, 128.69, 128.40, 128.19, 126.78, 126.20, 126.03, 124.86, 122.16, 120.86, 116.66, 116.18, 111.71, 105.46, 105.16, 52.64, 38.49, 31.87, 29.66, 29.49, 23.82, 22.64, 14.11. HRMS: m/z $[M]^+$ calcd for ($C_{112}H_{124}N_8$) 1580.9943; found 1580.9927. FT-IR data: ν_{CN}, 2226 cm^{-1}.

Synthesis of polymer P1. To a 25 mL two-necked flask, M1 (150 mg, 0.125 mmol), BTCN (29.4 mg, 0.125 mmol), and 15 mL of THF were added. The reaction solution was stirred at 25 °C under argon atmosphere. Then, 3 mL of *t*-BuOH solution of *t*-BuOK (70 mg, 0.625 mmol) was added dropwise via syringe. After 15 min, the reaction was quenched by water. The mixture was poured into a solution of ethanol (100 mL) and concentrate HCl (1 mL). After stirring for 20 min, the solid sample was collected by filtration, followed by the further purification via Soxhlet extraction with ethanol, acetone, petroleum ether, and chlorobenzene. The target polymer was separated from the chlorobenzene solution and dried under vacuo, giving a black purple solid (97 mg, 57%). FT-IR data: ν_{CN}, 2220 cm^{-1}. Molecular weights: M_n = 14.02 kDa, M_w = 21.67 kDa.

Synthesis of compounds P2. The synthetic procedure of P2 is similar to that of P1. On the basis of the reaction mixture of M2 (150 mg, 0.112 mmol), BTCN (26.2 mg, 0.112 mmol), 3 mL of *t*-BuOH solution of *t*-BuOK (63 mg, 0.56 mmol), and 15 mL of THF. After 20 min, the reaction was quenched by water, a black purple solid was prepared (122 mg, yield 73%). FT-IR data: ν_{CN}, 2220 cm^{-1}. Molecular weights: M_n = 21.75 kDa, M_w = 38.47 kDa.

Evaluation Only. Created with Aspose.Pdf. Copyright 2002-2014 Aspose Pty Ltd.

S3. TGA Data of Both Polymers

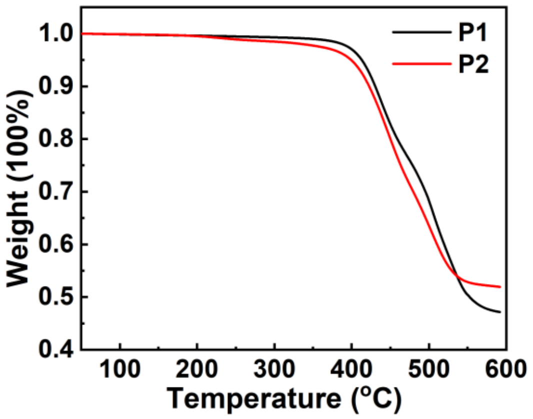

Fig. S1. TGA curves of P1 and P2.

S4. High-Temperature GPC Data of Both Polymers

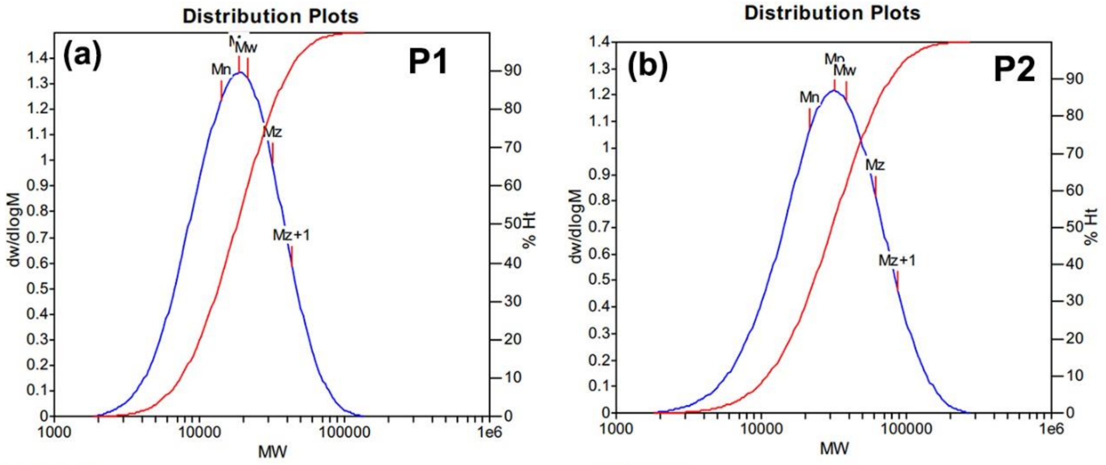

MW Averages (P1)

Peak No	Mp	Mn	Mw	Mz	Mz+1	Mv	PD
1	18595	14018	21668	31791	43519	20245	1.54573

MW Averages (P2)

Peak No	Mp	Mn	Mw	Mz	Mz+1	Mv	PD
1	32149	21750	38475	61330	87858	35345	1.76897

Fig. S2. High-temperature GPC curves of P1 and P2.

S5. Single Crystal X-ray Crystallographic Data and DFT-Optimized Structures

Under room temperature, a light-yellow flaky crystal of N2, suitable for X-ray analysis, was obtained by slow evaporation of the corresponding solution in CH_2Cl_2/toluene/hexane (V: V: V = 1: 0.5: 10). Single-crystal X-ray measurements were conducted on a XtaLAB Synergy R, DW system, HyPix diffractometer. During data collection, the crystal of N2 was kept at 100.00(10) K. The structure of N2 was solved by direct methods using Olex2[3] with the SHELXT structure solution program using intrinsic phasing algorithm[4]. The non-hydrogen atoms were refined anisotropically with the SHELXL[5] refinement package using Least Squares minimisation. In addition, the position of hydrogen atoms was fixed geometrically at the calculated distances and allowed to ride on their parent atoms. The disordered moieties presented in the single crystal were further refined using bond length restraints and displacement parameter restraints. Crystallographic data for N2 has been deposited at the Cambridge Crystallographic Data Center, and the deposition number (CCDC) is 2353054. The crystallographic data of N2 can be downloaded directly from the Cambridge Crystallographic Data Centre via www.ccdc.cam.ac.uk/structures.

Evaluation Only. Created with Aspose.Pdf. Copyright 2002-2014 Aspose Pty Ltd.

Table S1. Crystal data and structure refinement for N2

Identification code	N2
Empirical formula	$C_{112}H_{124}N_8$
Formula weight	1582.18
Temperature/K	100.00(10)
Crystal system	monoclinic
Space group	I2/a
a/Å	15.0671(3)
b/Å	13.6045(3)
c/Å	46.1940(9)
α/°	90
β/°	90.107(2)
γ/°	90
Volume/Å^3	9468.8(3)
Z	4
ρ_{calc}g/cm^3	1.110
μ/mm^{-1}	0.487
F(000)	3408.0
Crystal size/mm^3	0.132 × 0.1 × 0.08
Radiation	Cu Kα (λ = 1.54184)
2Θ range for data collection/°	6.774 to 133.198
Index ranges	-16 ≤ h ≤ 17, -16 ≤ k ≤ 16, -54 ≤ l ≤ 54
Reflections collected	32783
Independent reflections	8304 [R_{int} = 0.0629, R_{sigma} = 0.0542]
Data/restraints/parameters	8304/636/599
Goodness-of-fit on F^2	1.448
Final R indexes [I>=2σ (I)]	R_1 = 0.1186, wR_2 = 0.3478
Final R indexes [all data]	R_1 = 0.1523, wR_2 = 0.3787
Largest diff. peak/hole / e Å^{-3}	0.53/-0.52
CCDC number	2353054

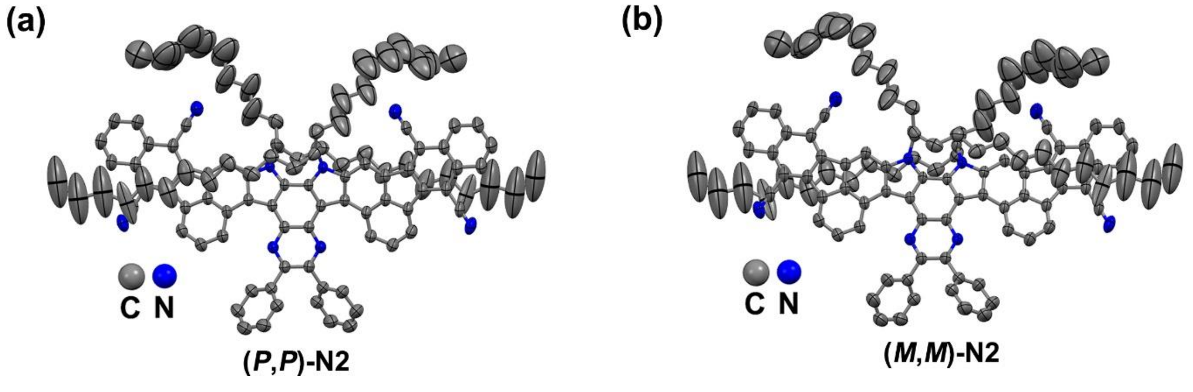

Fig. S3. Thermal ellipsoid plots of enantiomers (*P,P*)-N2 and (*M,M*)-N2 at the 75% probability level, and hydrogen atoms are omitted for clarify.

Evaluation Only. Created with Aspose.Pdf. Copyright 2002-2014 Aspose Pty Ltd.

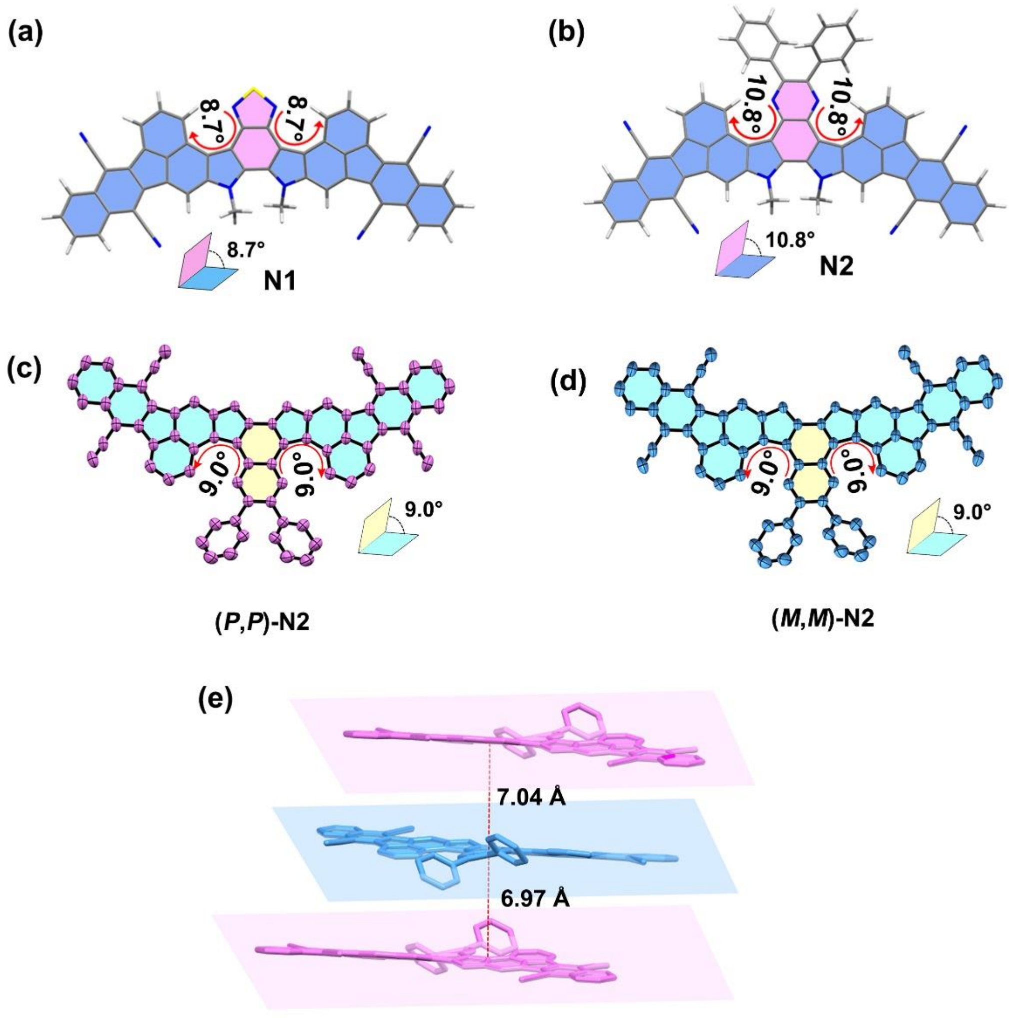

Fig. S4. Twisted dihedral angles extracted from the DFT-optimized structures of (a) N1 and (b) N2. (c, d) Twisted dihedral angles extracted from the single crystal structures of (*P,P*)-N2 and (*M,M*)-N2. (e) The packing distances extracted from the planes of adjacent racemic dimers.

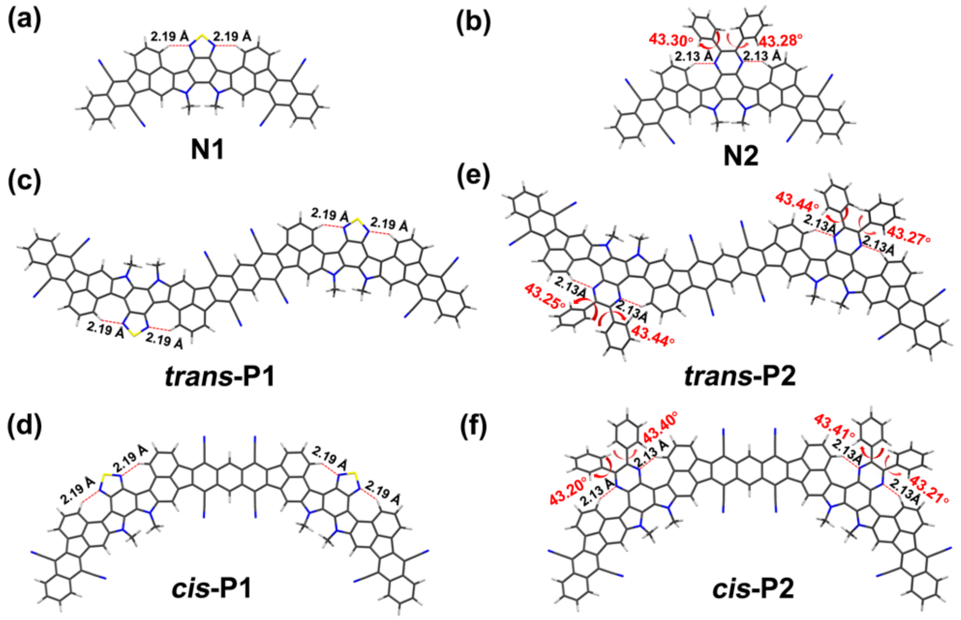

Figure S5. The H···N distances presented in the fjord regions and the twisted angles of the phenyl substituents. All the results are determined from the DFT-calculated ground-state structures.

Evaluation Only. Created with Aspose.Pdf. Copyright 2002-2014 Aspose Pty Ltd.

S6. FT-IR Spectrum of Compounds

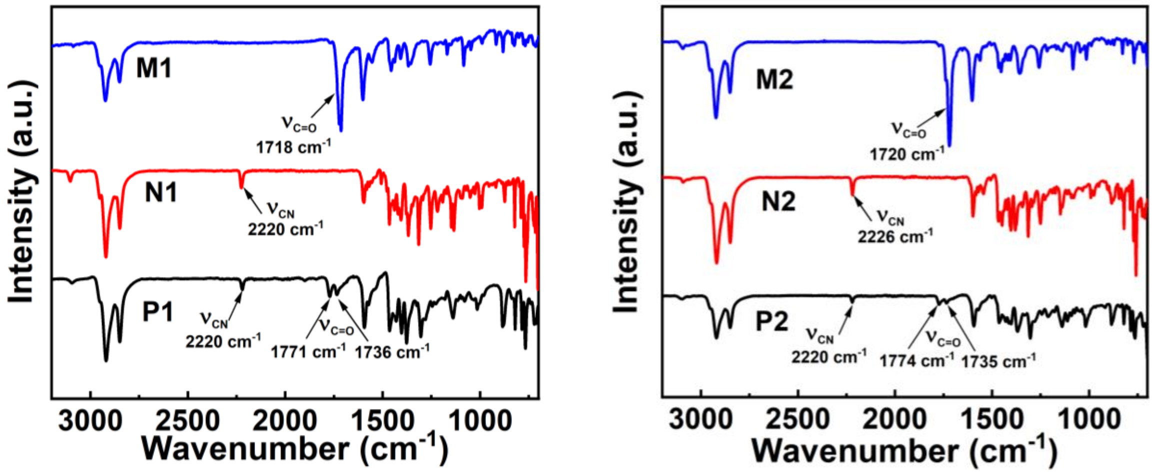

Fig. S6. FT-IR spectra of model compounds (N1 and N2), monomers (M1 and M2), and polymers (P1 and P2).

S7. FT-Raman Data of P1 and P2

The detailed computational methods for the Raman spectrum calculation are provided in followed S9 section.

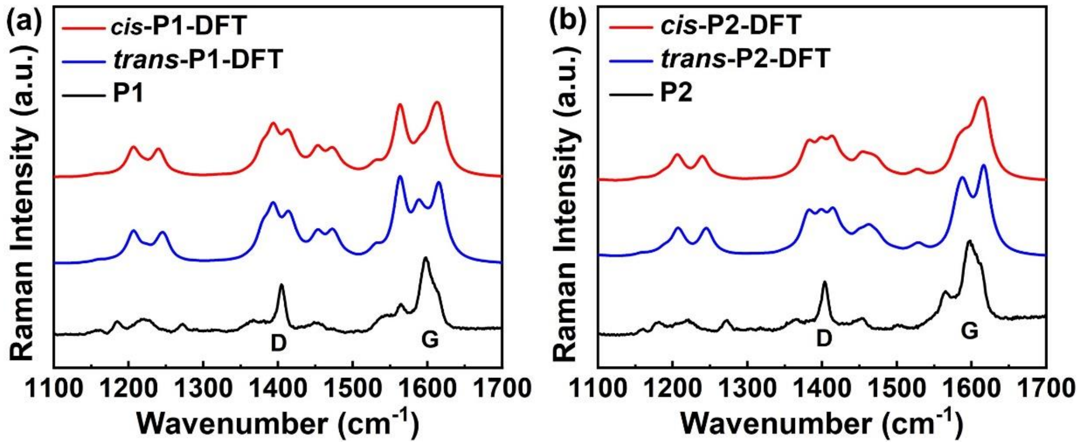

Fig. S7. (a) Raman spectrum of P1 in the solid state and the calculated Raman spectrum of the polymer models (*cis*-P1-DFT and *trans*-P1-DFT). (b) Raman spectrum of P2 in the solid state and the calculated Raman spectrum of the model structures (*cis*-P2-DFT and *trans*-P2-DFT).

Evaluation Only. Created with Aspose.Pdf. Copyright 2002-2014 Aspose Pty Ltd.

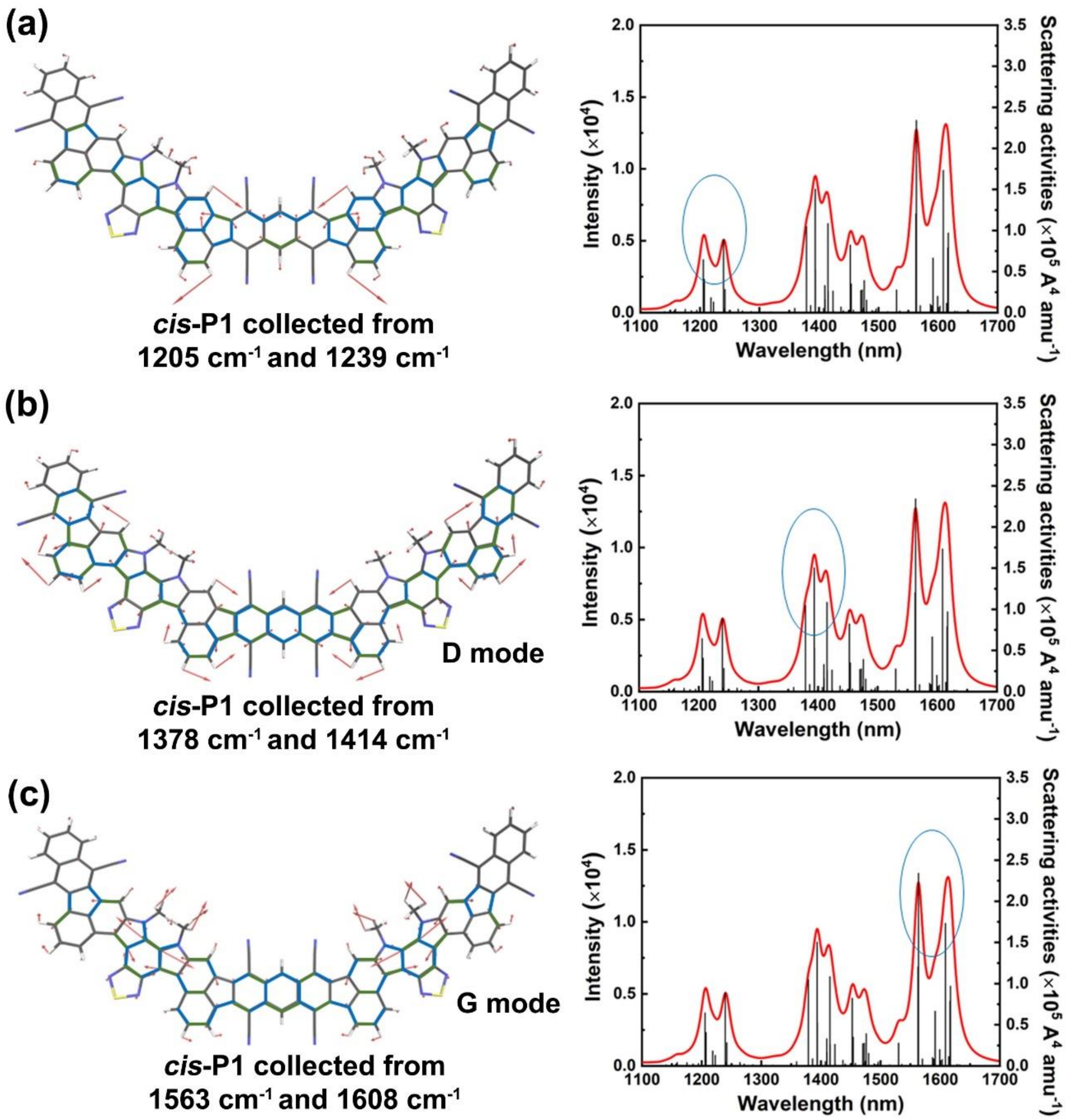

Fig. S8. The calculated normal modes and Raman spectrum of the polymer model *cis*-P1: red arrows represent displacement vectors; bonds between non-hydrogen atoms marked with different colors represent relative stretching (green) or shrinking (blue).

Evaluation Only. Created with Aspose.Pdf. Copyright 2002-2014 Aspose Pty Ltd.

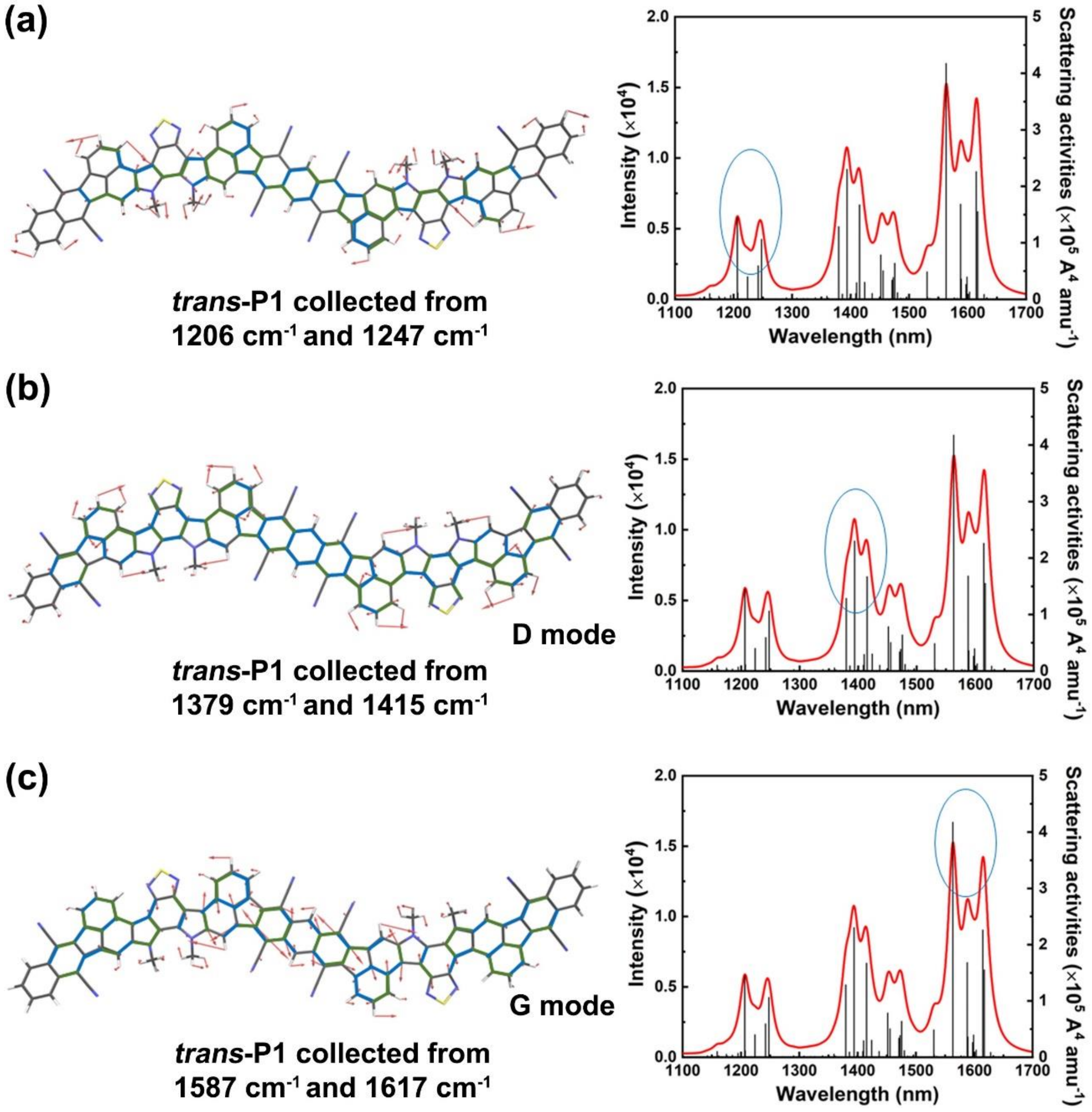

Fig. S9. The calculated normal modes and Raman spectrum of the polymer model *trans*-P1: red arrows represent displacement vectors; bonds between non-hydrogen atoms marked with different colors represent relative stretching (green) or shrinking (blue).

Evaluation Only. Created with Aspose.Pdf. Copyright 2002-2014 Aspose Pty Ltd.

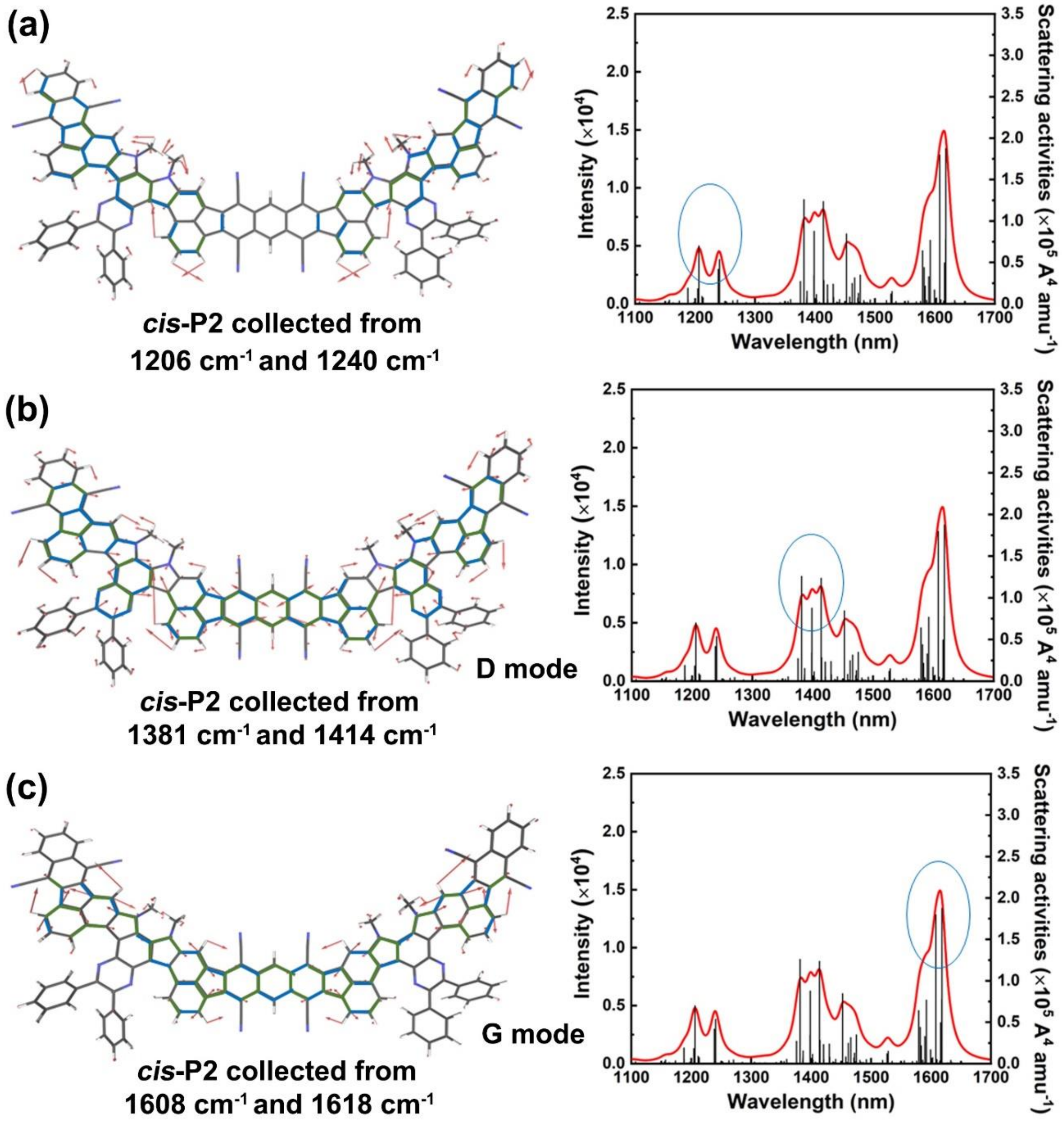

Fig. S10. The calculated normal modes and Raman spectrum of the polymer model *cis*-P2: red arrows represent displacement vectors; bonds between non-hydrogen atoms marked with different colors represent relative stretching (green) or shrinking (blue).

Evaluation Only. Created with Aspose.Pdf. Copyright 2002-2014 Aspose Pty Ltd.

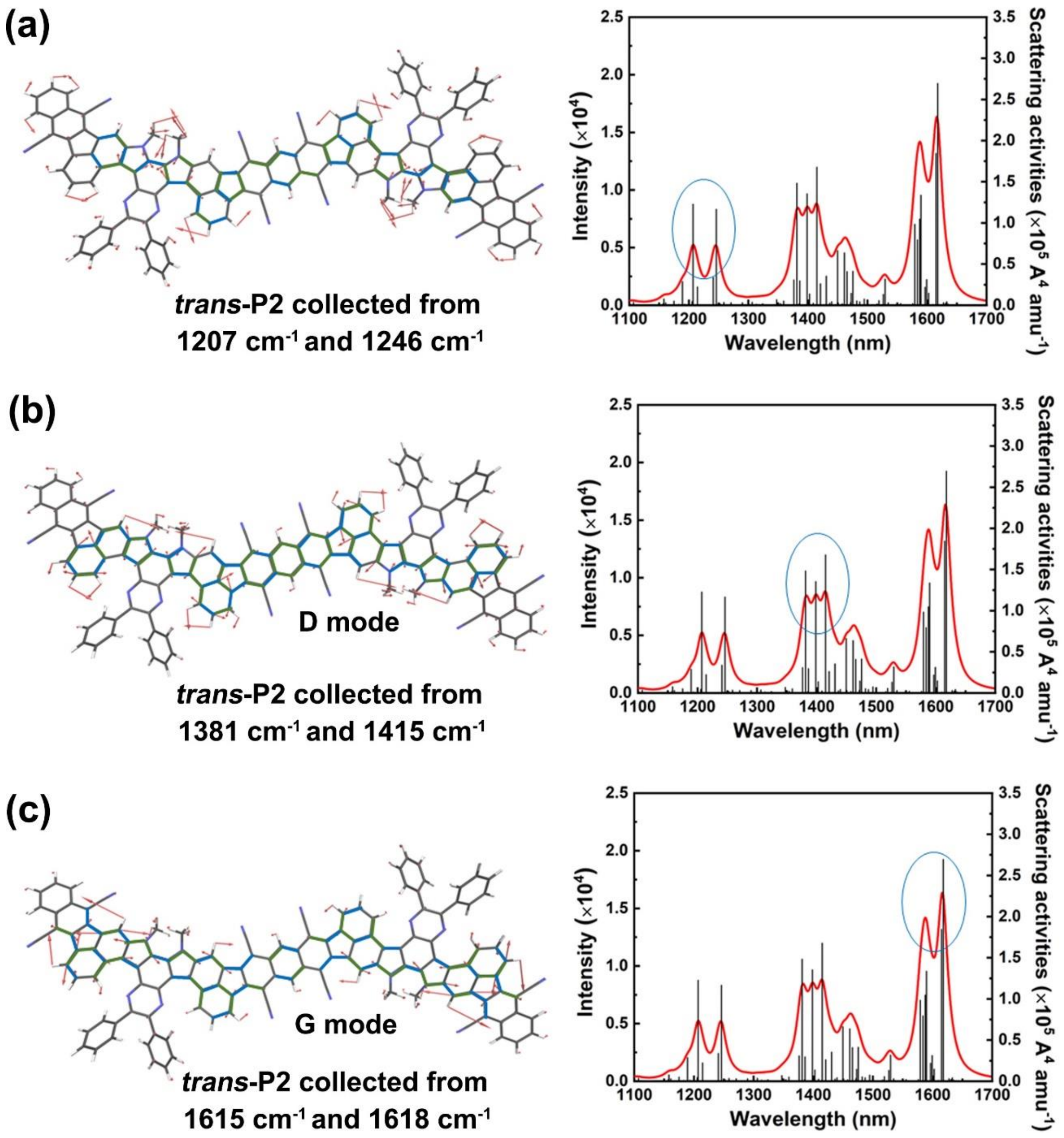

Fig. S11. The calculated normal modes and Raman spectrum of the polymer model *trans*-P2: red arrows represent displacement vectors; bonds between non-hydrogen atoms marked with different colors represent relative stretching (green) or shrinking (blue).

Evaluation Only. Created with Aspose.Pdf. Copyright 2002-2014 Aspose Pty Ltd.

S8. Photophysical and Electrochemical Properties

Table S2. Photophysical/Electrochemical Properties and DFT-Calculated Band Gaps of the Laddered model compounds and polymers

Sample	λ_{sol}^{max} [nm]	λ_{film}^{max} [nm]	$\lambda^{onset\ a}$ [nm]	λ_{PL}^{max} [nm]	$\Phi_{PL}{}^{b}$ [%]	$E_{g}^{opt\ c}$ [eV]	E_{ox}^{onset} [V]	$E_{HOMO}{}^{d}$ [eV]	E_{red}^{onset} [V]	E_{LUMO} [eV]	$E_{g}^{cv\ g}$ [eV]
N1	476, 511	–	539	533	54.0	2.30	1.22	–5.62	–	–3.32 [e]	–
N2	473, 507	–	537	528	51.0	2.31	1.21	–5.61	–	–3.30 [e]	–
P1	516, 603	525, 623	676	665	45.9	1.83	1.46	–5.88	–0.52	–3.90 [f]	1.98
P2	513, 605	519, 617	667	669	55.3	1.86	1.44	–5.86	–0.56	–3.86 [f]	2.00

[a]Absorption tail in chlorobenzene. [b]Absolute PLQYs tested in chlorobenzene. [c]Estimated from E_{g}^{opt} = 1240/λ^{onset} . [d]Estimated from E_{HOMO} = –(E_{ox}^{onset} + 4.8 – E_{Fc/Fc^+}) eV. [e]Estimated from E_{LUMO} = (E_{HOMO}+ E_{g}^{opt}) eV. [f]Estimated from E_{LUMO} = – (E_{red}^{onset} + 4.8 – E_{Fc/Fc^+}) eV. [g]Extracted from the formula of E_{g}^{cv} = –(E_{HOMO} – E_{LUMO}) eV.

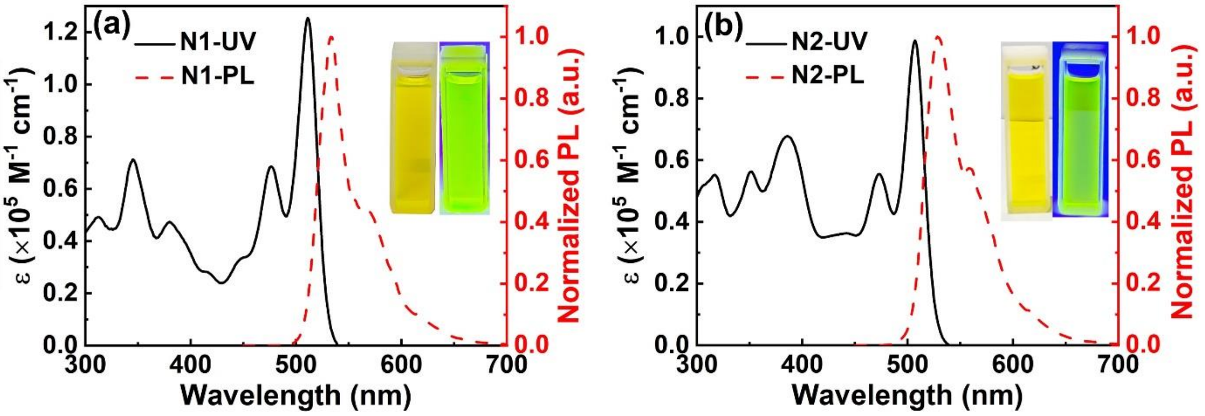

Fig. S12. Absorption spectra and normalized photoluminescence (PL) spectra of N1 and N2 in chlorobenzene solution (10^{-5} mol L^{-1}).

Evaluation Only. Created with Aspose.Pdf. Copyright 2002-2014 Aspose Pty Ltd.

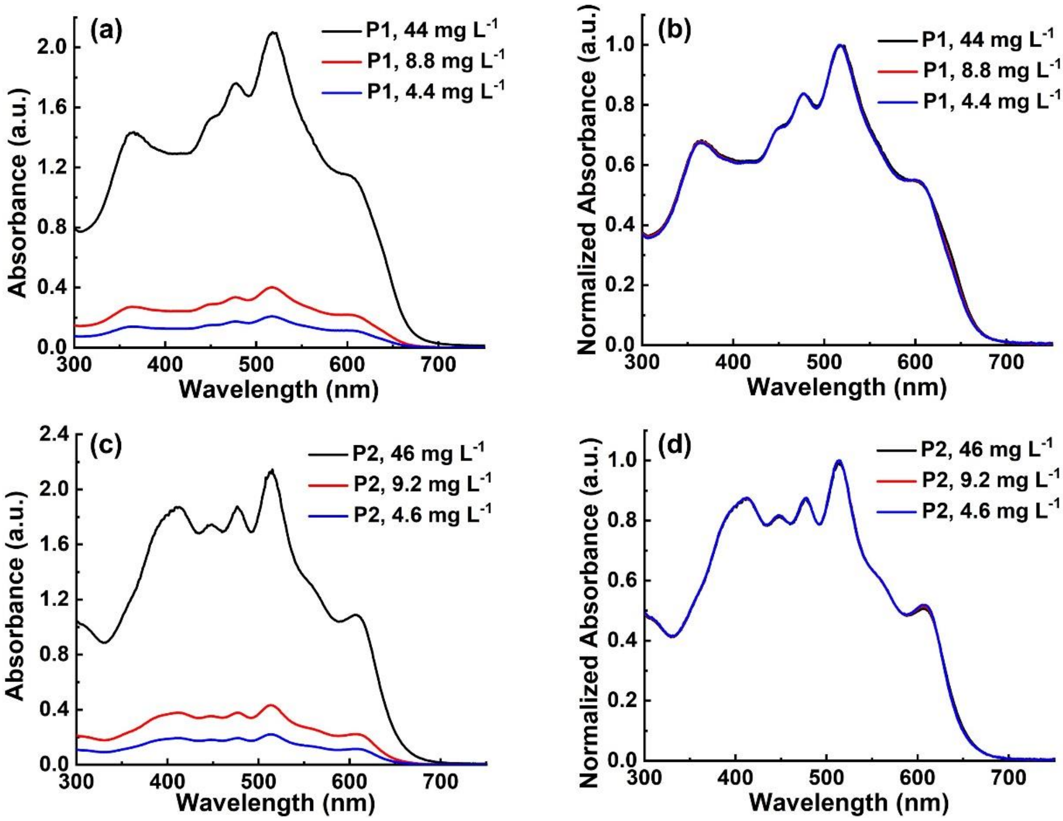

Fig. S13. The absorption spectra and normalized absorption spectra of P1 and P2 measured in chlorobenzene solution with different concentrations.

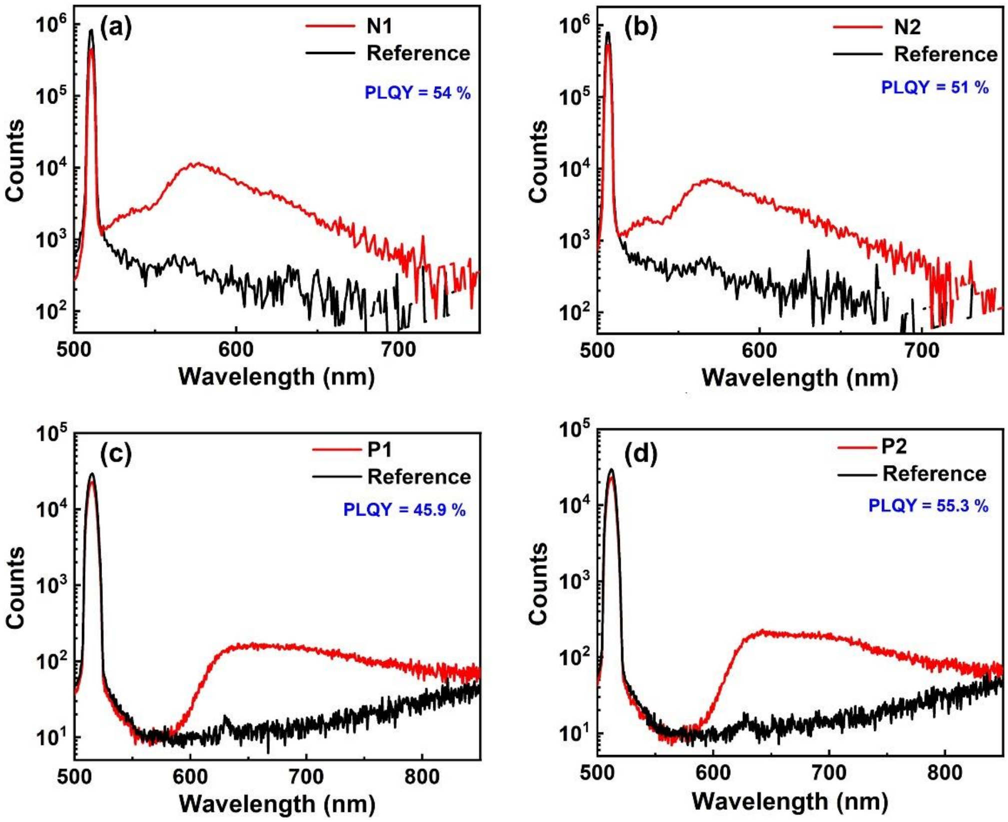

Fig. S14. The absolute PLQY profiles of N1 (a), N2 (b), P1 (c), and P2 (d) measured in chlorobenzene.

S9. Quantum Chemical Calculations

Quantum chemical studies were performed using density functional theory (DFT) implemented in GAUSSIAN 16 package. All ground state

Evaluation Only. Created with Aspose.Pdf. Copyright 2002-2014 Aspose Pty Ltd.

optimized geometries were obtained using the B3LYP functional together with the def2-SVP basis set [6-7]. The dispersion correction was conducted by Grimme's D3 version[8], while the long alkyl chain was simplified to be methyl group. On the basis of the optimized ground state geometries, the vertical excitation energies were evaluated at PBE0/def2-TZVP by TDDFT treatment[9], while the simulation of Raman spectra was carried out at the same level by DFT treatment. The solvent effect of chlorobenzene (ε = 5.70) was taken into account by a polarizable continuum model (PCM) [8]. To reduce the computation costs, the UV-Vis spectrum was calculated at TDDFT//PBE0/def2-SVP level. Excited states analysis was processed with the TDDFT results using Multiwfn 3.8 program according to the program manual and literature method[10-11].

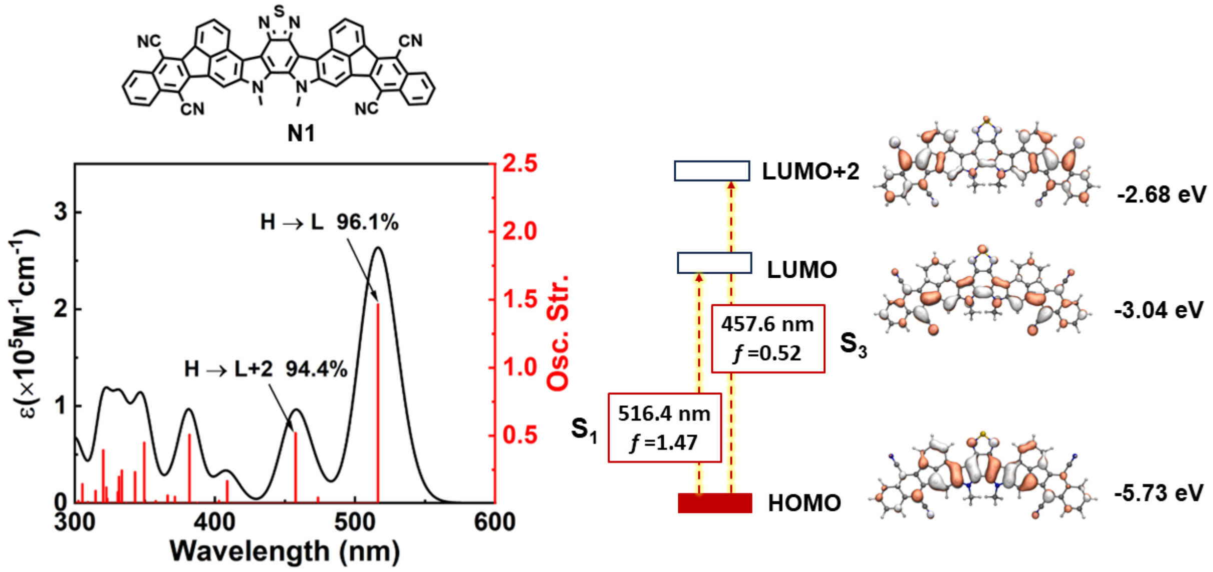

Fig. S15. Absorption spectra and typical electron transitions of N1 evaluated at PBE0/def2-SVP by TDDFT treatment in chlorobenzene solution.

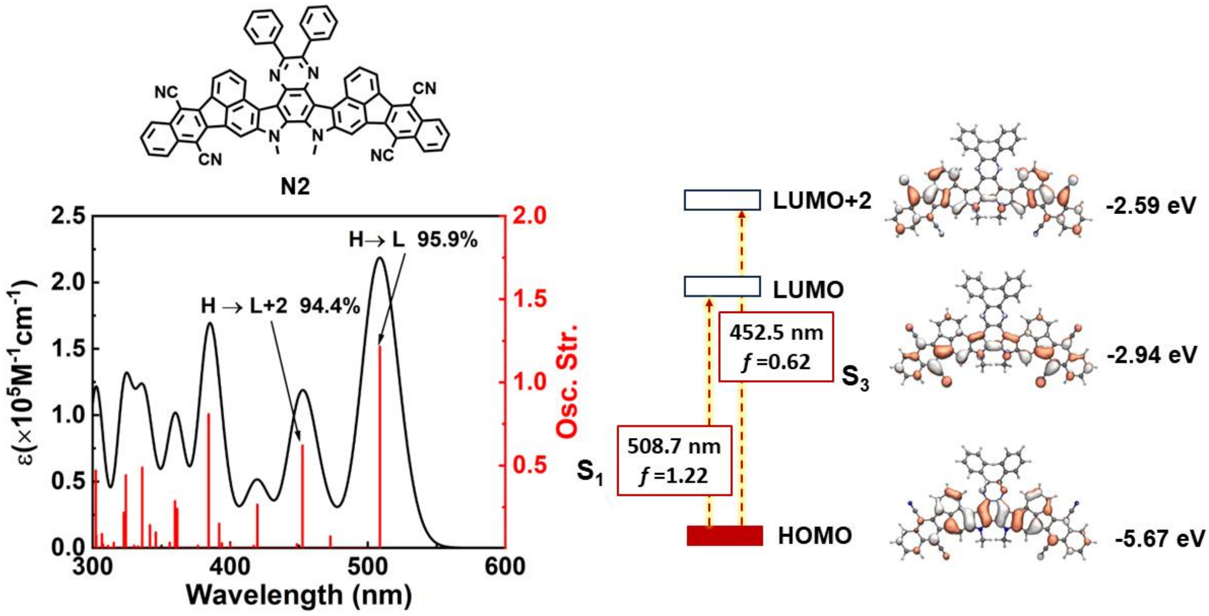

Fig. S16. Absorption spectra and typical electron transitions of N2 evaluated at PBE0/def2-SVP by TDDFT treatment in chlorobenzene solution.

Evaluation Only. Created with Aspose.Pdf. Copyright 2002-2014 Aspose Pty Ltd.

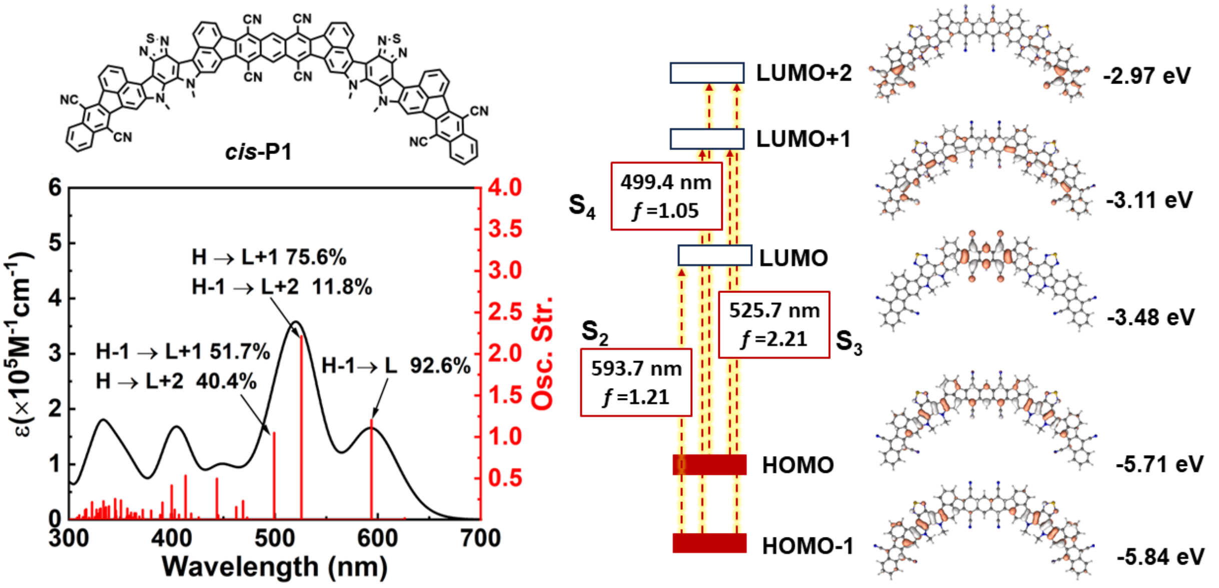

Fig. S17. Absorption spectra and typical electron transitions of the polymer model *cis*-P1 evaluated at PBE0/def2-SVP by TDDFT treatment in chlorobenzene solution.

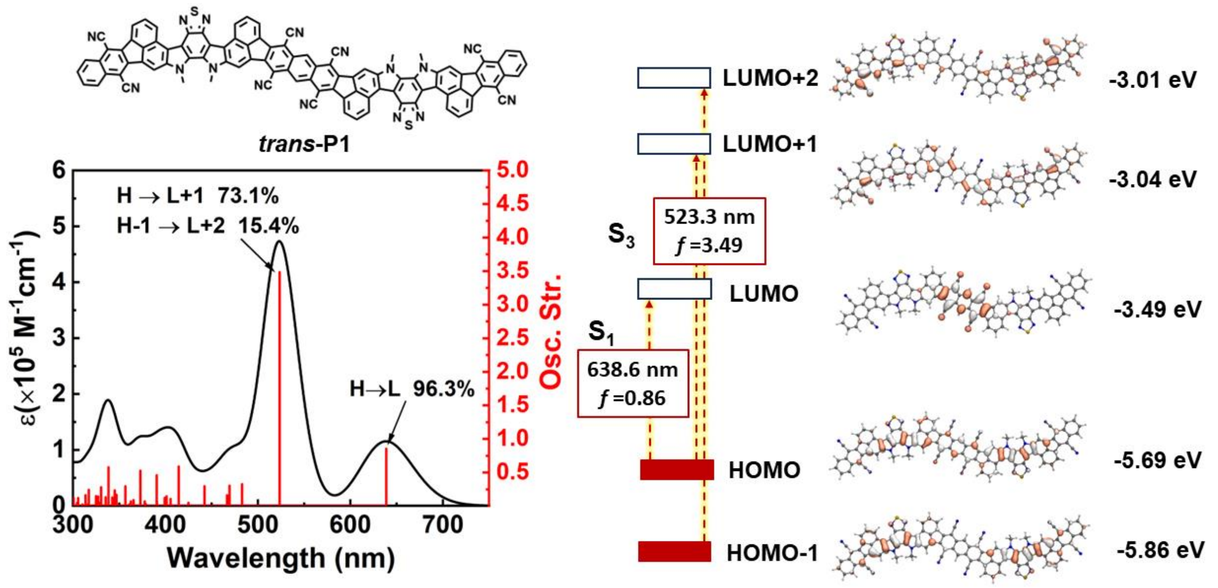

Fig. S18. Absorption spectra and typical electron transitions of the polymer model *trans*-P1 evaluated at PBE0/def2-SVP by TDDFT treatment in chlorobenzene solution.

Evaluation Only. Created with Aspose.Pdf. Copyright 2002-2014 Aspose Pty Ltd.

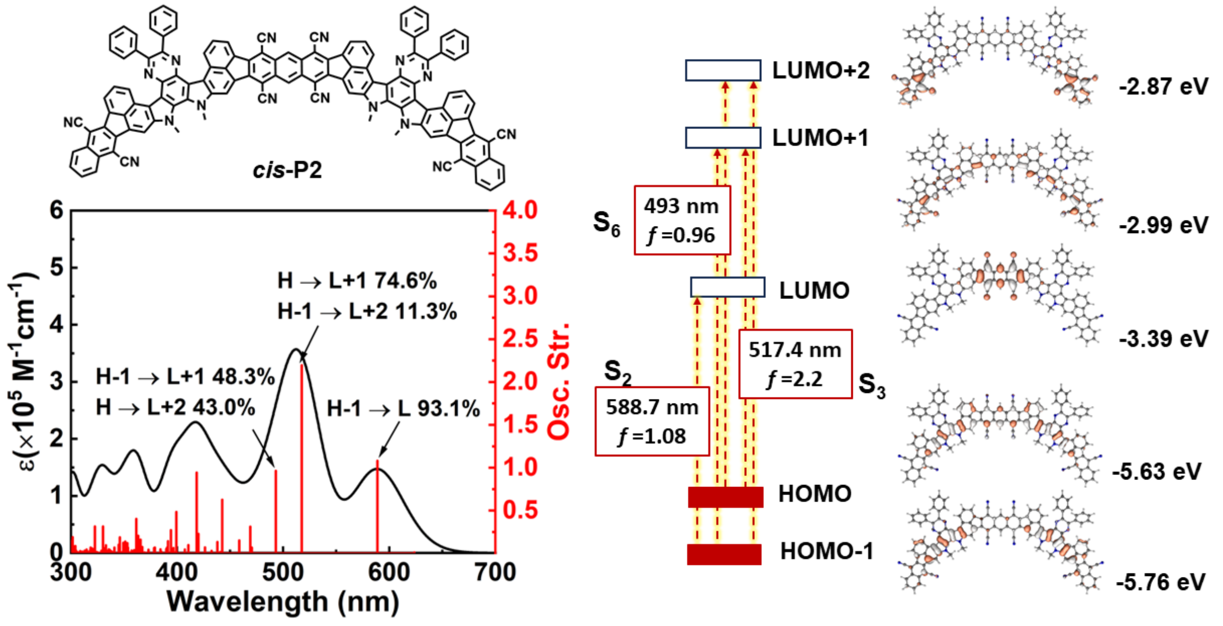

Fig. S19. Absorption spectra and typical electron transitions of the polymer model *cis*-P2 evaluated at PBE0/def2-SVP by TDDFT treatment in chlorobenzene solution.

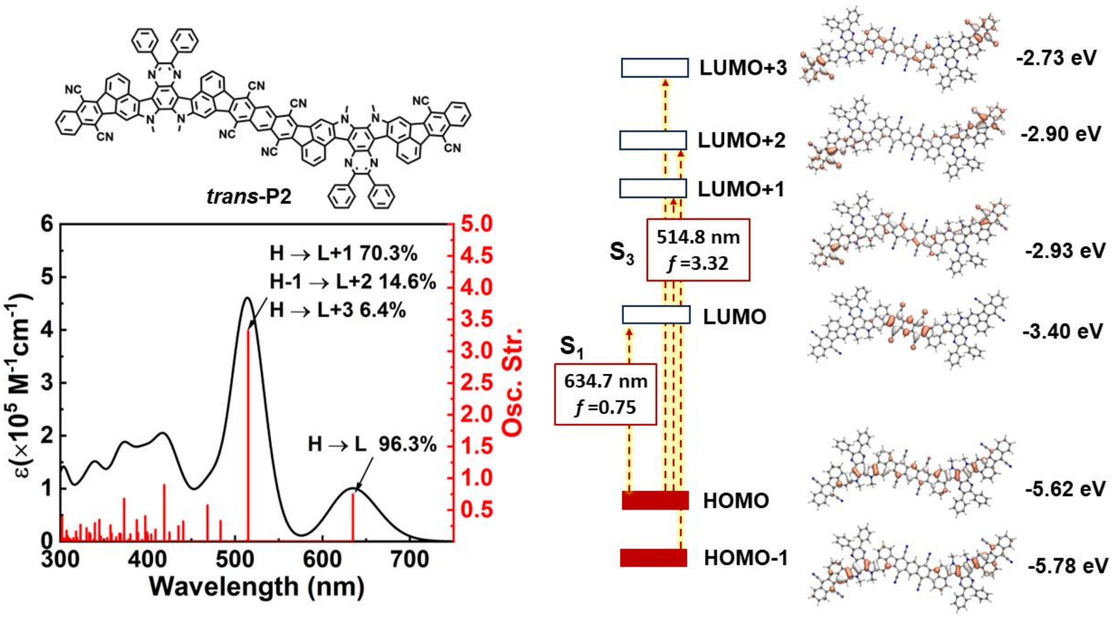

Fig. S20. Absorption spectra and typical electron transitions of the polymer model *trans*-P2 evaluated at PBE0/def2-SVP by TDDFT treatment in chlorobenzene solution.

Evaluation Only. Created with Aspose.Pdf. Copyright 2002-2014 Aspose Pty Ltd.

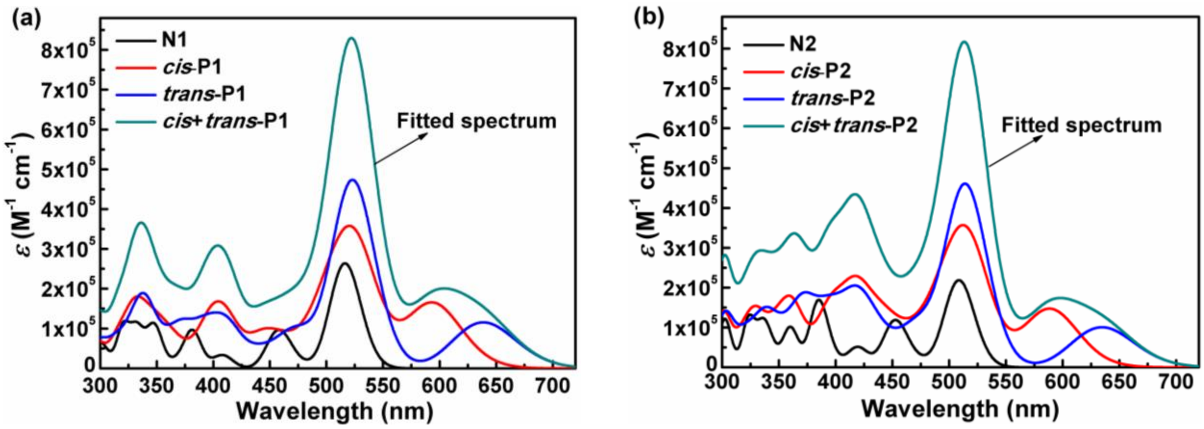

Fig. S21. Absorption spectra of N1, N2, and the polymer models (*trans*-P1, *cis*-P1, *trans*-P2, and *cis*-P2) calculated at PBE0/def2-SVP by TDDFT treatment in chlorobenzene solution.

S10. GIWAXS Data of Polymer Films

Table S3. Summary of stacking characteristics of P1 and P2 thin films.

polymer	d-d Stacking in the direction of in-plane				π-π Stacking in the direction of out-of-plane			
	Peak (Å-1)	Distance (Å)	FWHM (Å-1)	CCL (Å)	Peak (Å-1)	Distance (Å)	FWHM (Å-1)	CCL (Å)
P1	0.23	27.31	0.2114	26.7496	1.69	3.72	0.3752	15.0716
P2	0.25	25.13	0.2709	20.8743	1.62	3.88	0.3978	14.2153

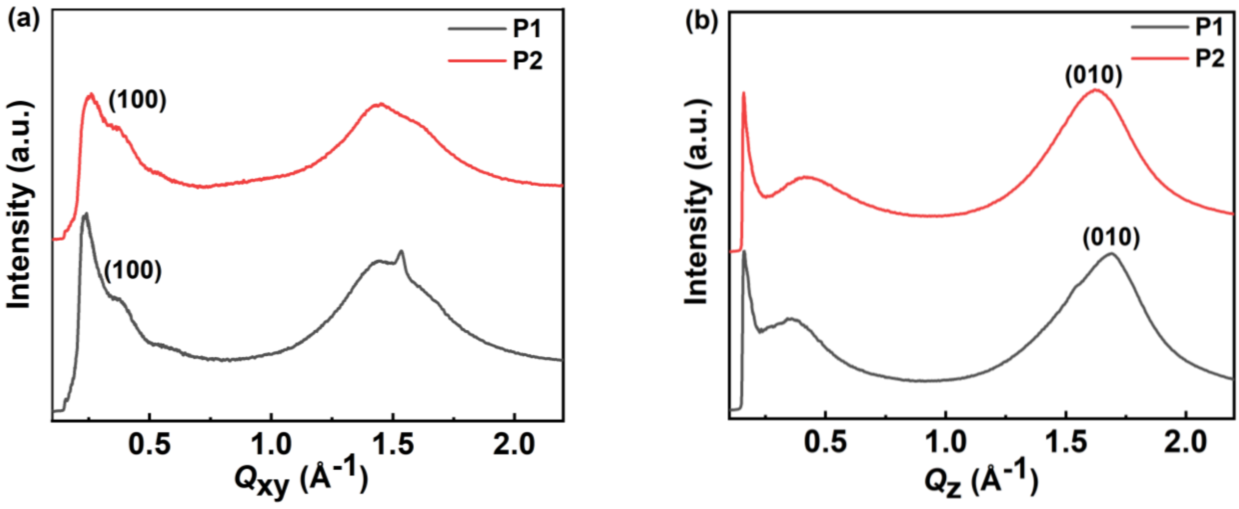

Fig. S22. 1D-GIWAXS curves of P1 and P2 films. (a) in plane. (b) out-of-plane.

Evaluation Only. Created with Aspose.Pdf. Copyright 2002-2014 Aspose Pty Ltd.

S11. Transistor Devices Fabrication and Measurement

The organic field-effect transistors (OFET) with bottom-gate bottom-contact device architecture was fabricated to study the charge-carrier transport properties of P1 and P2. A silicon wafer with a layer of silica oxide (285 nm) and pre-deposited interdigitated gold electrodes was used as the substrate, among which the silicon, silicon dioxide, and gold electrodes serve as gate electrode, dielectric layer and source/drain electrodes, respectively. The wafer was firstly immersed into piranha solution at 60 °C for 30 min, followed by ultrasonic cleaning in ethanol for 10 min. Subsequently, the substrates were subjected to a vapor phase modification of octadecyltrichlorosilane (OTS) at 120 °C for 3 h. After that, the OTS-modified substrates were ultrasonically cleaned with *n*-hexane, ethanol, and chloroform for 10 minutes, respectively; and then blown-dried by nitrogen gun. Subsequent preparation and measurement of the OFET devices were performed at nitrogen atmosphere. The solution of P1 and P2 with a concentration of 5 mg mL^{-1} was prepared by using *o*-dichlorobenzene as solvent. Semiconductor films were deposited on the surface of OTS-modified substrates via a spin-coating process at 1500 rpm for 60 s. Before measuring, the devices were subjected to the thermal treatment at 150 °C for 30 min.

The transfer and output characteristic curves of OFET devices based P1 and P2 were measured by using Primarius FS-Pro semiconductor parameter analyzer under nitrogen atmosphere. The mobilities were extracted from saturation area and calculated according to the equation:

$$I_D = (W\mu C_i/2L)(V_G - V_{TH})^2$$

where μ is mobility; I_D is current between source and drain electrodes; V_G is gate voltage; V_{TH} is the threshold voltage; W and L are the channel width and length of OFET device, respectively. C_i is the capacitance per unit area of dielectric and the C_i value of silicon dioxide layer in this work is 11.5 nF·cm^{-2}.

Table S4. Summary of device performance of P1- and P2-based OFETs.

Materials	Maximum/Average Mobilities (cm^2 V^{-1} s^{-1})		V_T (V)		I_{on}/I_{off}	
	Hole	Electron	Hole	Electron	Hole	Electron
P1	0.008/ 0.003	0.009/ 0.004	−36 ± 6	23 ± 10	10^2~10^3	10^3~10^4
P2	0.01/ 0.007	0.01/ 0.005	−34 ± 5	26 ± 6	10^3~10^4	10^3~10^4

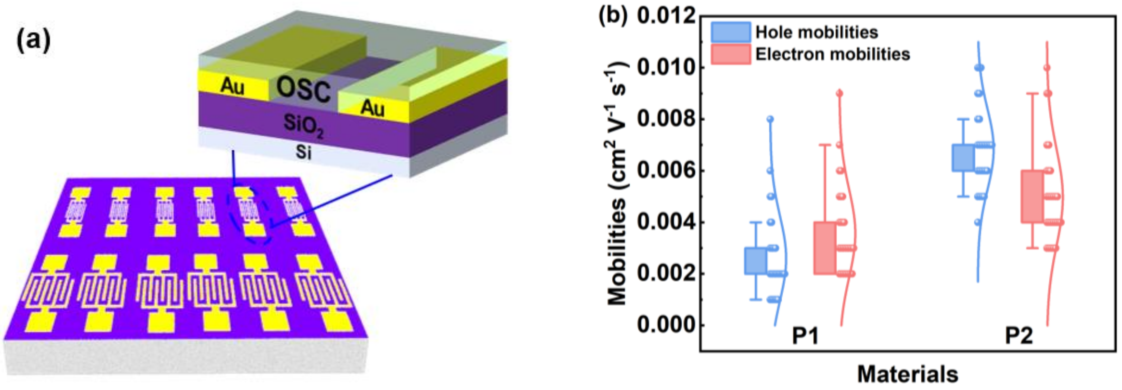

Fig. S23. (a) Schematic diagram of BGBC type OFETs. (b) Statistical distribution diagram of hole and electron mobilities of P1 and P2 with 30 devices, respectively. The box chart ranges within 1.5 quartile.

Evaluation Only. Created with Aspose.Pdf. Copyright 2002-2014 Aspose Pty Ltd.

Table S5. Comparison of charge-transport performance between the reported cLPs and as-prepared cLPs (P1 and P2) characterized by OFETs.

Materials	Hole Mobility ($cm^2 V^{-1} s^{-1}$)	Electron Mobility ($cm^2 V^{-1} s^{-1}$)	Processing solvent	Ref.
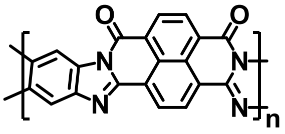	–	0.1	MSA	13
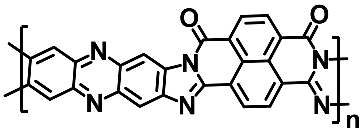	–	1.2×10^{-4}	MSA	14
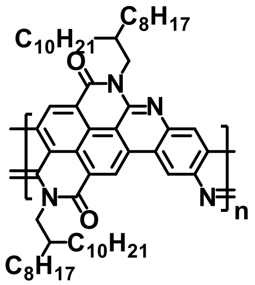	–	2.6×10^{-3}	CB	15
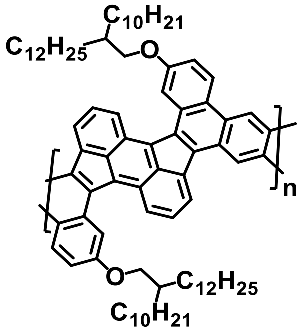	2.0×10^{-5}	–	CB	16
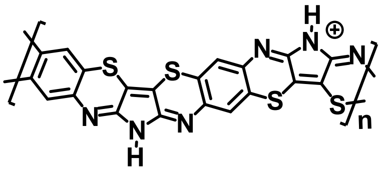	3.1×10^{-3}	–	MSA	17
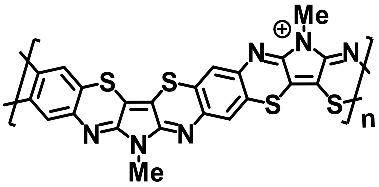	1.3×10^{-3}	–	MSA	17
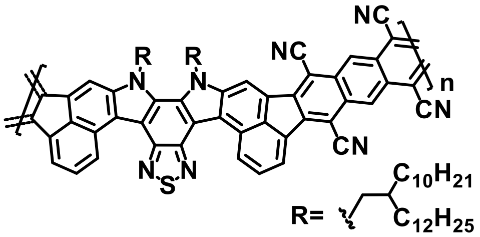	**8×10^{-3}**	**9×10^{-3}**	**CB**	**This work**

Evaluation Only. Created with Aspose.Pdf. Copyright 2002-2014 Aspose Pty Ltd.

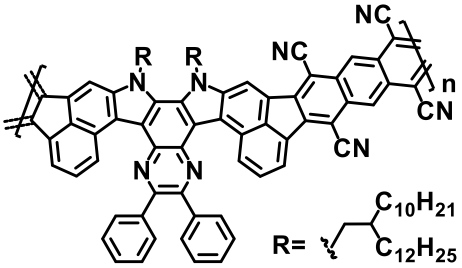	**0.01**	**0.01**	**CB**	**This work**

Processing solvents of OFET devices. MSA: Methanesulfonic acid; CB: Chlorobenzene.

Evaluation Only. Created with Aspose.Pdf. Copyright 2002-2014 Aspose Pty Ltd.

S12. ^{1}H and ^{13}C NMR of Compounds

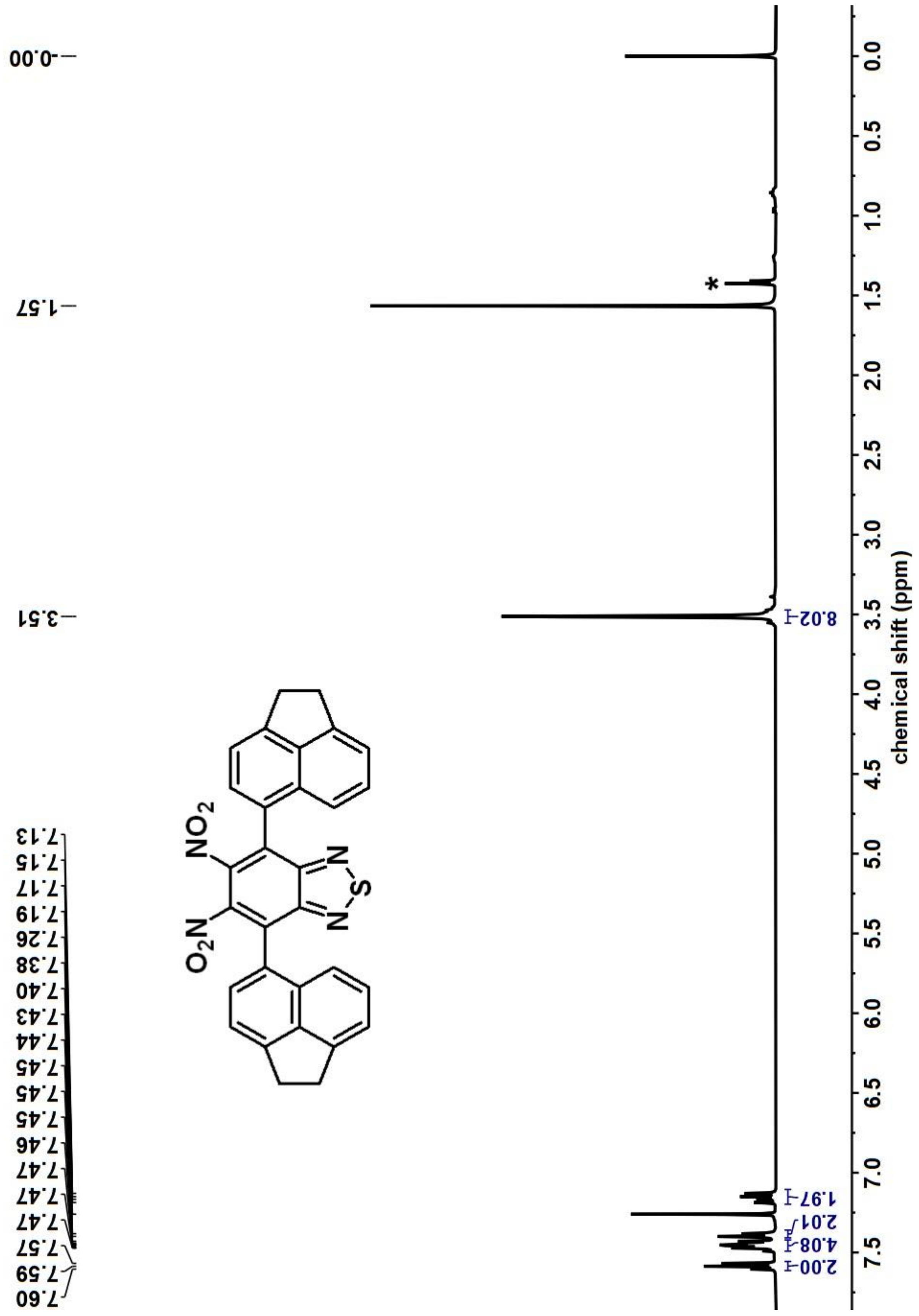

Fig. S24. ^{1}HNMR spectra of compound 3 in $CDCl_3$ (298 K).

Evaluation Only. Created with Aspose.Pdf. Copyright 2002-2014 Aspose Pty Ltd.

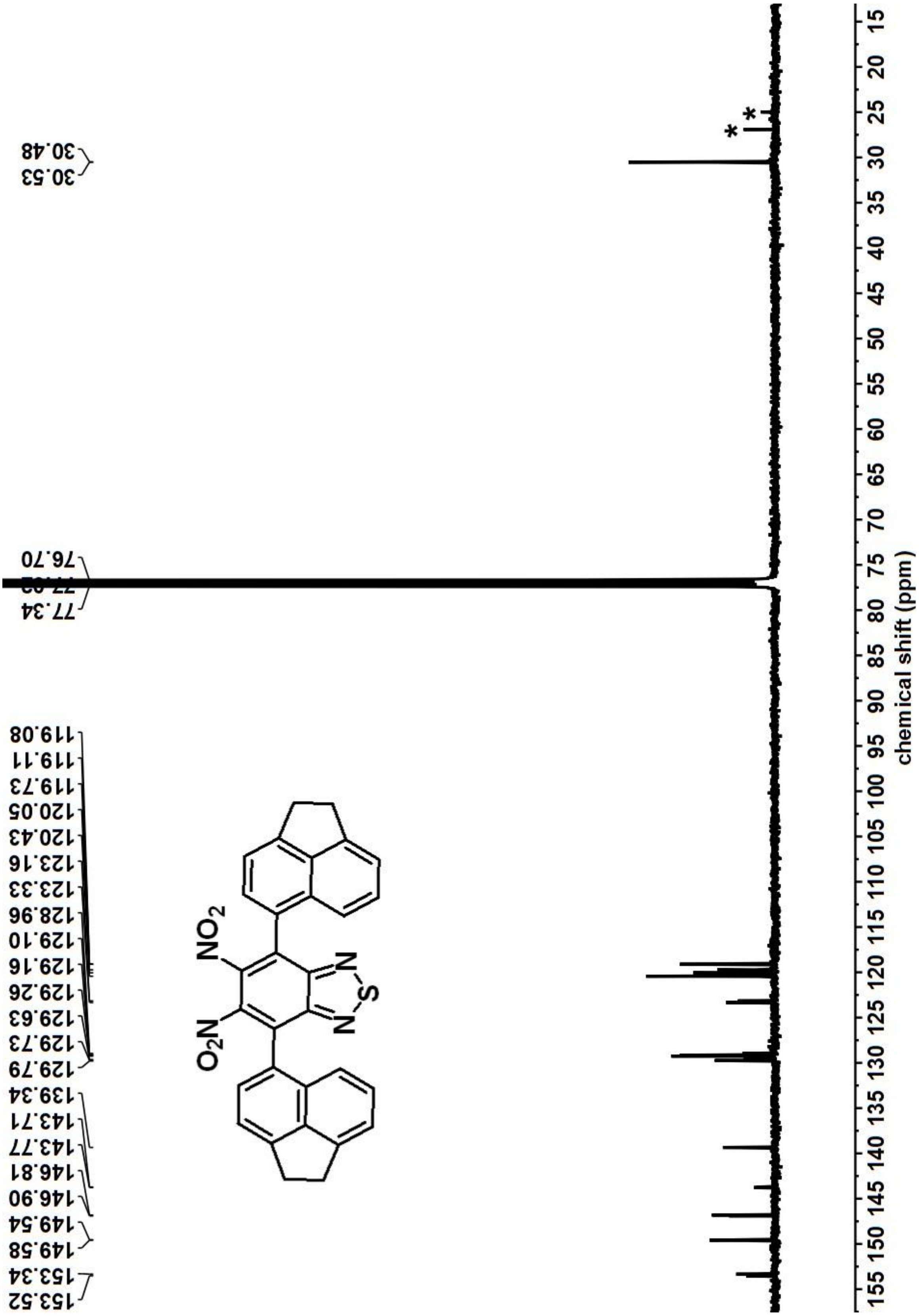

Fig. S25. ^{13}CNMR spectra of compound 3 in $CDCl_3$ (298 K).

Evaluation Only. Created with Aspose.Pdf. Copyright 2002-2014 Aspose Pty Ltd.

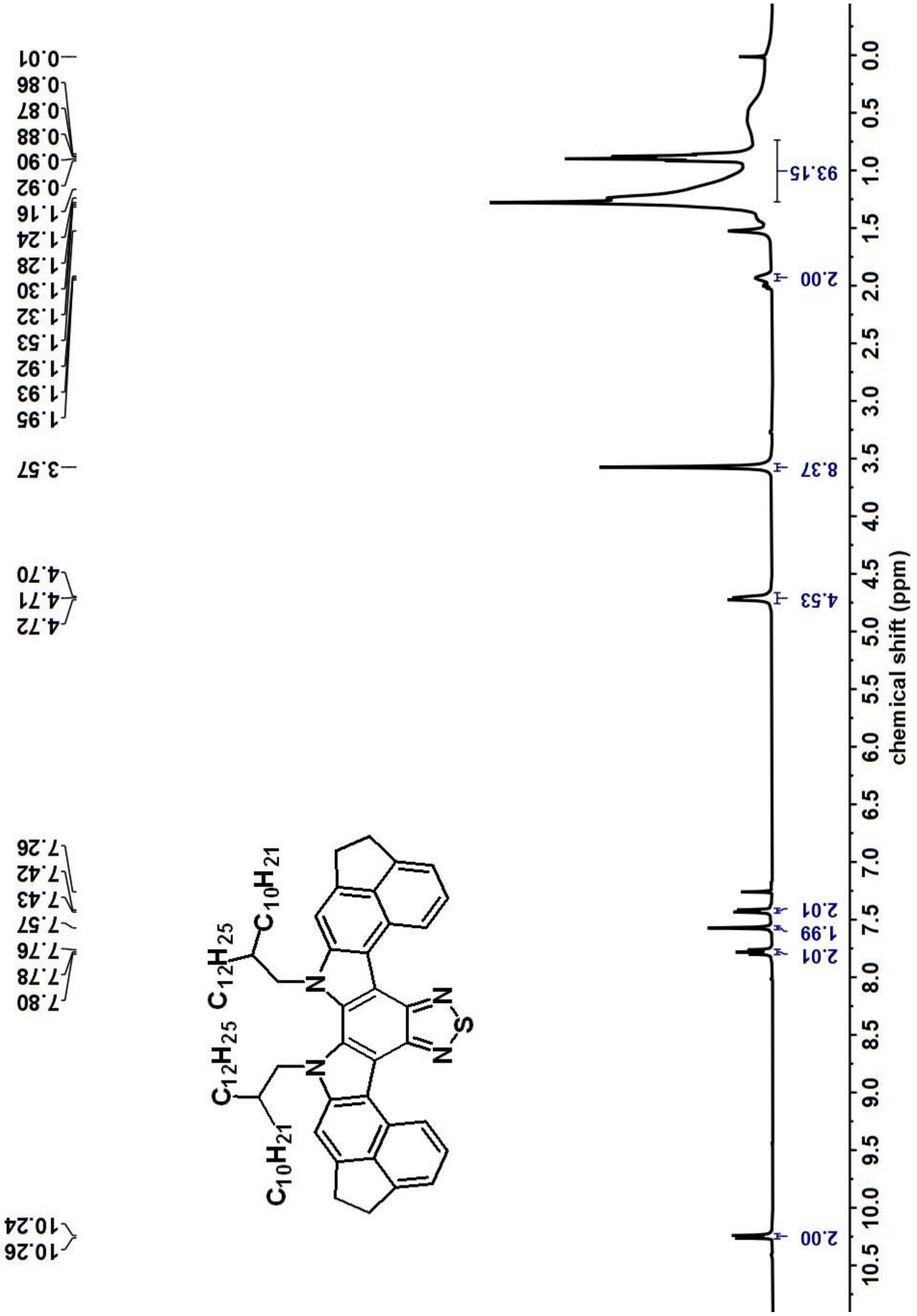

Fig. S26. ^{1}HNMR spectra of compound 4 in $CDCl_3$ (313 K).

Evaluation Only. Created with Aspose.Pdf. Copyright 2002-2014 Aspose Pty Ltd.

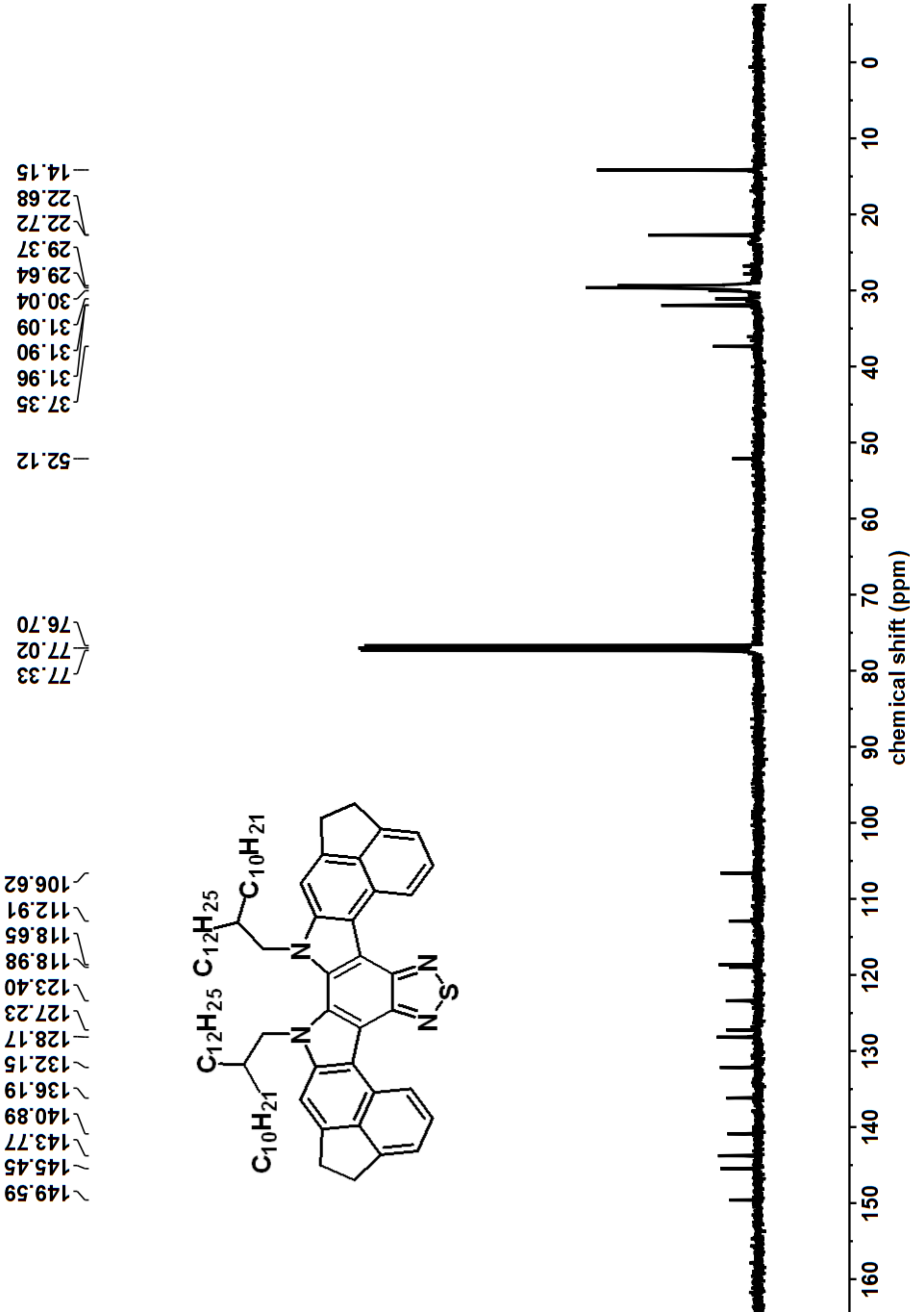

Fig. S27. ^{13}CNMR spectra of compound 4 in $CDCl_3$ (298 K).

Evaluation Only. Created with Aspose.Pdf. Copyright 2002-2014 Aspose Pty Ltd.

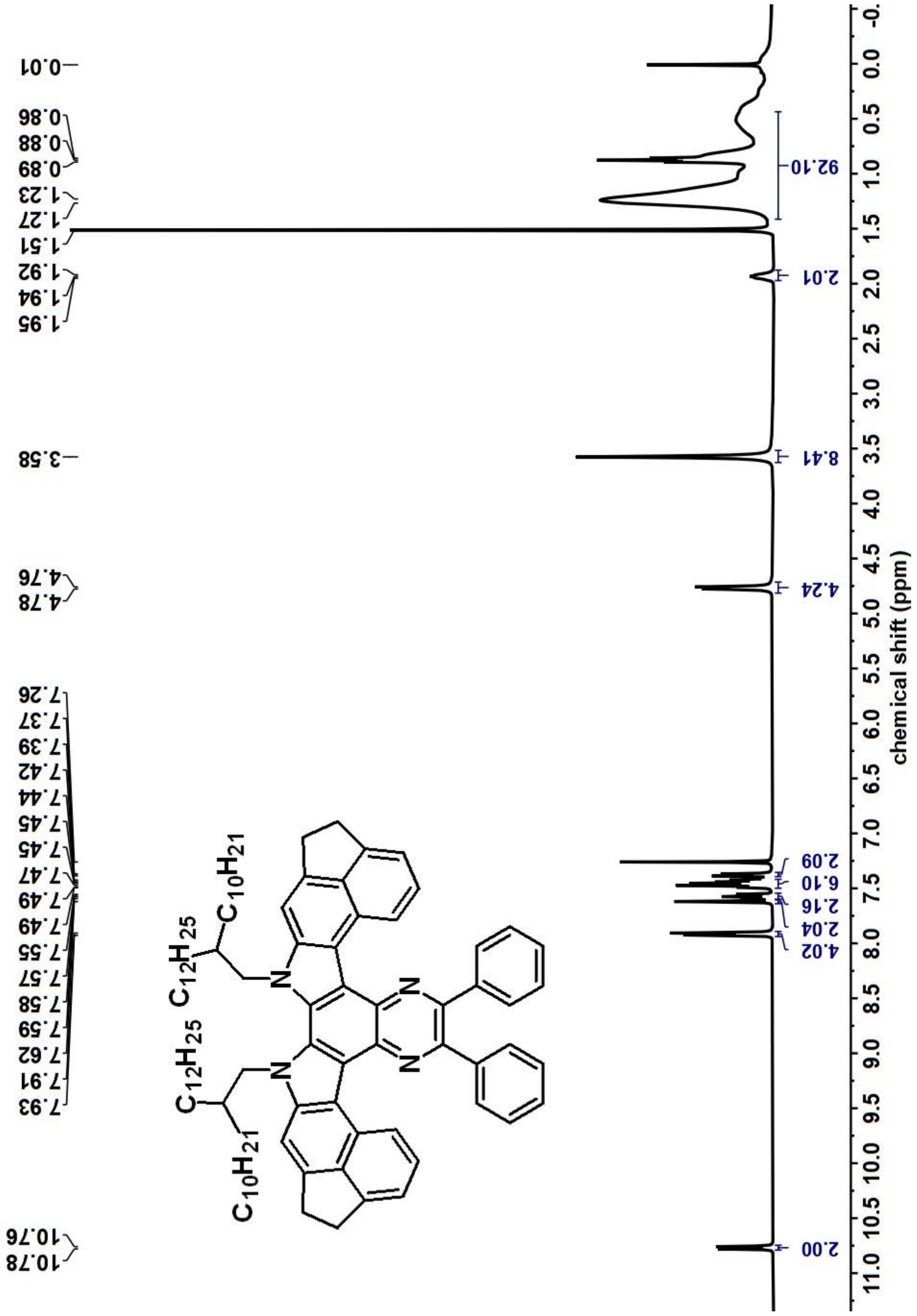

Fig. S28. ^{1}HNMR spectra of compound 6 in $CDCl_3$ (313 K).

Evaluation Only. Created with Aspose.Pdf. Copyright 2002-2014 Aspose Pty Ltd.

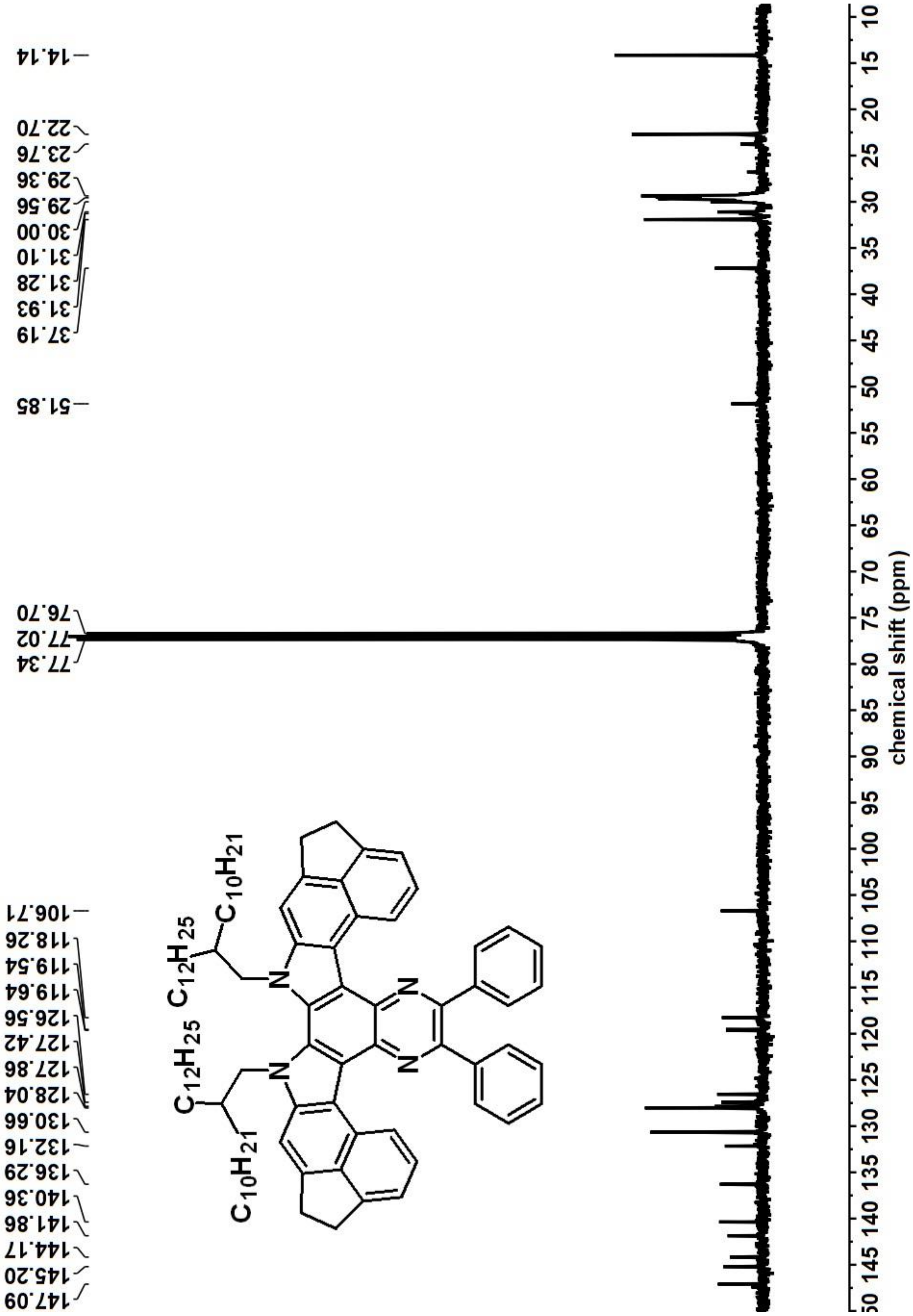

Fig. S29. ^{13}CNMR spectra of compound 6 in $CDCl_3$ (298 K).

Evaluation Only. Created with Aspose.Pdf. Copyright 2002-2014 Aspose Pty Ltd.

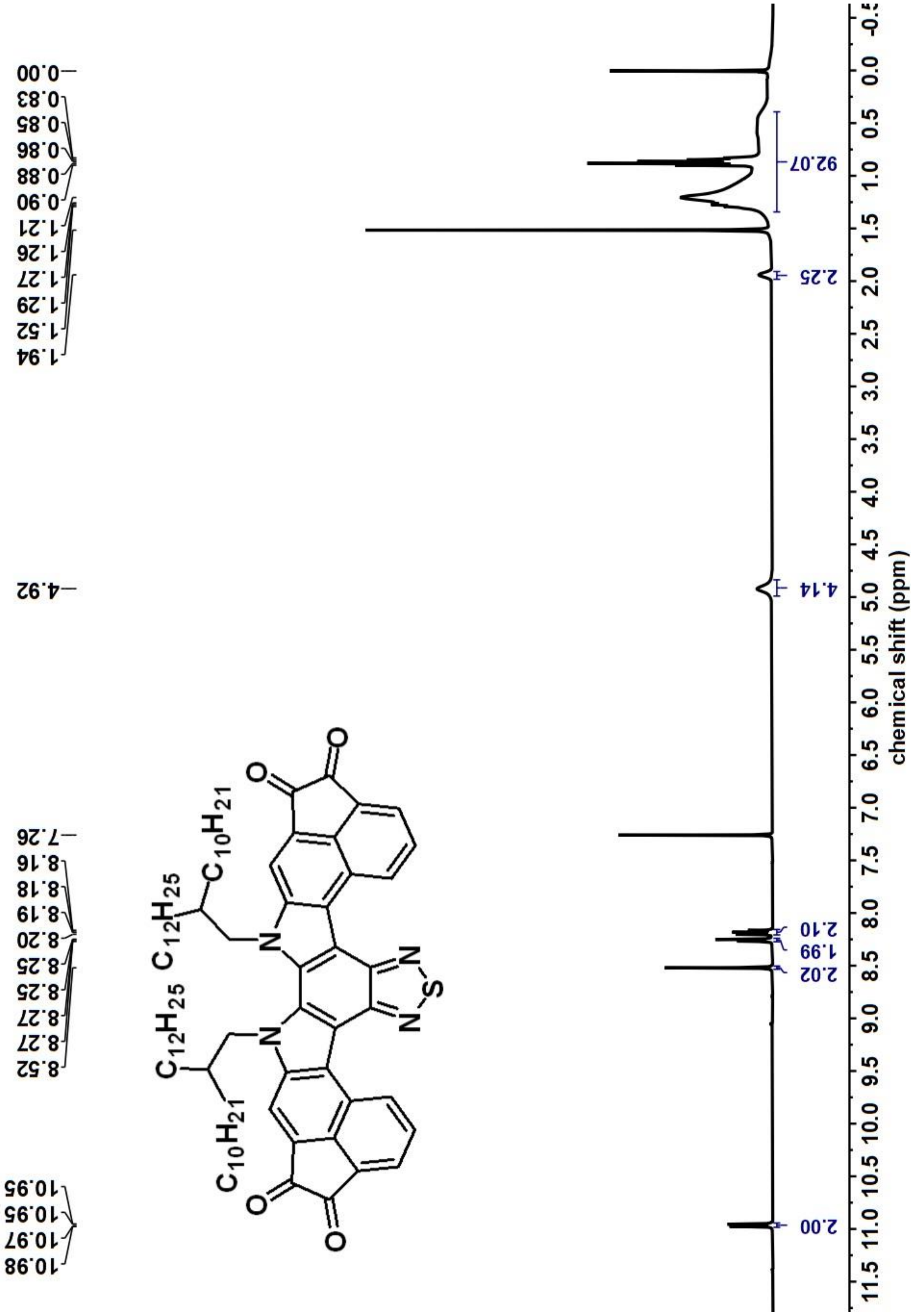

Fig. S30. ^{1}HNMR spectra of compound M1 in $CDCl_3$ (313 K).

Evaluation Only. Created with Aspose.Pdf. Copyright 2002-2014 Aspose Pty Ltd.

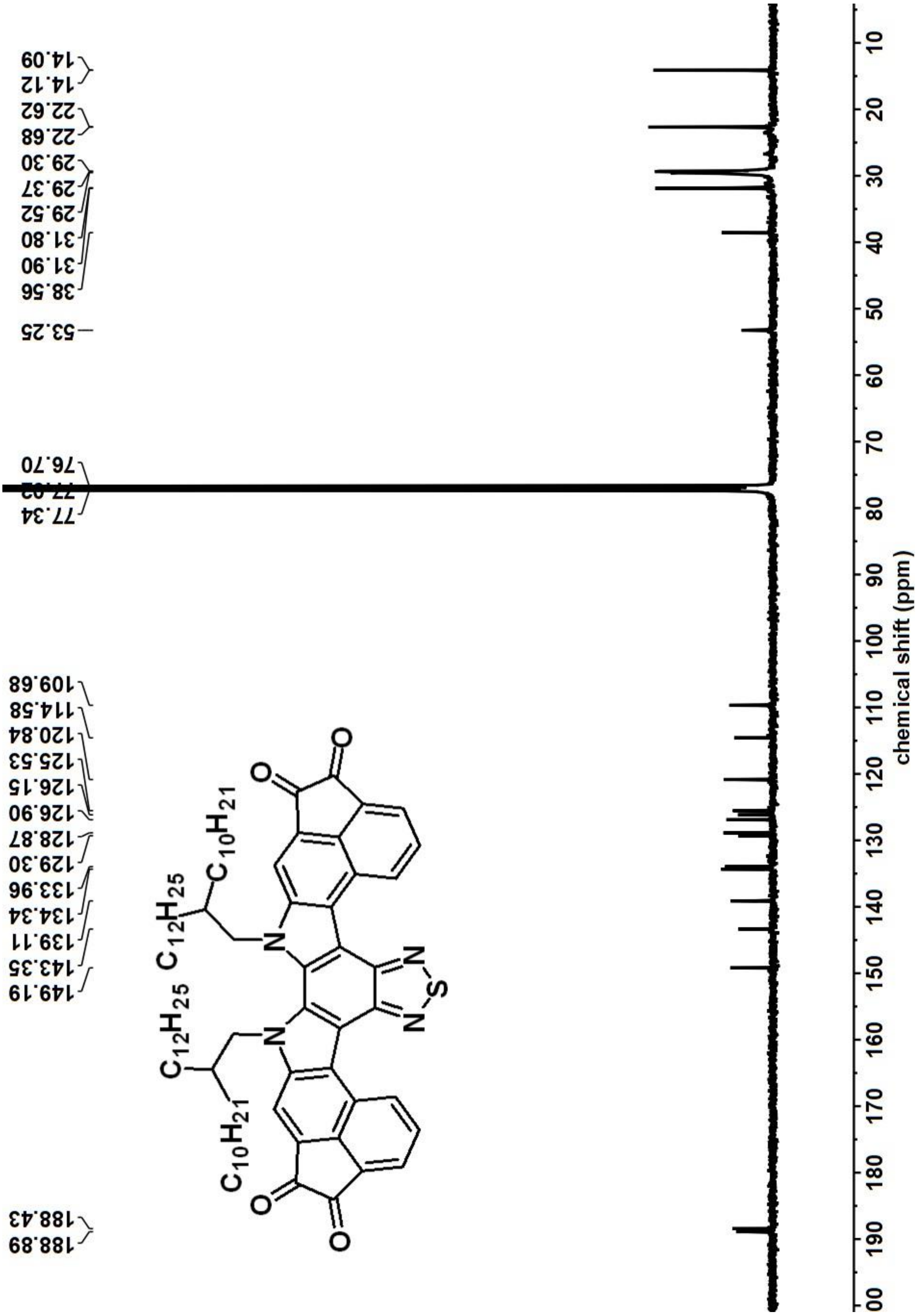

Fig. S31. ^{13}CNMR spectra of compound M1 in $CDCl_3$ (298 K).

Evaluation Only. Created with Aspose.Pdf. Copyright 2002-2014 Aspose Pty Ltd.

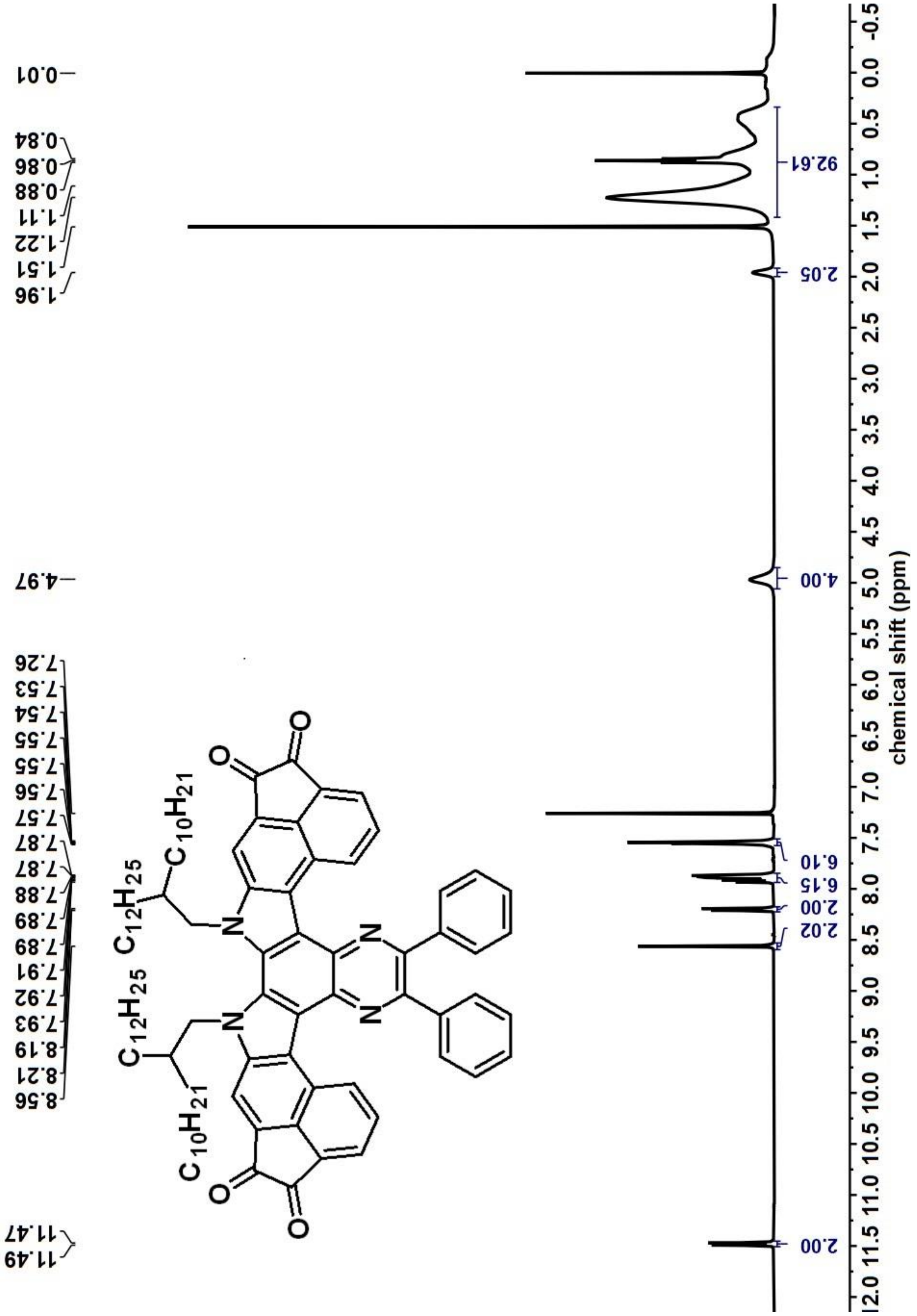

Fig. S32. ^{1}HNMR spectra of compound M2 in $CDCl_3$ (313 K).

Evaluation Only. Created with Aspose.Pdf. Copyright 2002-2014 Aspose Pty Ltd.

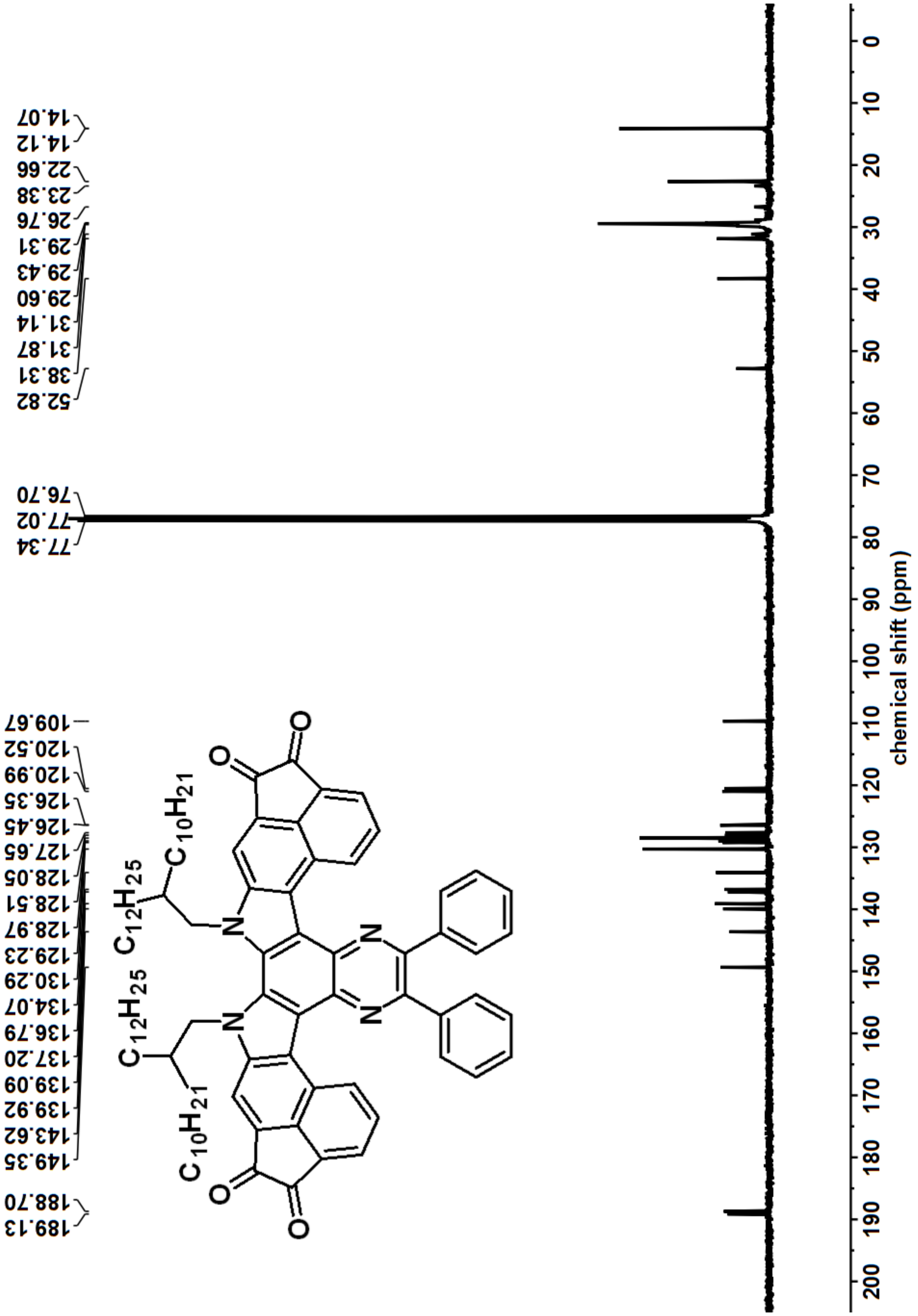

Fig. S33. ^{13}CNMR spectra of compound M2 in $CDCl_3$ (298 K)

Evaluation Only. Created with Aspose.Pdf. Copyright 2002-2014 Aspose Pty Ltd.

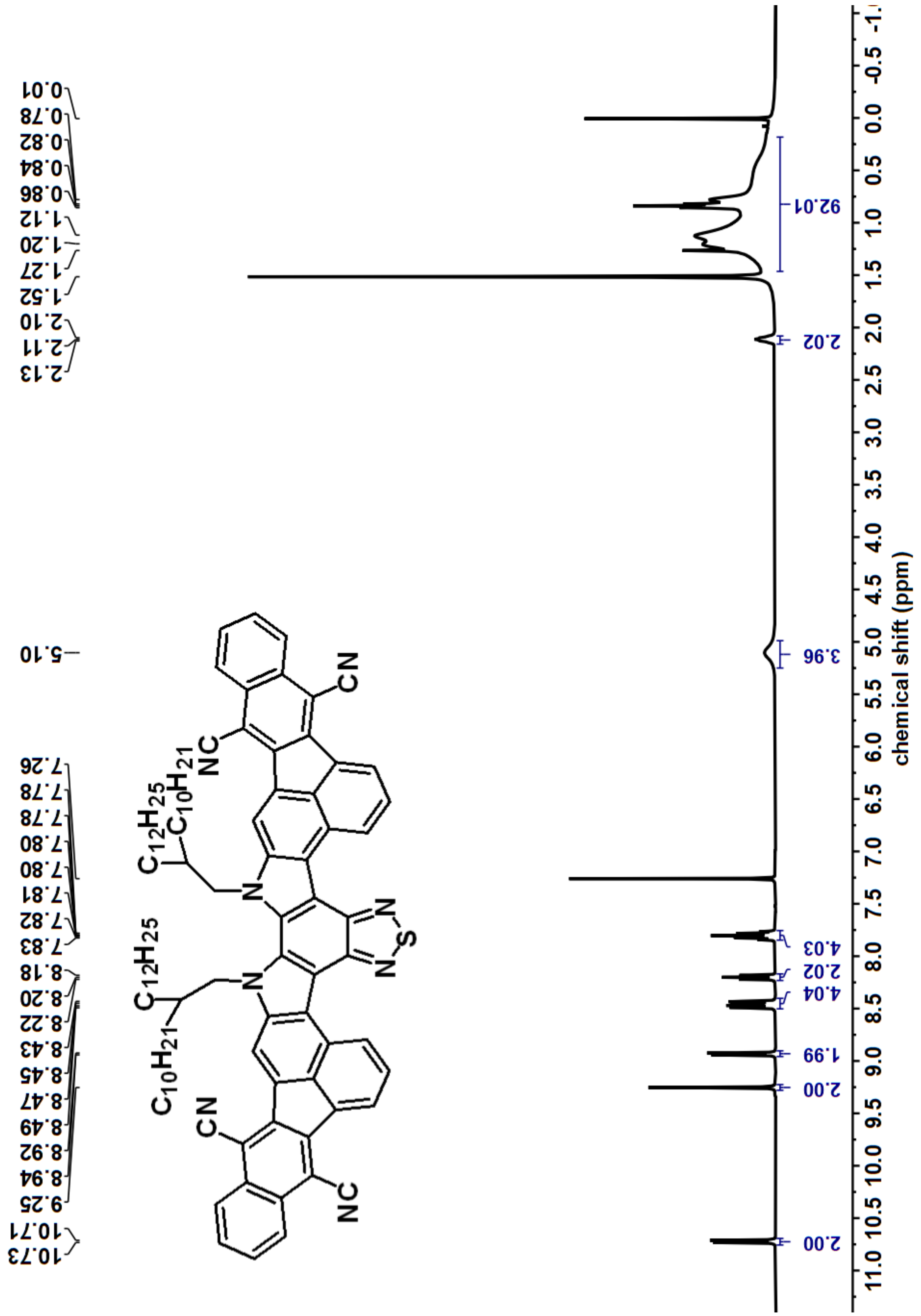

Fig. S34. ^{1}HNMR spectra of compound N1 in $CDCl_3$ (313 K).

Evaluation Only. Created with Aspose.Pdf. Copyright 2002-2014 Aspose Pty Ltd.

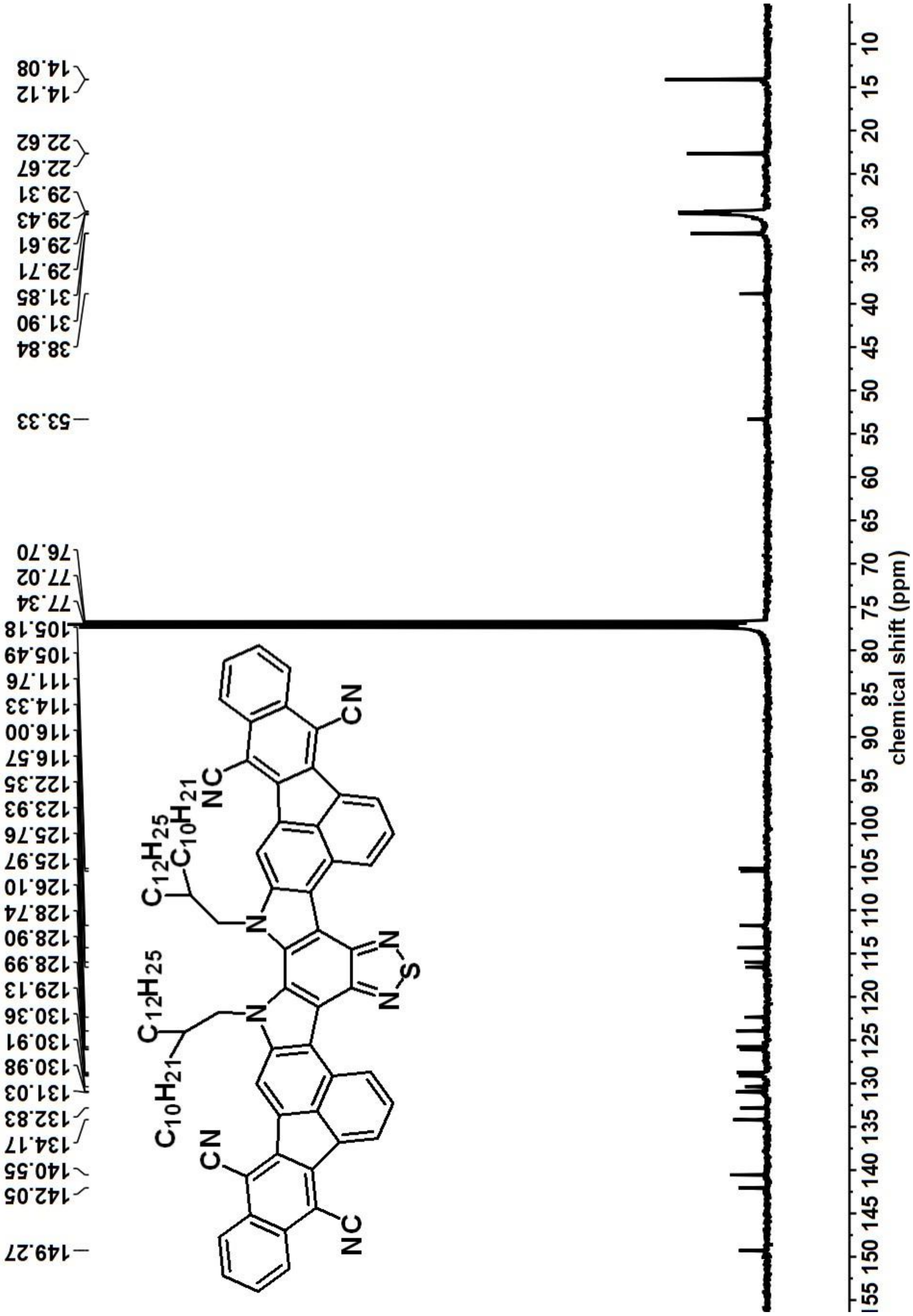

Fig. S35. ^{13}CNMR spectra of compound N1 in $CDCl_3$ (298 K).

Evaluation Only. Created with Aspose.Pdf. Copyright 2002-2014 Aspose Pty Ltd.

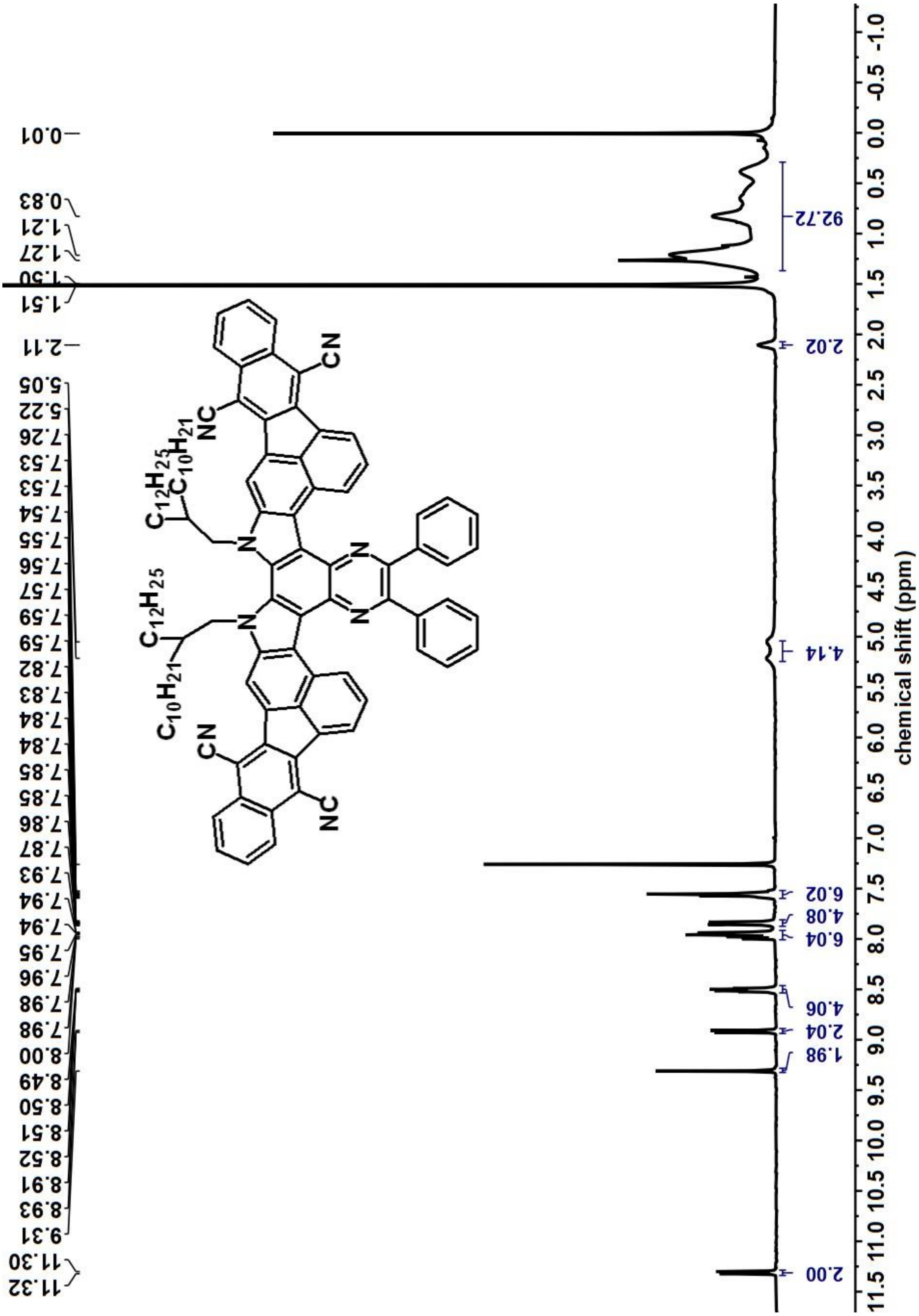

Fig. S36. ^{1}HNMR spectra of compound N2 in $CDCl_3$ (313 K).

Evaluation Only. Created with Aspose.Pdf. Copyright 2002-2014 Aspose Pty Ltd.

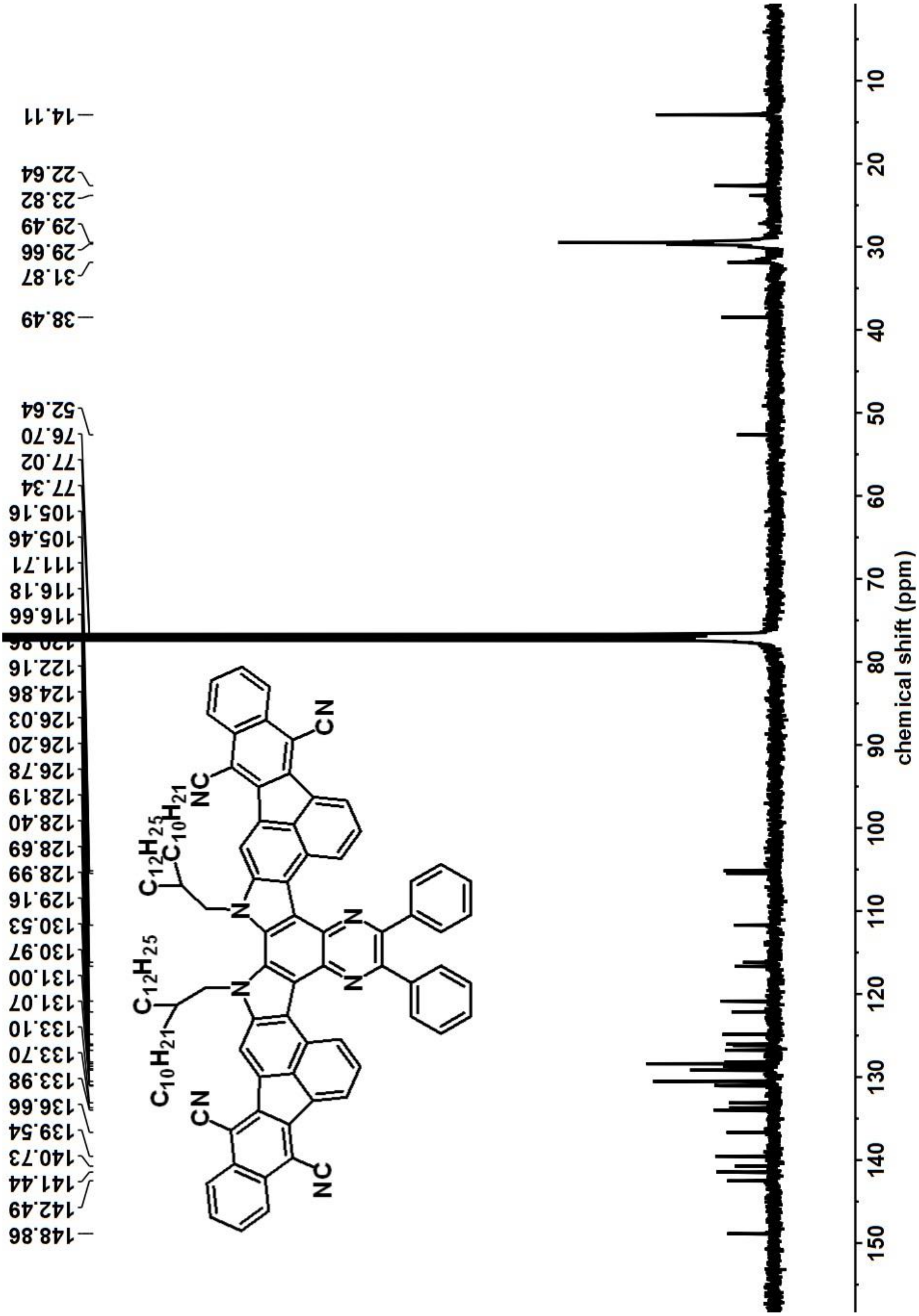

Fig. S37. ^{13}CNMR spectra of compound N2 in $CDCl_3$ (298 K).

Evaluation Only. Created with Aspose.Pdf. Copyright 2002-2014 Aspose Pty Ltd.

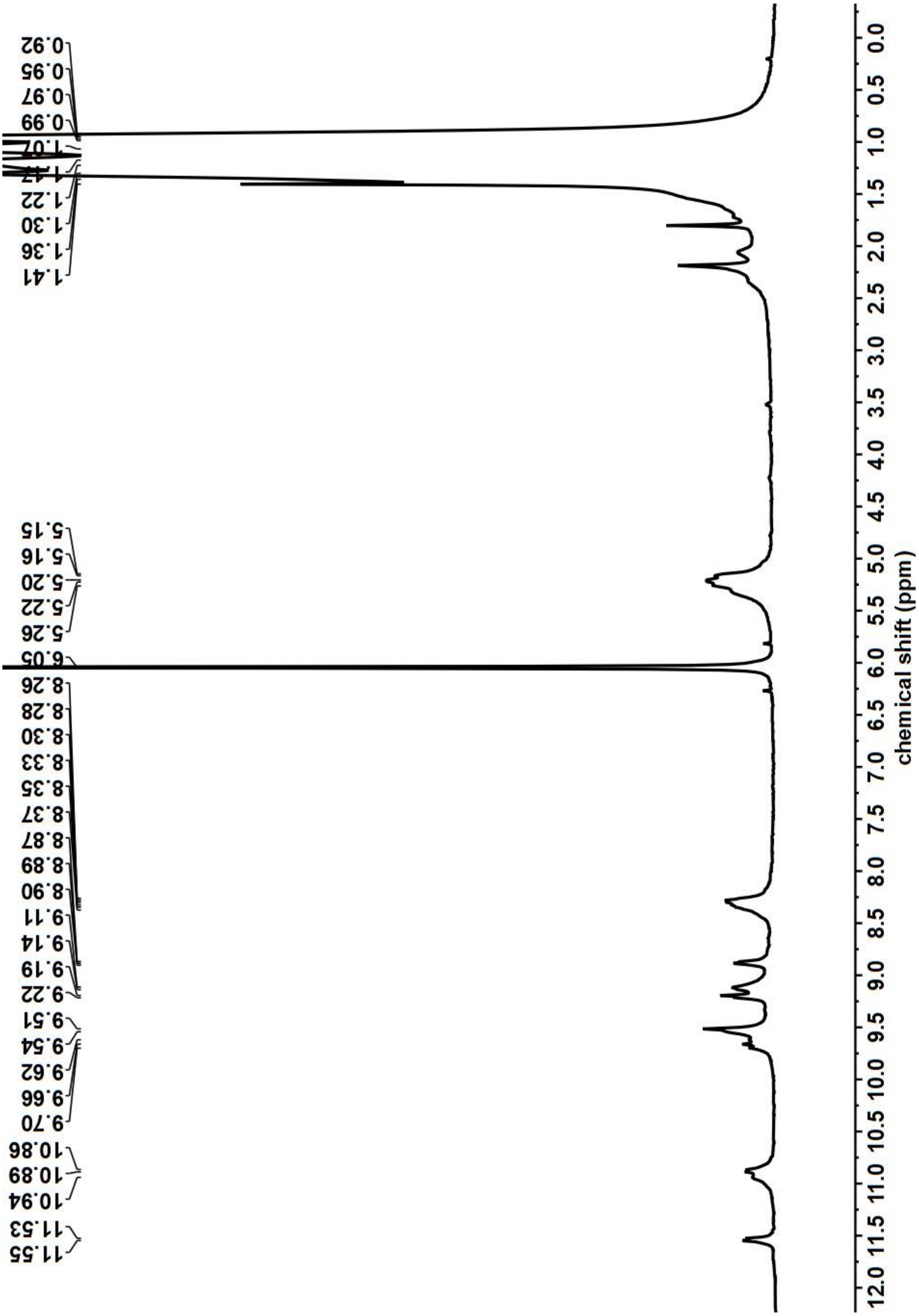

Fig. S38. ^{1}HNMR spectra of P1 in $C_2D_2Cl_4$ (373 K).

Evaluation Only. Created with Aspose.Pdf. Copyright 2002-2014 Aspose Pty Ltd.

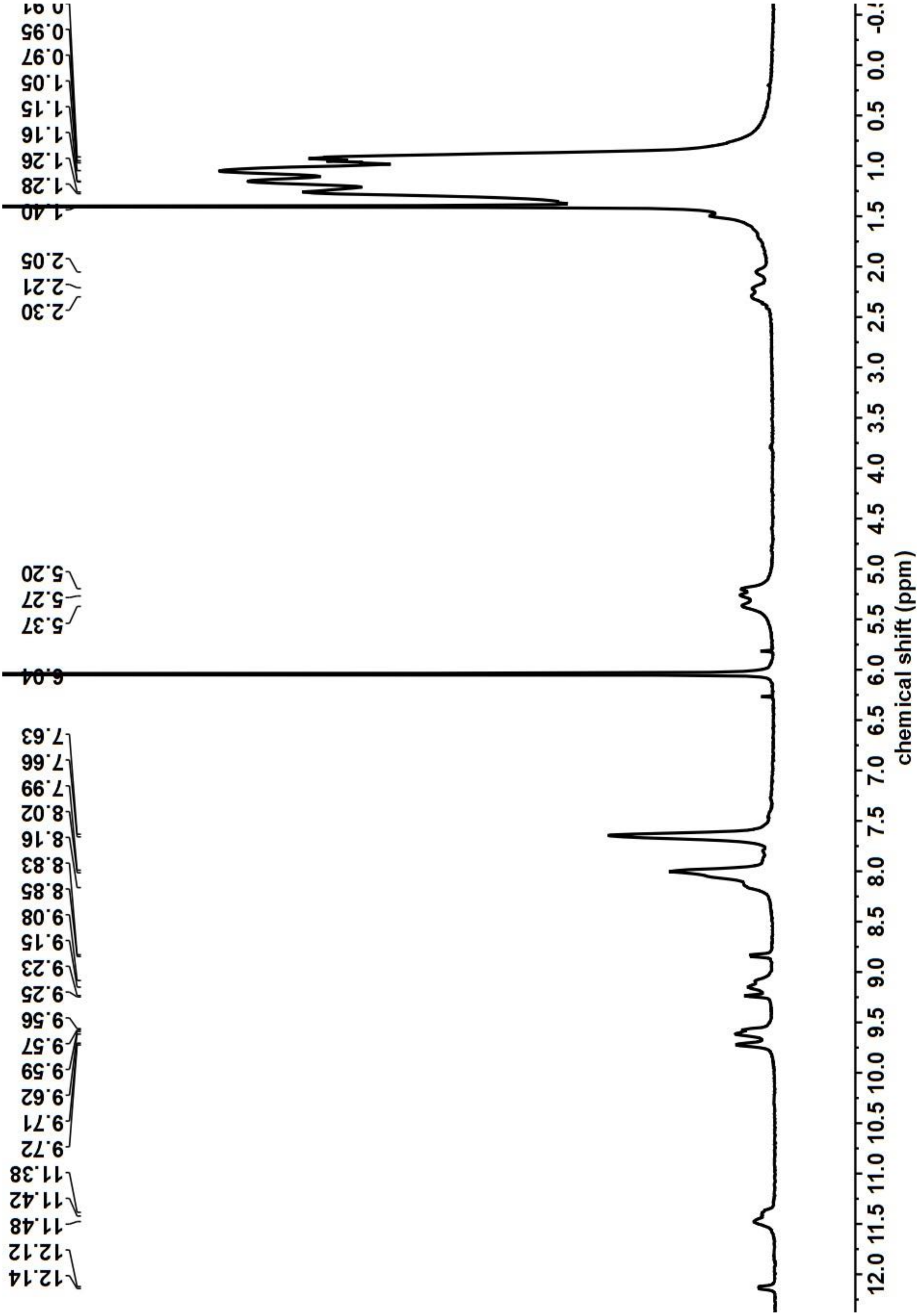

Fig. S39. ^{1}HNMR spectra of P2 in $C_2D_2Cl_4$ (373 K).

Evaluation Only. Created with Aspose.Pdf. Copyright 2002-2014 Aspose Pty Ltd.

S13. Computational Atomic Coordinate

The model structure of N1

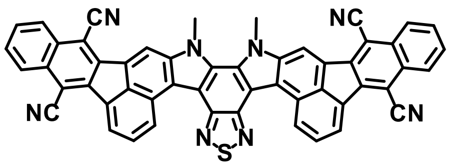

83 atoms
Total energy: -2829.442377 (Hartree)

Table S6. Computational atomic coordinates of ground-state N1 structure calculated at the DFT//B3LYP/def2-SVP level

#	Atom	Coordinates		
		X	Y	Z
1	C	-0.727829	2.504767	-0.093796
2	C	0.727805	2.504776	0.093133
3	C	1.448737	1.260218	0.111828
4	C	0.715568	0.053528	-0.013456
5	C	-0.715562	0.053498	0.012652
6	C	-1.448760	1.260206	-0.112464
7	S	-0.000034	4.807696	-0.000225
8	N	1.237863	3.727185	0.164795
9	N	-1.237906	3.727175	-0.165353
10	C	2.841928	0.914871	0.116452
11	C	4.077299	1.643055	0.237377
12	C	2.896994	-0.498785	-0.060408
13	C	4.274628	3.037094	0.431140
14	C	5.246011	0.857026	0.154907
15	C	4.085246	-1.259690	-0.148389
16	C	5.561293	3.556840	0.529585
17	H	3.405804	3.691416	0.492144
18	C	6.560355	1.387455	0.255774
19	C	5.273369	-0.560989	-0.036591
20	H	4.057232	-2.340206	-0.282424
21	C	6.723203	2.752351	0.445664
22	H	5.681960	4.632434	0.677754
23	H	7.710855	3.206677	0.529401
24	C	-2.841946	0.914867	-0.116785
25	C	-4.077341	1.643063	-0.237419
26	C	-2.896982	-0.498816	0.059951
27	C	-4.274699	3.037115	-0.431046
28	C	-5.246040	0.857030	-0.154842
29	C	-4.085229	-1.259725	0.148018
30	C	-5.561380	3.556871	-0.529247
31	H	-3.405886	3.691444	-0.492124
32	C	-6.560397	1.387463	-0.255469
33	C	-5.273364	-0.561010	0.036487
34	H	-4.057212	-2.340252	0.281956

Evaluation Only. Created with Aspose.Pdf. Copyright 2002-2014 Aspose Pty Ltd.

35	C	-6.723276	2.752378	-0.445210
36	H	-5.682067	4.632480	-0.677299
37	H	-7.710942	3.206710	-0.528759
38	N	1.604526	-0.998711	-0.160263
39	N	-1.604499	-0.998715	0.159700
40	C	7.480898	0.248414	0.119866
41	C	8.864298	0.167500	0.136797
42	C	6.687116	-0.947874	-0.060348
43	C	9.524935	-1.103103	-0.024167
44	C	7.302696	-2.180702	-0.218568
45	C	10.938145	-1.216007	-0.010609
46	C	8.737805	-2.286883	-0.203661
47	C	11.551263	-2.443281	-0.167315
48	H	11.537460	-0.313837	0.126152
49	C	9.396277	-3.532539	-0.362089
50	C	10.774581	-3.610453	-0.344410
51	H	12.641087	-2.511861	-0.154322
52	H	8.794571	-4.433348	-0.498790
53	H	11.266296	-4.577733	-0.467758
54	C	-7.480918	0.248407	-0.119540
55	C	-8.864320	0.167493	-0.136268
56	C	-6.687107	-0.947898	0.060431
57	C	-9.524931	-1.103129	0.024663
58	C	-7.302661	-2.180744	0.218616
59	C	-10.938143	-1.216035	0.011308
60	C	-8.737772	-2.286926	0.203914
61	C	-11.551234	-2.443328	0.167972
62	H	-11.537481	-0.313851	-0.125263
63	C	-9.396217	-3.532602	0.362306
64	C	-10.774523	-3.610517	0.344824
65	H	-12.641061	-2.511908	0.155137
66	H	-8.794488	-4.433424	0.498821
67	H	-11.266217	-4.577811	0.468139
68	C	-9.648637	1.351216	-0.314113
69	N	-10.276143	2.317827	-0.459173
70	C	-6.499923	-3.350710	0.399763
71	N	-5.814838	-4.277879	0.545659
72	C	6.499989	-3.350651	-0.399962
73	N	5.814936	-4.277809	-0.546079
74	C	9.648584	1.351207	0.314887
75	N	10.276065	2.317805	0.460139
76	C	1.333780	-2.294398	-0.765539
77	H	1.380504	-3.116640	-0.036079
78	H	0.345894	-2.281877	-1.237668
79	H	2.077961	-2.487625	-1.551007
80	C	-1.333612	-2.294497	0.764670
81	H	-1.380029	-3.116575	0.035006
82	H	-0.345822	-2.281914	1.237021
83	H	-2.077910	-2.488089	1.549932

Evaluation Only. Created with Aspose.Pdf. Copyright 2002-2014 Aspose Pty Ltd.

The model structure of *cis*-P1

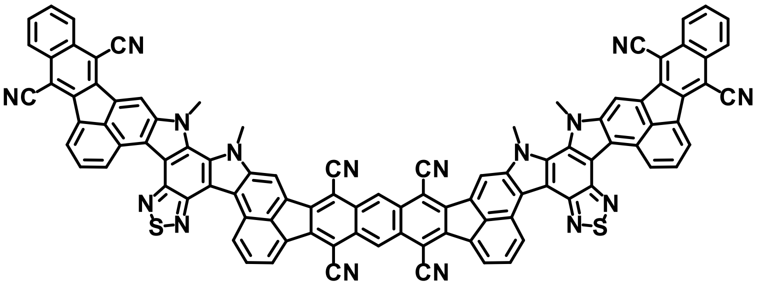

154 atoms
Total energy: -5426.774935 (Hartree)

Table S7. Computational atomic coordinates of ground-state *cis*-P1 structure calculated at the DFT//B3LYP/def2-SVP level

#	Atom	Coordinates		
		X	Y	Z
1	C	10.545983	-2.676511	-0.280861
2	C	11.749228	-1.852826	-0.444908
3	C	11.671200	-0.426546	-0.274882
4	C	10.408798	0.151191	0.011609
5	C	9.211559	-0.631664	-0.035577
6	C	9.265887	-2.047199	-0.095139
7	S	12.404255	-4.166223	-0.691811
8	N	12.837499	-2.572883	-0.683032
9	N	10.791563	-3.975277	-0.391735
10	C	12.646263	0.624729	-0.210764
11	C	14.067397	0.717784	-0.418203
12	C	11.932854	1.799653	0.167846
13	C	14.979697	-0.299847	-0.807944
14	C	14.620203	1.998583	-0.207653
15	C	12.516313	3.069831	0.380927
16	C	16.331250	-0.008925	-0.962887
17	H	14.607212	-1.309907	-0.974321
18	C	16.000463	2.296243	-0.366266
19	C	13.882239	3.159966	0.186233
20	H	11.912492	3.929461	0.668433
21	C	16.868382	1.283278	-0.748474
22	H	17.008917	-0.811587	-1.262728
23	H	17.935480	1.461933	-0.883605
24	C	7.914425	-2.527891	-0.062255
25	C	7.288455	-3.823645	-0.064188
26	C	7.084881	-1.367815	-0.036441
27	C	7.897613	-5.107536	-0.073474
28	C	5.877587	-3.807110	-0.050767
29	C	5.672221	-1.381355	-0.032630
30	C	7.114075	-6.257394	-0.068207
31	H	8.984622	-5.177232	-0.093804
32	C	5.077132	-4.981621	-0.045082
33	C	5.068419	-2.626318	-0.038975

Evaluation Only. Created with Aspose.Pdf. Copyright 2002-2014 Aspose Pty Ltd.

34	H	5.096729	-0.457009	-0.011025
35	C	5.699448	-6.222451	-0.053433
36	H	7.611117	-7.230095	-0.076174
37	H	5.128534	-7.151266	-0.049109
38	N	10.587264	1.489789	0.320277
39	N	7.885307	-0.232375	-0.044546
40	C	16.157006	3.725448	-0.055687
41	C	17.267068	4.555013	-0.042870
42	C	14.853732	4.253608	0.284445
43	C	17.136377	5.946628	0.307975
44	C	14.705750	5.589461	0.626857
45	C	18.254030	6.818679	0.328871
46	C	15.845293	6.468111	0.646176
47	C	18.106979	8.148908	0.668789
48	H	19.237503	6.421047	0.071076
49	C	15.726233	7.838190	0.991181
50	C	16.833574	8.662661	1.002558
51	H	18.978769	8.806511	0.679723
52	H	14.741543	8.233478	1.248724
53	H	16.724863	9.715759	1.270479
54	C	3.678446	-4.533113	-0.030822
55	C	2.485286	-5.230417	-0.021948
56	C	3.676457	-3.078132	-0.028120
57	C	1.223620	-4.523271	-0.010256
58	C	2.483791	-2.377324	-0.018996
59	C	-0.000022	-5.204005	-0.000195
60	C	1.223031	-3.081736	-0.009555
61	H	-0.000025	-6.295826	-0.000345
62	C	-0.000014	-2.400488	0.000185
63	H	-0.000010	-1.308507	0.000334
64	C	2.490335	-6.660610	-0.024617
65	N	2.510468	-7.821815	-0.026880
66	C	2.503879	-0.948067	-0.021388
67	N	2.562827	0.212370	-0.024863
68	C	13.409145	6.090441	0.964371
69	N	12.340720	6.459691	1.233358
70	C	18.555788	4.031662	-0.379442
71	N	19.597173	3.596841	-0.654326
72	C	9.694962	2.333905	1.101356
73	H	9.237462	3.132254	0.498294
74	H	8.907540	1.720863	1.552030
75	H	10.263847	2.801204	1.917487
76	C	7.364395	1.064165	-0.452769
77	H	6.924417	1.622900	0.386588
78	H	8.165514	1.657534	-0.905548
79	H	6.585926	0.916011	-1.214424
80	C	-1.223660	-4.523266	0.010049
81	C	-2.485331	-5.230407	0.021548
82	C	-1.223063	-3.081731	0.009736
83	C	-3.678486	-4.533098	0.030612
84	C	-2.490390	-6.660601	0.023830

Evaluation Only. Created with Aspose.Pdf. Copyright 2002-2014 Aspose Pty Ltd.

85	C	-2.483820	-2.377314	0.019362
86	C	-5.077175	-4.981602	0.044747
87	C	-3.676490	-3.078117	0.028298
88	N	-2.510532	-7.821807	0.025779
89	C	-2.503895	-0.948057	0.022138
90	C	-5.877623	-3.807086	0.050741
91	C	-5.699498	-6.222428	0.052787
92	C	-5.068449	-2.626298	0.039265
93	N	-2.562829	0.212379	0.025909
94	C	-7.288494	-3.823619	0.064193
95	C	-7.114126	-6.257369	0.067574
96	H	-5.128590	-7.151246	0.048208
97	C	-5.672252	-1.381329	0.033232
98	C	-7.914453	-2.527862	0.062636
99	C	-7.897659	-5.107511	0.073154
100	H	-7.611168	-7.230073	0.075281
101	C	-7.084904	-1.367794	0.037057
102	H	-5.096772	-0.456968	0.011889
103	C	-9.265921	-2.047154	0.095704
104	H	-8.984668	-5.177205	0.093473
105	N	-7.885308	-0.232338	0.045466
106	C	-10.546059	-2.676459	0.281198
107	C	-9.211564	-0.631623	0.036452
108	C	-7.364404	1.063984	0.454480
109	C	-11.749300	-1.852756	0.445223
110	N	-10.791710	-3.975236	0.391774
111	C	-10.408747	0.151288	-0.010775
112	H	-6.923888	1.622933	-0.384451
113	H	-8.165678	1.657320	0.907013
114	H	-6.586378	0.915425	1.216517
115	C	-11.671215	-0.426461	0.275409
116	N	-12.837640	-2.572809	0.683029
117	S	-12.404467	-4.166170	0.691522
118	N	-10.587112	1.489920	-0.319335
119	C	-12.646255	0.624803	0.211070
120	C	-11.932752	1.799756	-0.167289
121	C	-9.694530	2.334165	-1.099949
122	C	-14.067453	0.717811	0.418063
123	C	-12.516183	3.069930	-0.380488
124	H	-9.237406	3.132543	-0.496636
125	H	-8.906820	1.721225	-1.550262
126	H	-10.263063	2.801447	-1.916336
127	C	-14.979842	-0.299874	0.807452
128	C	-14.620223	1.998608	0.207425
129	C	-13.882164	3.160031	-0.186165
130	H	-11.912298	3.929588	-0.667776
131	C	-16.331449	-0.008996	0.962009
132	H	-14.607383	-1.309940	0.973859
133	C	-16.000537	2.296225	0.365643
134	C	-14.853650	4.253660	-0.284584
135	C	-16.868546	1.283211	0.747524

Evaluation Only. Created with Aspose.Pdf. Copyright 2002-2014 Aspose Pty Ltd.

136	H	-17.009190	-0.811696	1.261580
137	C	-16.157015	3.725451	0.055125
138	C	-14.705591	5.589543	-0.626849
139	H	-17.935688	1.461831	0.882346
140	C	-17.267088	4.554996	0.042039
141	C	-15.845144	6.468172	-0.646442
142	C	-13.408893	6.090572	-0.963935
143	C	-17.136319	5.946639	-0.308665
144	C	-18.555898	4.031596	0.378192
145	C	-15.726007	7.838279	-0.991307
146	N	-12.340395	6.459855	-1.232582
147	C	-18.253983	6.818670	-0.329829
148	N	-19.597355	3.596733	0.652733
149	C	-16.833360	8.662731	-1.002950
150	H	-14.741249	8.233605	-1.248528
151	C	-18.106854	8.148928	-0.669601
152	H	-19.237524	6.421000	-0.072358
153	H	-16.724589	9.715852	-1.270757
154	H	-18.978654	8.806516	-0.680744

The model structure of *trans*-P1

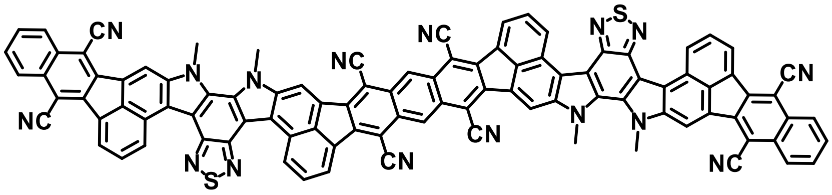

154 atoms

Total energy: -5426.775116 (Hartree)

Table S8. Computational atomic coordinates of ground-state *trans*-P1 structure calculated at the DFT//B3LYP/def2-SVP level

#	Atom	Coordinates		
		X	Y	Z
1	C	-10.114772	3.195379	-0.278682
2	C	-11.548534	2.923139	-0.431803
3	C	-12.047171	1.584780	-0.260341
4	C	-11.119828	0.548958	0.016630
5	C	-9.709569	0.786552	-0.041676
6	C	-9.192705	2.105493	-0.102526
7	S	-11.223679	5.305707	-0.676539
8	N	-12.258942	4.019418	-0.661998
9	N	-9.820226	4.483956	-0.389172
10	C	-13.361292	1.012478	-0.186110
11	C	-14.702236	1.497358	-0.381332
12	C	-13.175541	-0.350469	0.188865
13	C	-15.133292	2.795887	-0.765712

Evaluation Only. Created with Aspose.Pdf. Copyright 2002-2014 Aspose Pty Ltd.

14	C	-15.720374	0.545476	-0.163017
15	C	-14.217506	-1.280383	0.409814
16	C	-16.489386	3.071550	-0.908392
17	H	-14.388525	3.572017	-0.937686
18	C	-17.105516	0.826554	-0.308782
19	C	-15.506580	-0.814860	0.227310
20	H	-14.006654	-2.310411	0.694071
21	C	-17.497721	2.102990	-0.686084
22	H	-16.790922	4.078944	-1.204441
23	H	-18.548055	2.367457	-0.811518
24	C	-7.761732	2.004428	-0.081307
25	C	-6.669054	2.940836	-0.089406
26	C	-7.466215	0.609156	-0.061175
27	C	-6.712892	4.361213	-0.094705
28	C	-5.382938	2.360431	-0.087300
29	C	-6.166468	0.055584	-0.069037
30	C	-5.534331	5.100980	-0.096443
31	H	-7.681119	4.860514	-0.106428
32	C	-4.179043	3.115940	-0.088729
33	C	-5.114484	0.954319	-0.081280
34	H	-6.009347	-1.021927	-0.051499
35	C	-4.252176	4.502200	-0.092900
36	H	-5.600228	6.191353	-0.101099
37	H	-3.356954	5.124521	-0.093711
38	N	-11.817367	-0.606267	0.329310
39	N	-8.654465	-0.110471	-0.061430
40	C	-17.819315	-0.420117	0.007330
41	C	-19.168783	-0.734532	0.033180
42	C	-16.834242	-1.427045	0.337250
43	C	-19.604046	-2.061787	0.387719
44	C	-17.231351	-2.710232	0.682712
45	C	-20.977449	-2.411951	0.422441
46	C	-18.627451	-3.057744	0.715684
47	C	-21.373177	-3.689457	0.766028
48	H	-21.721228	-1.652935	0.172482
49	C	-19.064744	-4.360566	1.064587
50	C	-20.409708	-4.671251	1.089623
51	H	-22.435443	-3.941856	0.787741
52	H	-18.318900	-5.117919	1.314239
53	H	-20.730070	-5.679528	1.360468
54	C	-3.077117	2.144676	-0.085387
55	C	-1.704603	2.305446	-0.086244
56	C	-3.658101	0.810675	-0.082043
57	C	-0.832010	1.151654	-0.084449
58	C	-2.846034	-0.309362	-0.082449
59	C	0.561473	1.284374	-0.084267
60	C	-1.408855	-0.169439	-0.083560
61	H	0.999600	2.284518	-0.084515
62	C	-0.561422	-1.284442	-0.083930
63	H	-0.999550	-2.284586	-0.083915
64	C	-1.134699	3.617112	-0.089267
65	N	-0.683472	4.687326	-0.091667

Evaluation Only. Created with Aspose.Pdf. Copyright 2002-2014 Aspose Pty Ltd.

66	C	-3.438626	-1.610243	-0.084171
67	N	-3.961786	-2.647656	-0.086710
68	C	-16.241469	-3.689504	1.010200
69	N	-15.408361	-4.456433	1.270898
70	C	-20.142428	0.262194	-0.293185
71	N	-20.924428	1.078572	-0.559876
72	C	-11.331667	-1.739341	1.103361
73	H	-11.237176	-2.652420	0.496999
74	H	-10.361057	-1.494352	1.547021
75	H	-12.033556	-1.941842	1.924575
76	C	-8.699791	-1.506706	-0.470841
77	H	-8.514686	-2.195808	0.366476
78	H	-9.674747	-1.728569	-0.917056
79	H	-7.932611	-1.682131	-1.238091
80	C	0.832061	-1.151723	-0.084289
81	C	1.704653	-2.305515	-0.085929
82	C	1.408907	0.169370	-0.083740
83	C	3.077166	-2.144746	-0.085258
84	C	1.134752	-3.617183	-0.088606
85	C	2.846086	0.309291	-0.082808
86	C	4.179090	-3.116011	-0.088506
87	C	3.658154	-0.810745	-0.082251
88	N	0.683524	-4.687398	-0.090724
89	C	3.438675	1.610173	-0.084876
90	C	5.382988	-2.360501	-0.087358
91	C	4.252224	-4.502268	-0.092412
92	C	5.114536	-0.954391	-0.081622
93	N	3.961824	2.647591	-0.087693
94	C	6.669106	-2.940913	-0.089516
95	C	5.534377	-5.101054	-0.095991
96	H	3.357001	-5.124590	-0.092984
97	C	6.166532	-0.055658	-0.069659
98	C	7.761781	-2.004504	-0.081787
99	C	6.712941	-4.361293	-0.094539
100	H	5.600263	-6.191429	-0.100414
101	C	7.466262	-0.609238	-0.061838
102	H	6.009443	1.021863	-0.052306
103	C	9.192768	-2.105559	-0.103295
104	H	7.681166	-4.860596	-0.106249
105	N	8.654500	0.110413	-0.062257
106	C	10.114884	-3.195420	-0.279439
107	C	9.709602	-0.786622	-0.042622
108	C	8.699787	1.506495	-0.472309
109	C	11.548646	-2.923119	-0.432525
110	N	9.820412	-4.484019	-0.389853
111	C	11.119819	-0.548942	0.015723
112	H	8.514260	2.195927	0.364642
113	H	9.674897	1.728291	-0.918213
114	H	7.932882	1.681457	-1.239947
115	C	12.047227	-1.584744	-0.261093
116	N	12.259125	-4.019366	-0.662638
117	S	11.223950	-5.305727	-0.676951

Evaluation Only. Created with Aspose.Pdf. Copyright 2002-2014 Aspose Pty Ltd.

118	N	11.817252	0.606327	0.328440
119	C	13.361306	-1.012429	-0.186569
120	C	13.175455	0.350553	0.188249
121	C	11.331371	1.739425	1.102344
122	C	14.702290	-1.497336	-0.381396
123	C	14.217375	1.280476	0.409381
124	H	11.237352	2.652562	0.495994
125	H	10.360488	1.494581	1.545483
126	H	12.032878	1.941762	1.923930
127	C	15.133418	-2.795913	-0.765528
128	C	15.720376	-0.545433	-0.162948
129	C	15.506491	0.814944	0.227183
130	H	14.006458	2.310515	0.693551
131	C	16.489542	-3.071597	-0.907875
132	H	14.388683	-3.572066	-0.937554
133	C	17.105548	-0.826533	-0.308369
134	C	16.834129	1.427138	0.337357
135	C	17.497830	-2.103015	-0.685443
136	H	16.791145	-4.079031	-1.203724
137	C	17.819276	0.420175	0.007764
138	C	17.231160	2.710365	0.682763
139	H	18.548191	-2.367501	-0.810608
140	C	19.168739	0.734593	0.033886
141	C	18.627252	3.057880	0.716012
142	C	16.241201	3.689673	1.009912
143	C	19.603921	2.061887	0.388378
144	C	20.142457	-0.262169	-0.292149
145	C	19.064466	4.360741	1.064868
146	N	15.408028	4.456622	1.270343
147	C	20.977316	2.412054	0.423374
148	N	20.924516	-1.078580	-0.558568
149	C	20.409424	4.671429	1.090172
150	H	18.318566	5.118122	1.314270
151	C	21.372966	3.689598	0.766906
152	H	21.721151	1.653009	0.173671
153	H	20.729725	5.679738	1.360974
154	H	22.435228	3.942000	0.788831

The model structure of N2

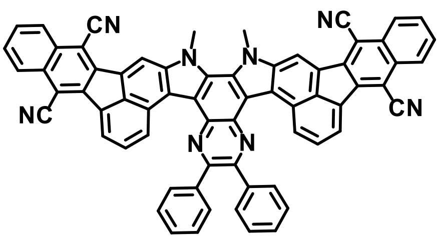

106 atoms

Total energy: -2970.588829 (Hartree)

Evaluation Only. Created with Aspose.Pdf. Copyright 2002-2014 Aspose Pty Ltd.

Table S9. Computational atomic coordinates of ground-state N2 structure calculated at the DFT//B3LYP/def2-SVP level

#	Atom	Coordinates		
		X	Y	Z
1	C	-1.434675	0.103777	-0.100810
2	C	-0.710380	-1.108936	0.023695
3	C	0.710398	-1.108889	-0.024287
4	C	1.434666	0.103824	0.100341
5	C	-2.836539	-0.246439	-0.119184
6	C	-4.084025	0.459569	-0.280405
7	C	-2.887795	-1.658682	0.079245
8	C	-4.303910	1.836630	-0.557230
9	C	-5.247280	-0.333351	-0.168822
10	C	-4.068916	-2.427432	0.188822
11	C	-5.593923	2.339730	-0.682263
12	H	-3.438325	2.485553	-0.662216
13	C	-6.567943	0.179069	-0.295895
14	C	-5.263745	-1.743613	0.068137
15	H	-4.029043	-3.504514	0.345239
16	C	-6.747922	1.530804	-0.551360
17	H	-5.718263	3.404613	-0.893148
18	H	-7.740985	1.968368	-0.657286
19	C	2.836537	-0.246389	0.118793
20	C	4.084008	0.459609	0.280136
21	C	2.887830	-1.658620	-0.079629
22	C	4.303867	1.836741	0.556614
23	C	5.247264	-0.333339	0.168795
24	C	4.068929	-2.427389	-0.189144
25	C	5.593868	2.339865	0.681672
26	H	3.438265	2.485688	0.661362
27	C	6.567913	0.179103	0.295908
28	C	5.263757	-1.743597	-0.068187
29	H	4.029026	-3.504446	-0.345731
30	C	6.747878	1.530892	0.551102
31	H	5.718183	3.404804	0.892291
32	H	7.740934	1.968464	0.657055
33	N	-1.596818	-2.161053	0.177527
34	N	1.596846	-2.161020	-0.177964
35	C	-7.477824	-0.962167	-0.117129
36	C	-8.860558	-1.056971	-0.130037
37	C	-6.673085	-2.143826	0.105230
38	C	-9.508932	-2.327526	0.076064
39	C	-7.276442	-3.376369	0.306008
40	C	-10.921016	-2.454453	0.067945
41	C	-8.710556	-3.496812	0.295893
42	C	-11.522364	-3.681291	0.267921
43	H	-11.529025	-1.563356	-0.099597
44	C	-9.357012	-4.742403	0.498206
45	C	-10.734529	-4.834161	0.484711
46	H	-12.611518	-3.760568	0.258401
47	H	-8.746517	-5.632070	0.665513
48	H	-11.216848	-5.801241	0.642109

Evaluation Only. Created with Aspose.Pdf. Copyright 2002-2014 Aspose Pty Ltd.

49	C	7.477814	-0.962139	0.117281
50	C	8.860548	-1.056941	0.130336
51	C	6.673100	-2.143806	-0.105125
52	C	9.508942	-2.327505	-0.075647
53	C	7.276477	-3.376354	-0.305811
54	C	10.921026	-2.454437	-0.067354
55	C	8.710590	-3.496799	-0.295525
56	C	11.522395	-3.681285	-0.267203
57	H	11.529018	-1.563335	0.100224
58	C	9.357068	-4.742401	-0.497706
59	C	10.734582	-4.834162	-0.484038
60	H	12.611547	-3.760566	-0.257547
61	H	8.746592	-5.632074	-0.665050
62	H	11.216917	-5.801252	-0.641330
63	C	9.657079	0.111681	0.348789
64	N	10.295638	1.065551	0.526642
65	C	6.462895	-4.531983	-0.526527
66	N	5.770543	-5.448374	-0.703035
67	C	-6.462833	-4.531993	0.526650
68	N	-5.770457	-5.448381	0.703082
69	C	-9.657108	0.111643	-0.348466
70	N	-10.295679	1.065506	-0.526315
71	C	-1.319821	-3.447999	0.797467
72	H	-1.390303	-4.281699	0.082767
73	H	-0.319425	-3.434911	1.242584
74	H	-2.044017	-3.624768	1.605613
75	C	1.320066	-3.447685	-0.798637
76	H	1.391582	-4.281807	-0.084536
77	H	0.319316	-3.434852	-1.242922
78	H	2.043699	-3.623481	-1.607522
79	C	0.715014	1.351613	0.082384
80	C	-0.715068	1.351602	-0.082694
81	C	0.704818	3.674196	0.117822
82	C	-0.704936	3.674205	-0.117642
83	N	-1.360876	2.522698	-0.186158
84	N	1.360790	2.522703	0.186084
85	C	-1.513236	4.905445	-0.321875
86	C	-2.760934	5.026951	0.312166
87	C	-1.079893	5.934841	-1.173393
88	C	-3.552813	6.157932	0.108490
89	H	-3.101539	4.226780	0.971153
90	C	-1.876980	7.060443	-1.383140
91	H	-0.117992	5.848614	-1.681003
92	C	-3.112998	7.178235	-0.740218
93	H	-4.516817	6.243259	0.615822
94	H	-1.531744	7.849629	-2.055057
95	H	-3.733187	8.062990	-0.902151
96	C	1.513137	4.905421	0.322026
97	C	2.760700	5.027052	-0.312253
98	C	1.079991	5.934629	1.173881
99	C	3.552620	6.157994	-0.108517
100	H	3.101164	4.227014	-0.971477

Evaluation Only. Created with Aspose.Pdf. Copyright 2002-2014 Aspose Pty Ltd.

101	C	1.877120	7.060191	1.383682
102	H	0.118232	5.848267	1.681729
103	C	3.112989	7.178120	0.740499
104	H	4.516515	6.243436	-0.616036
105	H	1.532051	7.849228	2.055859
106	H	3.733222	8.062833	0.902489

Evaluation Only. Created with Aspose.Pdf. Copyright 2002-2014 Aspose Pty Ltd.

The model structure of *cis*-P2

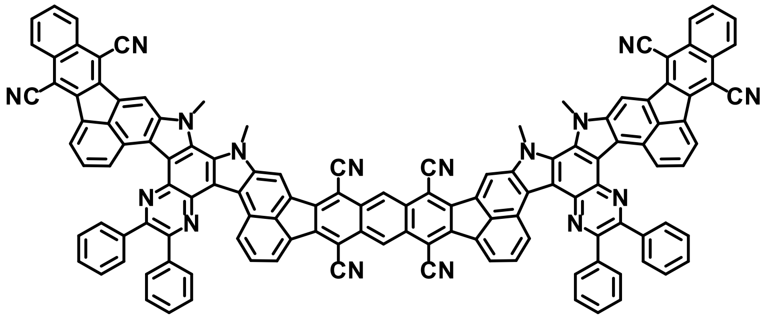

200 atoms
Total energy: -5709.068104 (Hartree)

Table S10. Computational atomic coordinates of ground-state *cis*-P2 structure calculated at the DFT//B3LYP/def2-SVP level

#	Atom	Coordinates		
		X	Y	Z
1	C	11.646693	0.609468	-0.058068
2	C	10.381586	1.177583	0.238232
3	C	9.199617	0.393085	0.147149
4	C	9.279206	-1.019751	0.052010
5	C	12.614675	1.679265	0.022378
6	C	14.030737	1.822075	-0.213038
7	C	11.887385	2.831229	0.447151
8	C	14.958375	0.861918	-0.701709
9	C	14.563638	3.101077	0.059713
10	C	12.448540	4.100834	0.714733
11	C	16.299315	1.189195	-0.867984
12	H	14.593364	-0.134720	-0.935753
13	C	15.934980	3.438184	-0.109246
14	C	13.810965	4.226252	0.521222
15	H	11.828708	4.934658	1.041316
16	C	16.816876	2.473327	-0.574176
17	H	16.978972	0.420783	-1.244156
18	H	17.876337	2.685671	-0.720123
19	C	7.923620	-1.517798	0.083104
20	C	7.291191	-2.814130	0.089790
21	C	7.084710	-0.363024	0.113634
22	C	7.890762	-4.102353	0.133706
23	C	5.879031	-2.799216	0.071669
24	C	5.672329	-0.377410	0.093362
25	C	7.108746	-5.252172	0.135558
26	H	8.975684	-4.164304	0.159225
27	C	5.077073	-3.974378	0.072754
28	C	5.068122	-1.620802	0.065256
29	H	5.097640	0.547395	0.114002
30	C	5.694868	-5.216916	0.100849
31	H	7.610912	-6.222000	0.167742
32	H	5.121141	-6.143977	0.103507
33	N	10.544154	2.507560	0.586143
34	N	7.870929	0.781276	0.137404

Evaluation Only. Created with Aspose.Pdf. Copyright 2002-2014 Aspose Pty Ltd.

35	C	16.068881	4.850547	0.277851
36	C	17.162534	5.700952	0.320747
37	C	14.760879	5.333275	0.663911
38	C	17.009357	7.069048	0.747378
39	C	14.591012	6.645886	1.078076
40	C	18.109726	7.961514	0.800638
41	C	15.713148	7.545555	1.129256
42	C	17.941276	9.268658	1.212638
43	H	19.097204	7.598257	0.509567
44	C	15.571890	8.892773	1.547997
45	C	16.662739	9.738043	1.589238
46	H	18.800086	9.942346	1.247445
47	H	14.583159	9.253597	1.838538
48	H	16.536901	10.773081	1.914042
49	C	3.678658	-3.526471	0.051955
50	C	2.485297	-4.223739	0.035700
51	C	3.676189	-2.071724	0.049312
52	C	1.223660	-3.516534	0.017329
53	C	2.483849	-1.370723	0.032344
54	C	-0.000002	-4.197120	-0.000051
55	C	1.223007	-2.075020	0.016466
56	H	0.000002	-5.288928	-0.000003
57	C	-0.000012	-1.393844	-0.000175
58	H	-0.000016	-0.301860	-0.000223
59	C	2.489487	-5.653906	0.036449
60	N	2.508114	-6.815193	0.037219
61	C	2.503873	0.058593	0.027990
62	N	2.561693	1.219086	0.024575
63	C	13.289665	7.102394	1.457911
64	N	12.218528	7.437390	1.759052
65	C	18.456893	5.223643	-0.059619
66	N	19.503675	4.827243	-0.370023
67	C	9.641565	3.311491	1.396456
68	H	9.192292	4.139439	0.827737
69	H	8.847104	2.676984	1.803011
70	H	10.197228	3.737311	2.244205
71	C	7.346703	2.080290	-0.257064
72	H	6.871823	2.612734	0.580611
73	H	8.155174	2.694876	-0.666511
74	H	6.596004	1.943382	-1.048575
75	C	-1.223668	-3.516545	-0.017489
76	C	-2.485301	-4.223760	-0.035797
77	C	-1.223025	-2.075030	-0.016754
78	C	-3.678667	-3.526502	-0.052111
79	C	-2.489480	-5.653928	-0.036419
80	C	-2.483873	-1.370743	-0.032692
81	C	-5.077080	-3.974420	-0.072856
82	C	-3.676208	-2.071755	-0.049596
83	N	-2.508098	-6.815215	-0.037082
84	C	-2.503906	0.058574	-0.028465
85	C	-5.879045	-2.799263	-0.071882
86	C	-5.694866	-5.216966	-0.100802

Evaluation Only. Created with Aspose.Pdf. Copyright 2002-2014 Aspose Pty Ltd.

87	C	-5.068144	-1.620843	-0.065567
88	N	-2.561733	1.219066	-0.025155
89	C	-7.291206	-2.814189	-0.089972
90	C	-7.108745	-5.252235	-0.135475
91	H	-5.121133	-6.144023	-0.103375
92	C	-5.672362	-0.377458	-0.093743
93	C	-7.923644	-1.517862	-0.083384
94	C	-7.890768	-4.102422	-0.133728
95	H	-7.610906	-6.222071	-0.167532
96	C	-7.084743	-0.363086	-0.114006
97	H	-5.097686	0.547355	-0.114449
98	C	-9.279239	-1.019820	-0.052300
99	H	-8.975692	-4.164383	-0.159194
100	N	-7.870967	0.781210	-0.137881
101	C	-9.199656	0.393010	-0.147481
102	C	-7.346742	2.080269	0.256451
103	C	-10.381628	1.177512	-0.238513
104	H	-6.871873	2.612632	-0.581283
105	H	-8.155217	2.694892	0.665840
106	H	-6.596030	1.943441	1.047963
107	C	-11.646715	0.609420	0.057899
108	N	-10.544213	2.507459	-0.586530
109	C	-12.614678	1.679240	-0.022443
110	C	-11.887418	2.831173	-0.447349
111	C	-9.641801	3.311223	-1.397217
112	C	-14.030702	1.822089	0.213164
113	C	-12.448580	4.100777	-0.714918
114	H	-9.192663	4.139474	-0.828833
115	H	-8.847240	2.676698	-1.803535
116	H	-10.197565	3.736601	-2.245128
117	C	-14.958293	0.861966	0.701987
118	C	-14.563609	3.101096	-0.059556
119	C	-13.810977	4.226234	-0.521221
120	H	-11.828775	4.934564	-1.041647
121	C	-16.299202	1.189279	0.868436
122	H	-14.593270	-0.134673	0.936013
123	C	-15.934922	3.438237	0.109575
124	C	-14.760890	5.333267	-0.663833
125	C	-16.816774	2.473415	0.574659
126	H	-16.978827	0.420895	1.244724
127	C	-16.068848	4.850585	-0.277569
128	C	-14.591053	6.645856	-1.078082
129	H	-17.876212	2.685785	0.720737
130	C	-17.162490	5.701010	-0.320352
131	C	-15.713179	7.545546	-1.129148
132	C	-13.289751	7.102322	-1.458118
133	C	-17.009345	7.069082	-0.747069
134	C	-18.456806	5.223744	0.060218
135	C	-15.571952	8.892741	-1.547972
136	N	-12.218647	7.437288	-1.759412
137	C	-18.109703	7.961568	-0.800218
138	N	-19.503551	4.827380	0.370789

Evaluation Only. Created with Aspose.Pdf. Copyright 2002-2014 Aspose Pty Ltd.

139	H	-14.583253	9.253531	-1.838667
140	H	-19.097148	7.598343	-0.508994
141	C	-16.662790	9.738031	-1.589102
142	C	-17.941284	9.268689	-1.212303
143	C	10.566993	-1.635135	-0.144033
144	C	11.742615	-0.813328	-0.262852
145	C	11.865440	-3.531453	-0.480726
146	C	13.019983	-2.710267	-0.669866
147	N	12.921170	-1.394086	-0.532880
148	N	10.687013	-2.966919	-0.249234
149	C	14.360165	-3.234804	-1.043509
150	C	15.503555	-2.732739	-0.399972
151	C	14.516500	-4.190394	-2.060850
152	C	16.774546	-3.184765	-0.757566
153	H	15.384951	-1.987381	0.388018
154	C	15.788478	-4.634121	-2.423447
155	H	13.638010	-4.580710	-2.576836
156	C	16.920681	-4.136988	-1.770827
157	H	17.654380	-2.792661	-0.241719
158	H	15.896360	-5.371136	-3.222583
159	H	17.915409	-4.489807	-2.053078
160	C	11.891043	-5.017979	-0.500171
161	C	10.883882	-5.718734	-1.184343
162	C	12.875385	-5.743422	0.190698
163	C	10.867638	-7.114231	-1.187210
164	H	10.116213	-5.157401	-1.719310
165	C	12.852117	-7.138324	0.194625
166	H	13.657289	-5.211986	0.735270
167	C	11.851934	-7.828243	-0.497179
168	H	10.083898	-7.646241	-1.731885
169	H	13.618635	-7.690150	0.743608
170	H	11.838720	-8.920689	-0.496831
171	C	-11.742615	-0.813366	0.262787
172	C	-10.567015	-1.635185	0.143870
173	C	-13.019973	-2.710282	0.669934
174	C	-11.865448	-3.531483	0.480723
175	N	-10.687038	-2.966966	0.249119
176	N	-12.921159	-1.394107	0.532908
177	C	-11.891048	-5.018006	0.500284
178	C	-10.883844	-5.718705	1.184447
179	C	-12.875406	-5.743502	-0.190509
180	C	-10.867584	-7.114203	1.187398
181	H	-10.116152	-5.157330	1.719336
182	C	-12.852122	-7.138404	-0.194351
183	H	-13.657332	-5.212107	-0.735089
184	C	-11.851902	-7.828268	0.497456
185	H	-10.083809	-7.646169	1.732065
186	H	-13.618652	-7.690275	-0.743271
187	H	-11.838674	-8.920715	0.497168
188	C	-14.360112	-3.234798	1.043748
189	C	-15.503570	-2.732745	0.400323
190	C	-14.516338	-4.190350	2.061142

Evaluation Only. Created with Aspose.Pdf. Copyright 2002-2014 Aspose Pty Ltd.

191	C	-16.774525	-3.184748	0.758077
192	H	-15.385048	-1.987417	-0.387707
193	C	-15.788278	-4.634057	2.423895
194	H	-13.637792	-4.580655	2.577041
195	C	-16.920552	-4.136936	1.771386
196	H	-17.654414	-2.792655	0.242316
197	H	-15.896077	-5.371042	3.223068
198	H	-17.915251	-4.489738	2.053763
199	H	-16.536977	10.773051	-1.913972
200	H	-18.800085	9.942392	-1.247022

The model structure of *trans*-P2

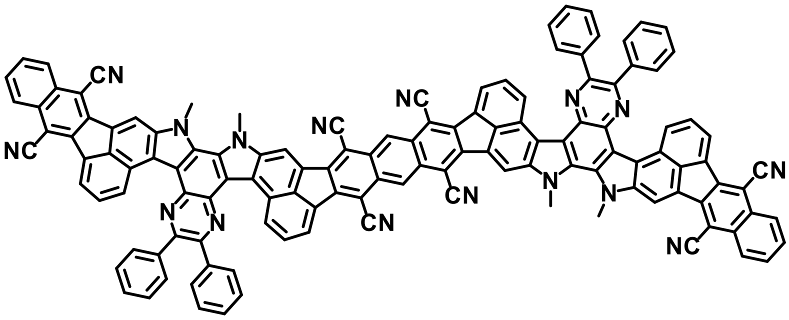

200 atoms

Total energy: -5709.068211 (Hartree)

Table S11. Computational atomic coordinates of ground-state *trans*-P2 structure calculated at the DFT//B3LYP/def2-SVP level

#	Atom	Coordinates		
		X	Y	Z
1	C	-12.127279	0.330686	-0.079803
2	C	-11.101596	-0.596142	0.236508
3	C	-9.734785	-0.212422	0.161823
4	C	-9.376005	1.156200	0.063091
5	C	-13.377756	-0.390158	-0.012068
6	C	-14.765956	-0.093332	-0.268689
7	C	-13.044772	-1.706970	0.425595
8	C	-15.347604	1.101964	-0.772858
9	C	-15.669134	-1.145684	-0.002154
10	C	-13.971942	-2.741688	0.686748
11	C	-16.721808	1.200440	-0.959273
12	H	-14.691437	1.937377	-1.002632
13	C	-17.075225	-1.047272	-0.191799
14	C	-15.304324	-2.444533	0.472703
15	H	-13.642329	-3.723386	1.024026
16	C	-17.612208	0.138537	-0.671713
17	H	-17.127763	2.137899	-1.346924
18	H	-18.683560	0.260161	-0.833732
19	C	-7.933620	1.214869	0.113381
20	C	-6.934268	2.254884	0.127397

Evaluation Only. Created with Aspose.Pdf. Copyright 2002-2014 Aspose Pty Ltd.

21	C	-7.489695	-0.141263	0.158076
22	C	-7.110497	3.665118	0.160221
23	C	-5.594613	1.807606	0.130268
24	C	-6.140807	-0.560739	0.158369
25	C	-6.013691	4.519873	0.172062
26	H	-8.124457	4.056823	0.169028
27	C	-4.471116	2.680326	0.141624
28	C	-5.184044	0.437272	0.137268
29	H	-5.877835	-1.617064	0.188658
30	C	-4.678454	4.052592	0.158700
31	H	-6.194731	5.597126	0.195053
32	H	-3.848177	4.759128	0.168600
33	N	-11.669069	-1.809921	0.584247
34	N	-8.589133	-0.989241	0.172098
35	C	-17.641186	-2.348195	0.195204
36	C	-18.943376	-2.822190	0.222011
37	C	-16.549873	-3.206156	0.602458
38	C	-19.223180	-4.168713	0.652965
39	C	-16.796652	-4.504953	1.021461
40	C	-20.544774	-4.680731	0.689863
41	C	-18.141176	-5.017076	1.056209
42	C	-20.791142	-5.974007	1.106380
43	H	-21.368998	-4.034231	0.382475
44	C	-18.425819	-6.340103	1.479172
45	C	-19.723713	-6.810191	1.504136
46	H	-21.815503	-6.351929	1.128353
47	H	-17.599654	-6.984715	1.785966
48	H	-19.925962	-7.831944	1.832387
49	C	-3.277260	1.825184	0.141953
50	C	-1.927566	2.122827	0.142502
51	C	-3.720842	0.439683	0.140814
52	C	-0.943550	1.062421	0.142827
53	C	-2.800718	-0.593276	0.141183
54	C	0.429669	1.334135	0.142749
55	C	-1.384703	-0.309911	0.142491
56	H	0.765311	2.373161	0.142723
57	C	-0.429625	-1.334179	0.142717
58	H	-0.765267	-2.373205	0.142669
59	C	-1.491498	3.484815	0.141840
60	N	-1.148790	4.594602	0.141066
61	C	-3.259490	-1.947202	0.137353
62	N	-3.674475	-3.032431	0.133671
63	C	-15.703604	-5.335841	1.422763
64	N	-14.791238	-5.980974	1.741544
65	C	-20.023393	-1.973515	-0.179458
66	N	-20.893590	-1.277259	-0.506910
67	C	-11.067749	-2.847028	1.409167
68	H	-10.886065	-3.776164	0.848426
69	H	-10.122779	-2.484147	1.826756
70	H	-11.738916	-3.076963	2.249058
71	C	-8.483088	-2.389138	-0.211444
72	H	-8.206568	-3.035903	0.634659

Evaluation Only. Created with Aspose.Pdf. Copyright 2002-2014 Aspose Pty Ltd.

73	H	-9.435124	-2.728762	-0.632473
74	H	-7.715222	-2.494411	-0.991207
75	C	0.943594	-1.062465	0.142844
76	C	1.927610	-2.122869	0.142539
77	C	1.384746	0.309867	0.142540
78	C	3.277304	-1.825226	0.142036
79	C	1.491544	-3.484858	0.141846
80	C	2.800760	0.593234	0.141282
81	C	4.471160	-2.680368	0.141733
82	C	3.720886	-0.439725	0.140926
83	N	1.148839	-4.594645	0.141049
84	C	3.259531	1.947159	0.137491
85	C	5.594655	-1.807647	0.130415
86	C	4.678502	-4.052634	0.158821
87	C	5.184088	-0.437312	0.137428
88	N	3.674515	3.032389	0.133838
89	C	6.934309	-2.254924	0.127575
90	C	6.013740	-4.519913	0.172227
91	H	3.848226	-4.759171	0.168696
92	C	6.140852	0.560698	0.158584
93	C	7.933658	-1.214913	0.113594
94	C	7.110545	-3.665155	0.160409
95	H	6.194783	-5.597165	0.195242
96	C	7.489739	0.141220	0.158324
97	H	5.877882	1.617023	0.188890
98	C	9.376042	-1.156254	0.063305
99	H	8.124506	-4.056857	0.169246
100	N	8.589181	0.989189	0.172400
101	C	9.734828	0.212362	0.162022
102	C	8.483141	2.389113	-0.211046
103	C	11.101639	0.596074	0.236704
104	H	8.206561	3.035811	0.635091
105	H	9.435198	2.728780	-0.631990
106	H	7.715318	2.494432	-0.990846
107	C	12.127322	-0.330733	-0.079664
108	N	11.669100	1.809840	0.584504
109	C	13.377789	0.390161	-0.012016
110	C	13.044792	1.706941	0.425741
111	C	11.067835	2.846808	1.409647
112	C	14.765979	0.093422	-0.268797
113	C	13.971941	2.741672	0.686906
114	H	10.886294	3.776096	0.849111
115	H	10.122798	2.483933	1.827080
116	H	11.738968	3.076468	2.249645
117	C	15.347636	-1.101810	-0.773106
118	C	15.669136	1.145796	-0.002273
119	C	15.304317	2.444590	0.472726
120	H	13.642311	3.723324	1.024303
121	C	16.721828	-1.200214	-0.959653
122	H	14.691483	-1.937229	-1.002892
123	C	17.075216	1.047455	-0.192044
124	C	16.549848	3.206249	0.602447

Evaluation Only. Created with Aspose.Pdf. Copyright 2002-2014 Aspose Pty Ltd.

125	C	17.612208	-0.138296	-0.672090
126	H	17.127789	-2.137624	-1.347415
127	C	17.641159	2.348367	0.195025
128	C	16.796612	4.505018	1.021545
129	H	18.683551	-0.259861	-0.834211
130	C	18.943334	2.822409	0.221765
131	C	18.141120	5.017189	1.056228
132	C	15.703565	5.335829	1.423010
133	C	19.223123	4.168904	0.652818
134	C	20.023349	1.973810	-0.179872
135	C	18.425747	6.340189	1.479288
136	N	14.791202	5.980903	1.741918
137	C	20.544701	4.680968	0.689653
138	N	20.893543	1.277616	-0.507461
139	H	17.599583	6.984741	1.786209
140	H	21.368923	4.034527	0.382139
141	C	19.723626	6.810323	1.504188
142	C	20.791053	5.974217	1.106267
143	C	11.779410	-1.713057	-0.288612
144	C	10.410158	-2.135550	-0.153432
145	C	12.407450	-3.907487	-0.719447
146	C	11.059489	-4.336286	-0.514366
147	N	10.114380	-3.439234	-0.263299
148	N	12.719088	-2.625410	-0.577967
149	C	13.516573	-4.815127	-1.115484
150	C	14.767870	-4.693238	-0.488657
151	C	13.357480	-5.764674	-2.138010
152	C	15.833463	-5.510819	-0.867787
153	H	14.895075	-3.953525	0.303309
154	C	14.426506	-6.574520	-2.522231
155	H	12.394288	-5.862380	-2.641042
156	C	15.665801	-6.453975	-1.886190
157	H	16.798433	-5.411714	-0.364837
158	H	14.291566	-7.302776	-3.325278
159	H	16.500076	-7.092826	-2.185337
160	C	10.627719	-5.758906	-0.538259
161	C	9.445644	-6.112353	-1.210004
162	C	11.351135	-6.756082	0.136119
163	C	9.002631	-7.435761	-1.217106
164	H	8.880155	-5.338827	-1.732017
165	C	10.901664	-8.076797	0.136006
166	H	12.265171	-6.493726	0.670971
167	C	9.729545	-8.422066	-0.543604
168	H	8.086727	-7.698074	-1.752155
169	H	11.469201	-8.840944	0.672127
170	H	9.382344	-9.457955	-0.546609
171	C	-11.779384	1.713007	-0.288716
172	C	-10.410135	2.135496	-0.153603
173	C	-12.407476	3.907433	-0.719471
174	C	-11.059500	4.336237	-0.514465
175	N	-10.114373	3.439188	-0.263470
176	N	-12.719096	2.625355	-0.577988

Evaluation Only. Created with Aspose.Pdf. Copyright 2002-2014 Aspose Pty Ltd.

177	C	-10.627717	5.758852	-0.538420
178	C	-9.445625	6.112243	-1.210156
179	C	-11.351107	6.756066	0.135935
180	C	-9.002585	7.435646	-1.217311
181	H	-8.880135	5.338682	-1.732120
182	C	-10.901612	8.076768	0.135770
183	H	-12.265141	6.493738	0.670803
184	C	-9.729488	8.421991	-0.543861
185	H	-8.086668	7.697915	-1.752357
186	H	-11.469130	8.840949	0.671864
187	H	-9.382270	9.457874	-0.546906
188	C	-13.516648	4.815057	-1.115411
189	C	-13.357731	5.764500	-2.138061
190	C	-14.767848	4.693188	-0.488392
191	C	-14.426825	6.574302	-2.522181
192	H	-12.394623	5.862157	-2.641263
193	C	-15.833508	5.510731	-0.867419
194	H	-14.894924	3.953527	0.303643
195	C	-15.666014	6.453807	-1.885922
196	H	-14.292027	7.302480	-3.325323
197	H	-16.798400	5.411653	-0.364313
198	H	-16.500343	7.092621	-2.184995
199	H	19.925863	7.832054	1.832515
200	H	21.815402	6.352175	1.128190

Evaluation Only. Created with Aspose.Pdf. Copyright 2002-2014 Aspose Pty Ltd.

S14. Reference

1 M. A. Niyas, R. Ramakrishnan, V. Vijay, E. Sebastian, M. Hariharan, *J. Am. Chem. Soc.* **2019**, *141*, 4536-4540.
2 S. Seifert, D. Schmidt, K. Shoyama, F. Würthner, *Angew. Chem. Int. Ed.* **2017**, *56*, 7595-7600.
3 O.V. Dolomanov, L.J. Bourhis, R. J. Gildea, J. A. K. Howard, H. Puschmann, *J. Appl. Cryst.*, **2009**, *42*, 339–341.
4 G. M. Sheldrick. *Acta Crystallogr. C* **2015**, *71*, 3-8.
5 D. Kratzert, J. J. Holstein, I. Krossing. *J. Appl. Cryst.* **2015**, *48*, 933-938.
6 A. D. Becke. *J. Chem. Phys.* **1993**, *98*, 5648-5652.
7 C. Lee, W. Yang, R. G. Parr. *Phys. Rev. B* **1988**, *37*, 785-789.
8 S. Grimme. *J. Comput. Chem.* **2006**, *27*, 1787-1799.
9 J. Tomasi, B. Mennucci, R. Cammi, *Chem. Rev.*, **2005**, *105*, 2999–3093.
10 Y. Zhao, D. G. Truhlar. *Theor. Chem. Acc.* **2008**, *120*, 215-241.
11 T. Lu, F. Chen. *J. Comput. Chem.* **2012**, *33*, 580-592.
12 T. Lu, F. Chen. *J. Mol. Graph. Model.* **2012**, *38*, 314-323.
13 A. Babel, S. A. Jenekhe, *J. Am. Chem. Soc.* **2003**, *125*, 13656-13657.
14 S. M. West, D. K. Tran, J. Guo, S. E. Chen, D. S. Ginger, S. A. Jenekhe, *Macromolecules* **2023**, *56*, 10222-10235.
15 M. M. Durban, P. D. Kazarinoff, Y. Segawa, C. K. Luscombe, *Macromolecules* **2011**, *44*, 4721-4728.
16 S. R. Bheemireddy, M. P. Hautzinger, T. Li, B. Lee, K. N. Plunkett, *J. Am. Chem. Soc.* **2017**, *139*, 5801-5807.
17 S. M. West, D. K. Tran, J. Guo, S. E. Chen, D. S. Ginger, S. A. Jenekhe, *Macromolecules* **2023**, *56*, 2081-2091.
